# Hospital performance evaluation indicators: a scoping review

**DOI:** 10.1186/s12913-024-10940-1

**Published:** 2024-05-01

**Authors:** Shirin Alsadat Hadian, Reza Rezayatmand, Nasrin Shaarbafchizadeh, Saeedeh Ketabi, Ahmad Reza Pourghaderi

**Affiliations:** 1 Student Research Committee, School of Management and Medical Information Sciences, Isfahan University of Medical Sciences, Isfahan, Iran; 2 Health Management and Economics Research Center, Isfahan University of Medical Sciences, Isfahan, Iran; 3 Hospital Management Research Center, Health Management Research Institute, Iran University of Medical Sciences, Tehran, Iran; 4 Department of Management, Faculty of Administrative Sciences and Economics, University of Isfahan, Isfahan, Iran; 5 School of Public Health and Preventive Medicine, Monash University, Victoria, Australia

**Keywords:** Hospital, Performance evaluation, Indicators

## Abstract

**Background:**

Hospitals are the biggest consumers of health system budgets and hence measuring hospital performance by quantitative or qualitative accessible and reliable indicators is crucial. This review aimed to categorize and present a set of indicators for evaluating overall hospital performance.

**Methods:**

We conducted a literature search across three databases, i.e., PubMed, Scopus, and Web of Science, using possible keyword combinations. We included studies that explored hospital performance evaluation indicators from different dimensions.

**Results:**

We included 91 English language studies published in the past 10 years. In total, 1161 indicators were extracted from the included studies. We classified the extracted indicators into 3 categories, 14 subcategories, 21 performance dimensions, and 110 main indicators. Finally, we presented a comprehensive set of indicators with regard to different performance dimensions and classified them based on what they indicate in the production process, i.e., input, process, output, outcome and impact.

**Conclusion:**

The findings provide a comprehensive set of indicators at different levels that can be used for hospital performance evaluation. Future studies can be conducted to validate and apply these indicators in different contexts. It seems that, depending on the specific conditions of each country, an appropriate set of indicators can be selected from this comprehensive list of indicators for use in the performance evaluation of hospitals in different settings.

**Supplementary Information:**

The online version contains supplementary material available at 10.1186/s12913-024-10940-1.

## Background

Healthcare is complex [1] and a key sector [2] that is now globally faced with problems of rising costs, lack of service efficiency, competition, and equity as well as responsiveness to users [3]. One estimate by the WHO has shown a yearly waste of approximately 20–40% of total healthcare resources because of inefficiency [4]. European countries have spent on average 9.6% of their gross domestic product (GDP) on healthcare in 2017 and 9.92% in 2019. Germany, France, and Sweden reported the highest healthcare expenditures in Europe in 2018 (between 10.9% and 11.5% of GDP) [5]. In the U.S., healthcare spending consumes 18% of the GDP, which is likely to eclipse $6 trillion by 2027 [6].

Hospitals, as the biggest consumers of health system budgets [7], are the major part of the health system [8]. In many countries 50–80% of the health sector budget is dedicated to hospitals [8, 9]. As a result, hospital performance analysis is becoming a routine task for every hospital manager. On the one hand, hospital managers worldwide are faced with difficult decisions regarding cost reduction, increasing service efficiency, and equity [10]. On the other hand, measuring hospital efficiency is an issue of interest among researchers because patients demand high-quality care at lower expenses [11].

To address the above mentioned need to measure hospital performance, implementing an appropriate hospital performance evaluation system is crucial in any hospital. In doing so, hospital administrators use various tools to analyse and monitor hospital activities [1], which need well-defined objectives, standards and quantitative indicators [12]. The latter are used to evaluate care provided to patients both quantitatively and qualitatively and are often related to input, output, processes, and outcomes. These indicators can be used for continuous quality improvement by monitoring, benchmarking, and prioritizing activities [13]. These parameters are developed to improve health outcomes and to provide comparative information for monitoring and managing and formulating policy objectives within and across health services [12]. Studies thus far have used their own set of indicators while evaluating hospital performance, which could be context dependent. In addition, those studies have mostly used a limited set of indicators that focus on few dimensions (2–6 dimensions) of hospital performance [14–18].

Therefore, comprehensive knowledge of potential indicators that can be used for hospital performance evaluation is necessary. It would help choose appropriate indicators when evaluating hospital performance in different contexts. It would also help researchers extend the range of analysis to evaluate performance from a wider perspective by considering more dimensions of performance. Although performance is a very commonly used term, it has several definitions [19, 20], yet, it is often misunderstood [21]. Therefore, some researchers have expressed confusion about the related terms and considered them interchangeable. These terms are effectiveness, efficiency, productivity, quality, flexibility, creativity, sustainability, evaluation, and piloting [21–23]. Thus, this scoping review aimed to categorize and present a comprehensive set of indicators that can be used as a suitable set for hospital performance evaluation at any needed level of analysis, i.e., clinical, para-clinical, logistical, or departmental, and relate those indicators to the appropriate performance dimensions. The uniqueness of this paper is that it provides its readers with a comprehensive collection of indicators that have been used in different performance analysis studies.

## Materials and methods

We conducted a scoping review of a body of literature. The scoping review can be of particular use when the topic has not yet been extensively reviewed or has a complex or heterogeneous nature. This type of review is commonly undertaken to examine the extent, range, and nature of research activity in a topic area; determine the value and potential scope and cost of undertaking a full systematic review; summarize and disseminate research findings; and identify research gaps in the existing literature. As a scoping review provides a rigorous and transparent method for mapping areas of research, it can be used as a standalone project or as a preliminary step to a systematic review [24]. While a systematic review (qualitative or quantitative) usually addresses a narrow topic/scope and is a method for integrating or comparing findings from previous studies [25].

In our study, we used the Preferred Reporting Items for Systematic reviews and Meta-Analyses extension for Scoping Reviews (PRISMA-ScR) Checklist following the methods outlined by Arksey and O’Malley [26] and Tricco [27]. A systematic search for published and English-language literature on hospital performance evaluation models was conducted, using three databases, i.e., PubMed, Scopus, and Web of Science, from 2013 to January 2023. Initially, the identified keywords were refined and validated by a team of experts. Then, a combination of vocabularies was identified by the authors through a brainstorming process. The search strategy was formulated using Boolean operators. The title and abstract of the formulas were searched in the online databases. The search query for each database is presented in Table 1.

**Table 1:** Database query

Database	Search strategy
PubMed	(hospitals[mesh] OR hospital*[tiab]) AND (Performance[tiab] OR productivity[tiab] OR Efficiency[mesh] OR efficiency[tiab] OR effectiveness[tiab]) AND (assessment[tiab] OR evaluat*[tiab] OR Benchmarking[mesh] OR Benchmarking[tiab]) AND (indicator*[tiab]) AND 2013/01/01:2023/01/30[dp] AND english[language]
Scopus	TITLE-ABS((hospital*) AND (performance OR productivity OR efficiency OR effectiveness) AND (assessment OR evaluat* OR benchmarking) AND (indicator*)) AND (PUBYEAR > 2012 AND PUBYEAR < 2024) AND LANGUAGE(english)
Web of Science	TS=((hospital*) AND (performance OR productivity OR efficiency OR effectiveness) AND (assessment OR evaluat* OR benchmarking) AND (indicator*)) AND PY=(2013–2023) AND LA=(english)

In the screening process, relevant references related to hospital performance evaluation were screened and abstracted into researcher-developed Microsoft® Excel forms by dual independent reviewers and conflicting information was provided by other reviewers.

The inclusion criteria were as follows: focused only on the hospital setting, available full text and written in English. We excluded studies that focused on health organization indicators, not specifically on hospital indicators; articles without appropriate data (only focused on models and not indicators; or qualitative checklist questionnaires); and articles that focused only on clinical or disease-related indicators, not hospital performance dimensions, and provided very general items as indicators, not the domains of the indicators themselves. Then, a PRISMA-ScR Checklist was used to improve transparency in our review [28].

To extract the data, researcher-developed Microsoft® Excel forms (data tables) were designed. The following data were subsequently extracted into Microsoft®Excel for synthesis and evaluation: title, author, article year, country, indicator category, study environment (number of hospitals studied), study time frame, indicator name, number of indicators, indicator level (hospital level, department level), evaluation perspective (performance, productivity, efficiency, effectiveness, quality, cost, safety, satisfaction, etc.*)*, study type (quantitative or qualitative), indicator subtype (input (structure), process, output (result), outcome and impact), and other explanations. To create a descriptive summary of the results that address the objectives of this scoping review, numerical summarization was also used.

The purpose of creating the main category and the evaluation perspective section was to develop them and create new categories, which focused on the type of indicators related to the performance term. For example, in the “Category” section, the names of the departments or wards of the hospital (such as hospital laboratories, pharmacies, clinical departments, and warehouses) and in the “Evaluation perspective” section, various terms related to the evaluation of hospital performance were extracted. These two types were used after extracting their information under the title “performance dimension”.

The indicators’ levels were collected to determine the level of performance evaluation with the relevant index. Some indicators were used to evaluate the performance of the entire hospital, some were used to evaluate the performance of hospital departments, and some were used to evaluate the performance at the level of a specific project. For example, several indicators (such as bed occupancy ratio, length of stay, and waiting time) were used to evaluate the performance of the entire hospital, and other indicators (such as laboratory department indicators, energy consumption indicators, and neonatal department indicators) were used only to measure the performance of specific departments. This sections were used under the title “category”. The “category” and “indicator’s name” sections were defined according to the results of the “subcategory” section.

The subtypes of indicators (input (structure), process, output(result), outcome and impact) were defined based on the chain model, and each of the selected indicators was linked to it (Appendix 1). As a result of the chain model, inputs were used to carry out activities, activities led to the delivery of services or products (outputs). The outputs started to bring about change (outcomes), and eventually, this (hopefully) contributed to the impact [29]. The classification of the set of input, process, output, outcome and impact indicators was such that readers could access these categories if necessary according to their chosen evaluation models. The term was used under the title “Indicators by types”.

The type of study was considered quantitative or qualitative for determining whether an indicator was able to perform calculations. In this way, readers can choose articles that use quantitative or qualitative indicators to evaluate hospital performance.

## Results

**Fig. 1:**
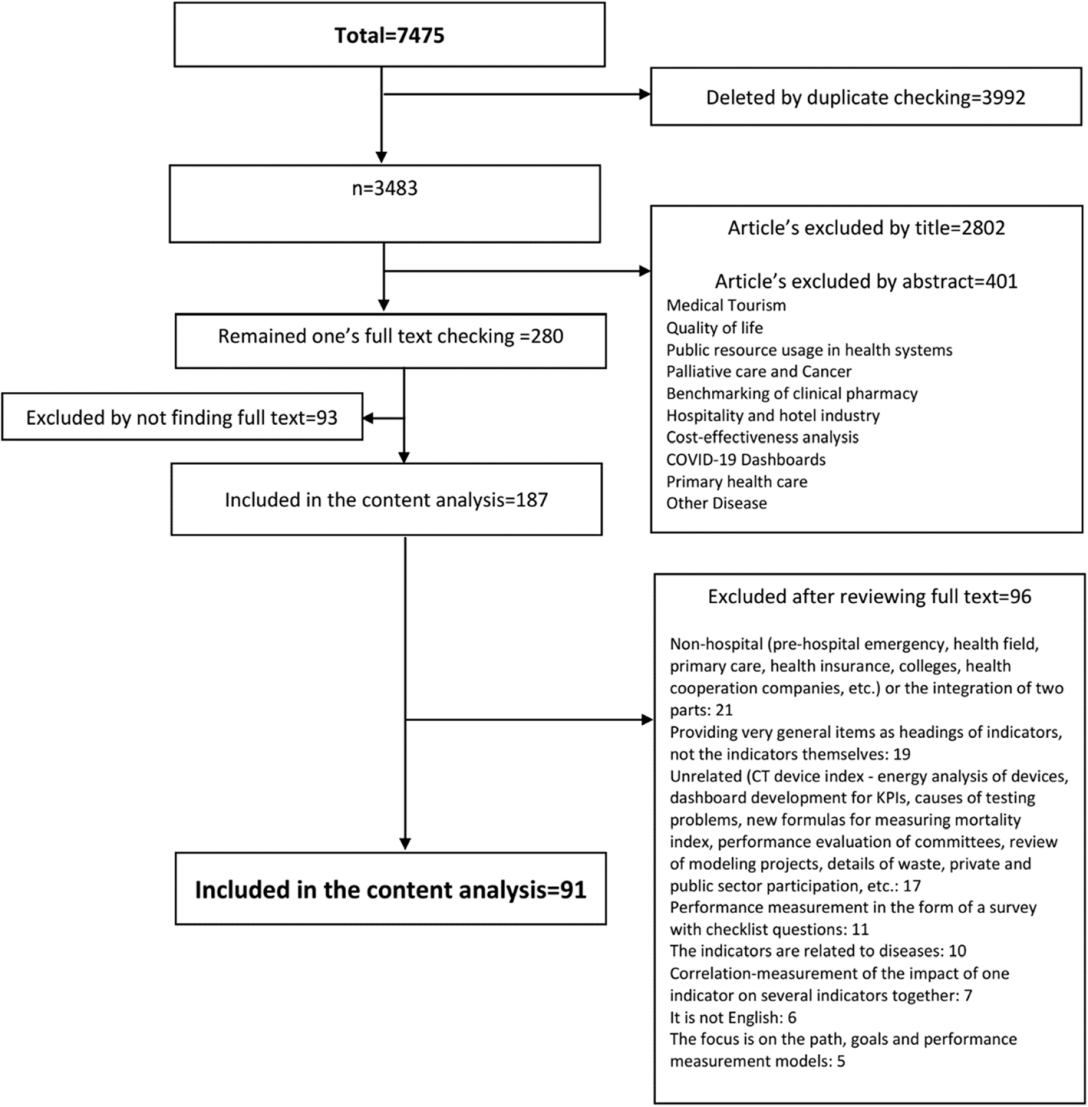
Study selection and data abstraction

We included 91 full-text studies (out of 7475) in English published between 2013 and January 2023 (Fig. 1), approximately 40% of which were published between 2020 and 2023. More than 20% of the retrieved studies were conducted in Iran and USA.

**Table 2:** Study characteristics

Information		No/%	Ref.
**Publication Year**	2020–2023	*N* = 40, 43%	[15–17, 29–65]
	2016–2019	*N* = 26, 28%	[14, 18, 66–89]
	2013–2015	*N* = 25, 27%	[90–114]
		*N* = 91	
**Number of Article Per Country**	Iran	*N* = 11, 12%	[47, 51, 68, 70, 73, 75, 78, 90, 99, 100, 107]
	USA	*N* = 8, 9%	[33, 34, 74, 85, 97, 103, 109, 111]
	Italy, China	*N* = 5, 5%	[36, 44, 52, 53, 71, 82, 84, 86, 87, 96]
	Canada, Brazil	*N* = 4, 4%	[18, 38, 40, 64, 93, 95, 105, 108]
	India, South Korea, Taiwan	*N* = 3, 3%	[17, 32, 37, 43, 45, 48, 54, 67, 92]
	Bangladesh, Saudi Arabia, Ethiopia, Spain, Portugal, Slovakia, Croatia, Australia	*N* = 2, 2%	[42, 46, 50, 55, 58, 60, 63, 69, 76, 77, 89, 98, 102, 104, 112, 114]
	Lebanon, Turkey, Nigeria, Cambodia, Netherlands, Latvia, Tanzania, Sudan, New Zealand, Germany, Colombia, Indonesia, Scandinavia, Nordic countries, Poland, Lithuania, Malaysia, Serbia, Palestine, Czech Republic, Greece, Marco	*N* = 1, 1%	[14, 15, 31, 35, 39, 41, 49, 57, 59, 61, 62, 65, 66, 72, 80, 81, 83, 88, 91, 94, 101, 110]
	Other (Reviews)	*N* = 7, 8%	[16, 29, 30, 56, 79, 106, 113]
		*N* = 91	
**Study Sample**	Hospital (*n* = 13,221)	*N* = 79, 87%	[14, 15, 32–41, 18, 43–48, 50–53, 49, 54, 55, 58, 59, 76, 83–87, 60, 88, 66–72, 89, 73, 61, 74, 77–82, 90, 91, 102, 62, 108–112, 114, 92–95, 63, 96–101, 103–105, 64, 65]
	Experts(*n* = 173)	*N* = 6, 6%	[16, 17, 29, 57, 75, 107]
	Papers (*n* = 143)	*N* = 4, 4%	[30, 69, 106, 113]
	Patients (*n* = 100)	*N* = 1, 1%	[31]
	Laboratory test (*n* = 422)	*N* = 1, 1%	[42]
		*N* = 91	
**Indicators Calculation**	Quantitative (with calculations)	*N* = 85, 93%	[14, 15, 65, 30–36, 38, 29, 16, 39–48, 17, 50–59, 49, 76, 66, 83–89, 67, 60, 68–75, 77, 78, 61, 79–82, 90, 91, 102, 108–110, 62, 111–114, 93–97, 99, 63, 100, 101, 103–105, 64]
	Qualitative( without calculations)	*N* = 6, 6%	[18, 37, 92, 98, 106, 107]
		*N* = 91	
**Unit of Evaluation**	Hospital Level	*N* = 24, 26%	[14, 17, 73, 75, 77–79, 81, 82, 114, 94, 96, 34, 99, 100, 104, 105, 41, 46, 51, 54, 86, 66, 71]
	Departmental Level		[15, 16, 30–33, 35, 29, 36–39, 18, 40, 42–45, 47, 48, 50, 52, 53, 49, 55–59, 76, 83–85, 87, 60, 88, 89, 67–70, 72, 74, 80, 90, 61, 91, 102, 108–113, 92, 93, 62, 95, 97, 98, 101, 103, 106, 107, 63–65]
	Administrative Departments	*N* = 29, 32%	[15, 61, 48, 52, 53, 55, 83–85, 87, 67, 68, 30, 80, 90, 109, 113, 93, 97, 101, 106, 107, 32, 33, 35, 36, 29, 44, 45]
	Hospital Clinical Wards	*N* = 30, 33%	[16, 18, 47, 50, 56–59, 88, 89, 69, 70, 62, 72, 74, 91, 102, 108, 111, 112, 92, 95, 103, 63–65, 31, 38–40]
	Para-Medical Departments	*N* = 8, 9%	[37, 42, 43, 49, 60, 76, 98, 110]
		*N* = 91	

### Study characteristic

As shown in Table 2, in 85% of the reviewed studies, a number of hospitals (1 to 3828 hospitals, 13,221 hospitals in total) were evaluated. More than 90% of the studies used a quantitative approach. In more than 70% of the studies, hospital evaluation occurred at the department level, which can also be divided into three levels: administrative, clinical ward, and paramedical department. In addition, the administrative departments consist of 13 departments, including financial management [48, 55, 61, 67, 68, 80, 83, 109, 113], supply chain management and warehouse [15, 43, 84], value-based purchasing [33, 85], human resource management [97, 101], medical equipment [32, 87], health information management department [90], information systems [106], nutritional assessment [93], energy management [30, 45, 92], facility management [52, 53], building sustainability and resilience [35], research activities [44], and education [107].

The clinical wards consisted of 8 wards, namely, emergency departments (EDs) [16, 39, 56, 57, 69, 70, 89], surgery departments [58, 62, 63, 91, 102], intensive care units (ICUs) [47, 64, 65], operating rooms (ORs) [38, 88, 108], surgical intensive care units (SICUs) [111], obstetrics and gynecology department [59], neonatal intensive care units (NICUs) [74, 103] and quality of care [18, 31, 40, 50, 72, 92, 95, 112] indicators. The paramedical departments consisted of 3 departments, pharmacy [60, 76, 98], laboratory and blood bank [37, 42, 43, 49], and outpatient assessment [86] indicators.

**Table 3:** Performance dimensions and related indicators

Performance dimensions	Category	Sub-category	Indicators	Ref.
**Productivity** [14, 18, 29, 34, 38, 39, 41, 46, 47, 50, 51, 54–56, 58–61, 63, 66, 69, 71–73, 75–79, 81–83, 86, 88, 89, 91–94, 96, 97, 99–102, 104–106, 108, 110, 111, 113, 114]	Organizational Management	Bed Utilization Rate	Bed occupancy rate	[14, 61, 83, 75, 77–79, 82, 91, 92, 99, 100, 63, 105, 38, 46, 47, 51, 55, 59, 76]
			Bed turnover ratio	[47, 51, 100, 105, 59, 86, 73, 75, 77, 78, 96, 99]
			Length of stay	[14, 18, 51, 54, 55, 59, 76, 86, 89, 66, 69, 71, 61, 72, 73, 75, 77–79, 82, 91, 102, 108, 34, 110, 111, 113, 92, 93, 96, 97, 99–101, 38, 104–106, 29, 39, 41, 47, 50]
		Hospital Characteristics	Case-mix index	[94, 111, 113]
			Number of operations/ procedures	[54, 79, 81]
		Quality Improvement	Surgery delay	[18, 38, 63, 88]
			Cancellation of surgery	[38, 58, 63, 75, 88]
			Lab cancellation rate	[106]
	Clinical Management	Service Delivery and Treatment	Hospitalization rate	[16, 58, 69, 73, 76, 81, 89, 94, 97, 111, 113, 114]
			Inpatient visits	[59, 78, 113]
			Emergency department (ED) visits	[56, 78, 89, 92, 113]
			Outpatient visits	[55, 60, 76, 78, 104, 113, 114]
**Efficiency** [14, 15, 38, 29, 39, 41, 45–47, 50–52, 16, 53–55, 57–59, 76, 84–86, 61, 87, 89, 66, 71, 73–75, 78, 81, 90, 63, 91, 109, 111, 113, 114, 92, 95–97, 99, 64, 103–106, 32, 33, 35, 36]	Organizational Management	Resource Management	ED bed numbers	[36, 41, 46, 48, 54, 59, 76, 81, 91, 104, 109, 113, 114]
			Bed number by types	[16, 33, 61, 73, 89, 96, 99, 105]
			Bed-staff ratios	[33, 76, 86, 92, 96, 105]
			Bed-physician ratios	[76, 96]
			Bed-patients ratios	[66, 89]
			Staff-patients ratios	[16, 47, 55, 89, 95]
			Physician-patients ratios	[16, 55, 96]
			Exams-physicians ratios	[106]
		Human Resource Management	Medical staff numbers	[16, 33, 39, 41, 46, 64, 73, 76, 86, 89, 91, 95, 96, 99, 113, 114]
			Non-medical staff numbers	[16, 33, 36, 41, 81, 86, 91, 95, 99, 113, 114]
			Physicians number	[54, 57, 76, 81, 92, 99, 104, 114]
			Physicians-nurses ratio	[86, 95, 96]
		Paramedical Assessment	Number of laboratory tests	[97, 106, 113]
			Number of imagines	[46, 97]
		Medical Management	Number of surgeries	[78]
			Number of coded diagnoses	[111]
			Number of deliveries	[41, 74]
			Number of cesarean birth	[71, 74, 76, 103]
			Number of medical records	[90]
	Administrative Management	Supportive Units Assessment	Facilities management	[45, 52, 53, 75]
			Supply chain management	[14, 15, 29, 33, 35, 84, 85]
			Medical equipment measures	[32, 46, 54, 87, 106]
			Number of discharge	[33, 41, 50, 61, 89, 95, 99, 105]
**Effectiveness** [14, 18, 50, 55, 56, 58, 85, 89, 70–72, 74, 63, 75, 77–79, 82, 91, 110, 111, 92, 93, 64, 94–97, 101, 103, 105, 65, 33, 34, 38, 40, 47]	Organizational Management	Patient Safety	Mortality rate	[18, 63, 85, 70–72, 74, 75, 77–79, 82, 65, 91, 110, 111, 92, 94, 96, 101, 103, 105, 33, 34, 38, 40, 47, 50, 56]
			Readmission rate (24–72 h RR)	[14, 18, 58, 89, 71, 79, 82, 110, 111, 92–94, 64, 95, 97, 101, 33, 34, 40, 47, 50, 55, 56]
**Speed** [14, 16, 42, 43, 50, 54, 55, 57, 87–89, 69, 18, 70–72, 75, 79, 82, 90, 102, 108, 92, 49, 106, 63, 32, 34, 37–39]	Organizational Management	Time Management	Waiting time	[14, 18, 55, 69, 75, 79, 82, 89, 90, 106]
			Interval between discharge process	[16, 89, 90]
			Laboratory sample or report intervals	[106]
			Lab turnaround time (TAT)	[37, 42, 43, 49, 106]
			Imaging exam/result intervals	[50, 80]
			Time of surgery	[38, 45, 55, 63, 88, 102, 108]
			ED requests turnaround time	[16, 34, 39, 54, 57, 70, 71, 89]
			Medical information request time	[90]
			Medical equipment repairing time	[32, 87]
**Development** [14, 17, 57, 60, 83, 86, 90, 96, 101, 107, 113]	Organizational Management	Training and Education Management	Staff training number,	[14, 86, 96, 107]
			Hours of training	[14, 57, 83, 107]
			Per capita teaching hours	[101, 107]
			Training courses	[17, 57, 60, 86, 90, 96, 101, 107]
			Student training	[57, 60, 96, 113]
**Safety** [14, 16, 29, 39, 40, 43, 47, 50, 55–57, 85, 18, 71, 72, 74, 75, 77, 79, 82, 90, 102, 111, 60, 92–96, 101, 103, 106, 62–65, 34, 38]	Clinical Management	Patient Safety	Hospital infection rate	[14, 34, 40, 75, 77, 92]
			Surgical site infection	[18, 38, 63]
			ICU infection rate	[47, 64, 74, 103, 111]
			Nosocomial infection	[57, 79, 82, 96]
			Other infections	[18, 43, 55, 65, 96]
			Accidents or adverse Events	[38, 43, 56, 60, 63, 64, 79, 82, 92, 94–96]
			Incidents or Errors rates	[14, 16, 47, 57, 60, 64, 75, 79, 82, 92, 106]
			Complications	[34, 38, 40, 47, 50, 55, 57, 71, 72, 85, 92, 94, 101]
			Surgical safety checklist	[18, 62, 63]
			Surgical safety measures	[55, 62, 63, 71, 102]
			ED safety measures	[16, 39, 56, 57]
			ICU safety measures	[47, 64, 65, 92, 111]
			Safety standards in the archives	[90]
			Nutrition risk screening	[93]
			Other safety measures	[18, 29, 85, 92]
**Quality of work life** [14, 16, 46, 53, 75, 79, 82, 83, 86, 88, 92, 95, 106]	Organizational Management	Human Recourse Management	Staff turnover (staff leaving transferring etc.)	[14, 75, 79, 82]
			Staff workload	[16, 86, 106]
			Staff work condition (absenteeism, sickness leave, …)	[75, 79, 86, 92, 95],
			Staff/physicians working hours	[46, 53, 83, 88]
**Quality** [39, 43, 63, 70, 71, 85, 87, 89, 90, 95, 106, 110]	Organizational Management	Quality Improvement	ED Left against medical advice	[39, 70, 71, 89]
			Lab Quality Control (QC) failures	[43, 63, 106]
			Imaging QC failures	[106]
			Error in Medical report	[90]
			Medical equipment QC failure	[87, 90]
	Clinical Management	Patient Safety	Compliance rate	[71, 106, 110]
			Pain management	[85, 95]
**Satisfaction** [14, 16, 29, 31, 33, 34, 63, 75, 79, 82, 83, 86, 90, 92, 96, 98, 101, 106, 112]	Organizational Management	Human Recourse Management	Staff satisfaction rate	[14, 16, 29, 75, 79, 82, 106]
	Clinical Management	Service Recipients Rights	Patient satisfaction rate	[14, 29, 31, 33, 34, 63, 75, 79, 82, 86, 90, 92, 96, 98, 101, 112]
			Rate of complaints	[14, 75, 79, 82, 83, 90]
**Innovation** [14, 44, 57, 107, 113]	Organizational Management	Research Assessment	Number of publications	[14, 44, 107, 113]
			Average impact factor	[44]
			Participation in scientific events	[57, 107]
**Appropriateness** [15, 16, 114, 93, 96–98, 103, 60, 50, 56, 76, 86, 71, 74, 78]	Clinical Management	Service Delivery and Treatment	Treatment rate	[16, 56, 71, 86, 96, 114]
			NICU treatment measures	[74, 103]
			Drug and medication measures	[15, 60, 50, 96–98]
			Obstetrics and Gynecology measures	[74, 76, 78, 103]
	Administrative Management	Supportive Units Assessment	Nutritional measures	[93]
**Evaluation** [16, 48, 60, 66, 85, 89, 90, 92, 95, 96, 98, 105]	Clinical Management	Service Delivery and Treatment	ED patients acuity levels	[16, 89]
		Paramedical Assessment	Lab scoring	[96]
			Imaging inspection	[96]
			Medication use evaluations (MUEs)	[60, 98]
	Administrative Management	Supportive Units Assessment	Medical information measures	[90, 92, 95, 96]
	Organizational Management	Hospital Characteristics	Hospital Rate	[48, 66, 85, 105]
**Profitability** [14, 16, 80, 82, 109, 113, 95, 96, 17, 54, 55, 86, 66–68, 75]	Administrative Management	Financial Management	Profit	[14, 54, 66–68, 75, 82, 109],
			Margin ratio	[55, 95, 109]
			Assets ratios	[54, 55, 68, 86, 96]
			Revenue or income	[14, 16, 17, 54, 68, 80, 86, 96, 113]
**Cost** [14, 15, 54, 55, 57, 83, 86–88, 66, 68, 71, 16, 75, 80–82, 90, 109, 113, 114, 94, 95, 18, 96, 63, 34, 41, 48, 52, 53]	Administrative Management	Financial Management	Budget allocation	[14, 15, 52, 53, 57]
			Personnel costs	[14, 55, 86–88, 75, 80, 113]
			Non-personnel costs	[14, 16, 86–88, 68, 75, 90, 109, 113, 114, 95, 63, 96, 34, 41, 48, 52–55]
			Average costs	[18, 55, 66, 71, 75, 81–83, 86, 88, 94, 96, 113, 114]
**Economy** [14, 17, 33, 54, 55, 67, 68, 75, 79, 80, 86, 96, 104, 105, 109, 113]	Administrative Management	Financial Management	Financial ratios	[14, 55, 67, 68, 86, 96]
			Financial Indices	[14, 17, 33, 54, 55, 67, 68, 75, 79, 80, 86, 104, 105, 109, 113]
**Coherence** [85]	Administrative Management	Financial Management	Discharge process	[85]
**Patient centeredness** [17, 18, 33, 85, 95]	Clinical Management	Service Recipients Rights	Patient experience	[17, 18, 33, 85, 95]
**Equity** [33, 34, 48, 55, 56, 76, 104, 110]	Clinical Management	Service Recipients Rights	Health equity	[33, 34, 48, 55, 56, 76, 104, 110]
**Relationship** [41, 60, 62]	Clinical Management	Service Delivery and Treatment	Medical consultations and discussions	[41, 60, 62]
**Sustainability** [30, 35, 36, 45, 52, 53]	Administrative Management	Supportive Units Assessment	Energy management	[30, 35, 36, 45, 52, 53]
**Flexibility** [14]	Administrative Management	Supportive Units Assessment	Emergency response rate	[14]

With regard to data categorization, firstly, a total of 1204 indicators in 91 studies were extracted and after detailed examination, 43 indices (such as hospital ownership, level of care, admission process, and personal discipline) were removed due to their generality and impossibility of calculation in the hospital environment. Then, 1161 performance indicators were entered in this research and were categorized based on the performance criteria (more details about the indicators can be found in Appendix 1). Secondly, 145 functional dimensions, including divisions based on different departments and units of the hospital, were defined according to several focus group discussions with 5 health experts. Then, re-categorization and functional summarization were performed, after which 21 performance dimensions were finalized.

As shown in Table 4, the 21 performance dimensions were divided into three parts: category, subcategory, and related indicators. Additionally, according to the hospital levels, there were three categories: ‘organizational management’, ‘clinical management’, and ‘administrative management’. Then, according to the type of indicators, fifteen subcategories were defined for the 110 selected main indicators.

### Performance dimensions

The ‘productivity’ dimension focuses on indicators reflecting the macro-performance of the hospital, considering that this index is more effective and efficient. The ‘efficiency’ dimension focuses on general performance indicators for the optimal use of resources to create optimal output in the hospital. The ‘effectiveness’ dimension is a general performance indicator with an outcome view. The ‘speed’ dimension focuses on the indicators that show attention to the service delivery time and the speed of the procedures. The ‘development’ dimension focuses on matters related to employees’ and students’ training and related training courses. In terms of ‘safety’ dimension, there were issues related to patient safety, unwanted and harmful events, and hospital infections.

The “quality of work life” dimension emphasizes matters related to personnel volume and work conditions. The ‘quality’ dimension is related to the quality of service provided in different parts of the hospital and possible complications in improving the quality of services. The ‘satisfaction’ dimension focuses on the satisfaction of patients, employees, and their complaints. The ‘innovation’ dimension relates to the research process and its output. The ‘appropriateness’ dimension involves proper service from clinical departments, pharmaceutical services, and patient treatment. The ‘evaluation’ dimension focuses on the indicators related to the assessment scores of the para-clinical departments of the hospital.

The ‘profitability’ dimension focuses on the overall output indicators for income and profitability. The ‘cost’ dimension focuses on indicators related to general expenditures and the average cost per bed and patient and budgeting. The ‘economy’ dimension is related to financial rates and their indicators. The ‘coherence’ dimension emphasizes the indicators related to the continuity of the service delivery process. The ‘patient-centeredness’ dimension focuses on the indicators related to the patient’s experience of the facility, environment, treatment processes, communications, and relevant support for the patient. The ‘equity’ dimension studies indicators related to social and financial justice and life expectancy. The ‘relationship’ dimension evaluates the process of consultations and discussions required during the patients’ care provided by the treatment team. The ‘sustainability’ dimension focuses on indicators related to energy standards. The ‘flexibility’ dimension focuses on the hospital’s response to the crisis.

According to Table 4, most studies focused on ‘efficiency’, ‘productivity’, ‘safety’ and ‘effectiveness’ as performance dimensions in 54, 53, 38 and 37 studies, respectively (40–70% of studies). In the ‘efficiency’ subcategory, resource management, supportive unit assessment, and human resource management indicators were the first to third most common indicators used in 26, 23 and 22 studies, respectively (approximately 25% of the studies).

In addition, for the ‘efficiency’ dimension, ‘medical staff numbers’, ‘emergency department bed numbers’, and ‘nonmedical staff numbers’ were reported in 16, 13, and 11 studies, respectively (between 20 and 30% of the studies). For the ‘productivity’ subcategory, ‘bed utilization rate’ and ‘service delivery and treatment’ were reported in 50% and 20% of the studies, respectively (46 and 19 out of 91).

Additionally, for the ‘productivity’ dimension, the ‘length of stay’ indicator was used more than others and reported in approximately 80% of the studies (43 out of 53), followed by the ‘bed occupancy rate’ in approximately 40% of the studies (21 out of 53). The ‘bed turnover ratio’ and ‘hospitalization rate’ were also reported in 12 studies. Furthermore, for ‘safety’ dimensions, all indicators were in the ‘patient safety’ subcategory, which has been reported in 38 studies, and ‘complications’, ‘accidents or adverse events’, and ‘incidents or errors rates’ were the most concentrated indicators by researchers in 13, 12, and 11 studies, respectively. The performance dimension of ‘effectiveness’ was presented in 37 studies (40%), with only two indicators, ‘mortality rate’ in 29 studies and ‘readmission rate’ in 23 studies.

### Performance categories

Considering the three categories shown in Table 4, ‘organizational management’ indicators were more commonly used among the other two categories (‘clinical’ and ‘administrative’) and were present in more than 85% of the studies (78 out of 91). Two categories, ‘clinical management’ and ‘administrative management’, were reported in 62 and 51 studies, respectively.

### Performance subcategories

Considering the 14 subcategories shown in Table 4, both the ‘bed utilization rate’ and ‘patient safety’ indicators were mentioned in 46 studies and were more common among the other subcategories. The second most common indicator of the ‘financial management’ subcategory was reported in 38 studies. At the third level, both the ‘human resource management’ and ‘time management’ indicators were presented in 31 studies. The ‘paramedical’ subcategory indicators were presented in less than 10% of the studies [60, 96–98, 106, 113].

### Performance indicators

According to the indicator columns in Table 3, the most used indicators in reviewed studies were the length of stay, mortality rate, and readmission rate in 47%, 32%, and 25% of studies, respectively. Bed occupancy rate and non-personnel costs were reported in 23% of studies. Additionally, among the 110 indicators, 16 indicators, namely, the lab cancellation rate, exam-physician ratios, number of coded diagnoses, number of medical records, laboratory sample/report intervals, medical information request time, safety standards in the archives, nutritional risk screening, imaging quality control failures, errors in medical reports, average impact factor, nutritional measures, laboratory scoring, imaging inspection, discharge process and emergency response rate, were reported in less than 1% of the studies.

The classification of the indicators in Table 4 was performed based on the chain model, which included the input, process, output, outcome and impact. The assignment of the indicators to each category was performed according to the experts’ opinions. For instance, the number of publications by academic member of an academic hospital and the average impact factor of those publications were considered outcome indicators. As depicted in the Table 4, most studies (80%) focused more on output indicators. Additionally, fifteen studies focused on introducing and extracting some of the input, process, output, outcome and impact indicators; among those, only one study [96] has examined the input, process, output and impact indicators simultaneously.

**Table 4:** Indicators by types

		No. of Ref
**Input**	ED Bed numbers, Bed number by types, Bed-staff ratios, Bed-physician ratios, Hospitalization rate, Medical staff numbers, Non-medical staff numbers, Physicians number, Physicians-nurse’s ratio, Facilities management, Medical equipment measures	*N* = 39 [16, 30, 31, 33, 38, 39, 43, 44, 46, 50, 51, 53, 54, 56, 60, 61, 68, 76, 76, 78, 84, 85, 89, 92, 94, 97, 98, 100–104, 107, 109, 110, 113, 118–120]
**Process**	Supply chain management, Number of Discharge, Waiting time, Interval between discharge process, Laboratory sample/report intervals, Lab turnaround time, Imaging exam/result intervals, Time of surgery, ED requests turnaround time, Medical information request time, Medical equipment repairing time, Surgical safety measure, Staff training number, Hours of training, Per capita teaching hours, Training courses, Student training, Accidents/adverse events, Incidents/errors rate, Complications, Surgical safety checklist, ED safety measures, ICU safety measures, Safety standards in the archives, Nutrition risk screening, Other safety measures, Staff turnover (staff leaving, transfer,…), Staff workload, Staff work condition (absenteeism, sickness leave, …), Staff/physicians working hours, Compliance rate, Pain management, Nutritional measures, ED Patients Acuity levels, MUEs, Discharge process, Patient experience, Medical consultations and discussions, Emergency response rate	*N* = 62 [14–18, 49, 60–63, 30–32, 34–41, 44, 45, 47, 51–53, 55–59, 76, 83–85, 89, 66, 67, 69, 75, 77, 78, 81, 82, 90, 91, 102, 111, 114, 92, 94, 95, 98–101, 103, 105, 106, 120, 121]
**Output**	Bed occupancy rate, Bed turnover ratio, Length of stay, Case-mix index, Number of operations/ procedures, Inpatient visits (IVs), Emergency room visits, Outpatient visits, Bed-patients ratios, Staff-patients ratios, Physician-patients ratios, Exams-physicians ratios, Number of laboratory tests, Number of imagines, Number of surgeries, Number of coded diagnoses, Number of deliveries, Number of cesarean birth, Number of medical records, Participation in scientific events, NICU treatment measures, Drug and medication measures, Obstetrics and gynecology measures, Lab scoring, Imaging inspection, Profit, Margin ratio, Assets ratios, Revenue or income, Budget allocation, Personnel costs, Non-personnel costs, Average costs, Financial ratios, Financial indices, Energy management	*N* = 76 [14–18, 24, 61, 62, 64, 31–33, 35–39, 43–48, 50–57, 59, 76, 84–89, 67–75, 77–81, 90, 91, 102, 109–114, 93–101, 103–107]
**Outcome**	Surgery delay, Cancellation of surgery, Lab cancellation rate, Mortality rate, Readmission rate, Hospital infection rate, Surgical site infection, ICU infection rate, Nosocomial infection, Other infections, ED left against medical advice, Lab QC failures, Imaging QC failures, Error in medical report, Medical equipment QC failure, Staff satisfaction rate, Patient Satisfaction rate, Rate of complaints, Number of publications, Average impact factor, Treatment rate, Medical information measures, Hospital rate, Health equity	*N* = 58 [14, 16, 18, 60, 62, 63, 65, 31, 35–38, 29, 41, 42, 45–47, 52–54, 57, 76, 83–86, 66, 67, 69–72, 75, 77–81, 90, 91, 102, 108–114, 94–98, 100, 101, 103, 104]
**Impact**	-	*N* = 0

Additionally, in approximately 42% (36 out of 91) of the studies, the indicators’ definitions, formulas, or descriptions have been illustrated, while less than 10% of the studies have defined measuring units, standard or benchmark units for all studied indicators [15, 43, 45, 51, 52, 57, 67].

Overall, nine studies related to hospital performance evaluation were conducted using systematic review methodologies (five systematic reviews [16, 29, 30, 56, 113], two literature reviews [79, 80], one narrative review [98] and one brief review [92]). Most of these studies focused on extracting performance indicators from one or more hospital departments (e.g., the emergency department) [16, 56], hospital laboratory and radiology information systems [106], supply chain performance [29], resources and financial results and activity [113], hospital water consumption [30], and the pharmaceutical sector [98]. Other reviews included a three-step process to review, evaluate and rank these hospital indicators in a systematic approach [16], or to evaluate performance indicator models to create an interactive network and visualize the causal relationships between performance indicators [79]; moreover, some have focused on the importance of indicators to ensure adequate coverage of the relevant areas of health care services to be evaluated [92].

Only one scoping review aimed to identify current assessments of hospital performance and compared quality measures from each method in the context of the six qualitative domains of STEEEP (safety, timeliness, effectiveness, efficiency, equity, and patient-centeredness) of the Institute of Medicine (IOM) in accordance with Donabedian’s framework and formulating policy recommendations [115].

In addition, 21 studies divided performance indicators into 2 to 6 dimensions of performance. Also, the reviewed studies included 2–40 indicators in zero [29, 30, 98] to 6 domains [34]. Moreover, none of the studies have tried to comprehensively summarize and categorize the performance indicators in several categories, focusing on all the indicators reflecting the performance of the entire hospital organization, or the indicators of administrative units or clinical departments.

## Discussion

In this scoping review, a unique set of hospital performance evaluation indicators related to the various performance dimensions was categorized from 91 studies over the past ten years.

Similarly, in a study, 19 performance dimensions, 32 sub-dimensions, and 138 indicators were extracted from only six studies. Those dimensions were described by all studies included in the review, but only three studies specified the relevant indicators, and the list provided for all possible indicators was not comprehensive. Also, despite current review, there was no classification of indicators based on the hospital levels: managerial, clinical, or organizational levels [116]. Another study has similarly investigated the performance evaluation indicators of the hospital in such a way that among 42 studies, 111 indicators were presented in the four categories: input, output, outcome, and impact. But, there was no classification of indicators based on performance dimensions and hospital levels [117].

In this study, the importance of categorized indicators, for the first time to our knowledge, was determined based on their frequency of use in the published literature (Appendix 2). The ‘Organizational management’ indicators were the most common compared with the other two categories (‘clinical’ and ‘administrative’). It could be because of the fact that the indicators such as ‘bed occupancy rate’, ‘average length of stay’, ‘mortality rate’, ‘hospital infection rate’, and ‘patient safety’ are easier to be registered in hospital software compared to other indicators, and also they better reflect the overall performance of hospital. Thus, researchers are more interested in using these indicators.

Considering 14 subcategories, indicators related to three subcategories i.e. bed utilization, patient safety and financial management are the most frequent used indicators for hospital performance evaluation. It reflects the need of hospital managers to increase the profitability of hospital in one hand, and to control cost on the other hand. As a results, researchers have paid special attention to ‘cost income’, ‘profitability’, ‘economic’, etc., as indicators for evaluating hospital performance.

When considering indicators by type, more studies have focused on output indicators, while input indicators were the least common used. This might be because of the fact that at hospital level, it is difficult for managers to change those inputs such as ‘beds’, ‘human resources’, ‘equipment and facilities’. In addition, due to the complexity of interdepartmental relationships in hospitals, process indicators seemed to provide more variety for analysis than input indicators, so they were more often used. As mentioned above, output indicators were the most used indicators for hospital performance evaluation due to their ease of calculation and interpretation.

The main purpose of this paper was to identify a comprehensive set of indicators that can be used to evaluate hospital performance in various hospital settings by being distilled into a smaller and more related set of indicators for every hospital or department setting. future studies could be designed to validate each set of indicators in any specific context. In addition, they could investigate the relationship between the indicators and their outcomes of interest and the performance dimension each could address. This will enable hospital managers to build their own set of indicators for performance evaluation both at organization or at department level. Also it should be mentioned that.

Although some previous studies have provided definitions for each indicator and determined the standard criteria for them, this was not done in this study because the focus of this study was to provide a collection of all the indicators used in hospital performance evaluation, which resulted in the identification of more than a thousand indicators without limiting to specific country or context. So while preparing a smaller set of indicators, specific conditions of each country, such as the type of health system and its policy, the type of financing system, and the structure of services, should be taken into account to select appropriate indicators.

In addition, although it is important to examine the scope of each article to compare the list of indicators and the relationships between the dimensions of the hospital in terms of size and type and between the number and type of selected indicators, this was considered beyond the scope of this review due to the high number of indicators, which made the abovementioned investigations impossible. Future studies could do that while working with a smaller set of indicators.

## Conclusion

This review aimed to categorize and present a comprehensive set of indicators for evaluating overall hospital performance in a systematic way. 1161 hospital performance indicators were drawn from 91 studies over the past ten years. They then were summarized into 110 main indicators, and categorized into three categories: 14 subcategories, and 21 performance dimensions This scoping review also highlighted the most frequent used indicators in performance evaluation studies which could reflect their importance for that purpose. The results of this review help hospital managers to build their own set of indicators for performance evaluation both at organization or at department level with regard to various performance dimensions.

As the results of this review was not limited to any specific country or context, specific conditions of each country, such as the type of health system and its policy, the type of financing system, and the structure of services, should be taken into account while selecting appropriate indicators as a smaller set of indicators for hospital performance evaluation in specific context.

### Supplementary Information

**Supplementary Material 1.**

**Supplementary Material 2.**

**Supplementary Material 3.**

## Data Availability

The datasets used and/or analyzed during the current study are available from the corresponding author on reasonable request.

## Abbreviations

GDP: Gross domestic product
PRISMA-ScR: Preferred Reporting Items for Systematic reviews and Meta-Analyses extension for Scoping Reviews
ED: Emergency departments
ICU: Intensive care unit
OR: Operating room
SICU: Surgical intensive care unit
NICU: Neonatal intensive care unit
RR: Readmission rate
QC: Quality Control
MUE: Medication use evaluation
STEEEP: safety, timeliness, effectiveness, efficiency, equity, and patient-centeredness
IOM: Institute of Medicine
