# Supplementary material for: Hospital performance evaluation indicators: a scoping review
Source: BMC Health Serv Res. 2024 May 1;24:561. doi: 10.1186/s12913-024-10940-1 (PMC11064245; doi:10.1186/s12913-024-10940-1)
Supplement: Supplementary file 1 — Supplementary Material 1. [file 12913_2024_10940_MOESM1_ESM.docx]

| **Appendix 1: Hospital performance indicators’ details** | | | | | | | | | | | |
| --- | --- | --- | --- | --- | --- | --- | --- | --- | --- | --- | --- |
| **Row** | **Performance aspects** | **Category** | **Sub-category** | **Indicators** | **Sub-indicators** | **Input** | **Process** | **Output** | **Outcome** | **Effect** | **Ref** |
| 1 | **Productivity** | **Organizational Management** | **Bed Utilization Rate** | **Bed Occupancy rate** | Bed occupancy rate= represents the degree to which available beds are used, thus, very low values are generally associated with lower efficiency levels. However, high rates may indicate a high prevalence of comorbidities, low resolvability, low emergency resource reserve or an imbalance between supply and demand. Furthermore, as indicators regarding bed utilization are closely related to each other, bed occupancy rate can be influenced by mean length of stay and the bed turnover index |  |  | * |  |  | (1) |
| 2 |  |  |  |  | Bed-occupation rate for inpatient service |  |  | * |  |  | (2) |
| 3 |  |  |  |  | Bed occupancy ratio= Proportion of beds occupied over a specific year |  |  | * |  |  | (3) |
| 4 |  |  |  |  | BOR, bed occupancy rate |  |  | * |  |  | (4) |
| 5 |  |  |  |  | Bed occupancy=Bed occupancy of the hospital, such as average daily census/beds in service, percentage of bed emptiness |  |  | * |  |  | (5) |
| 6 |  |  |  |  | Bed occupancy rate (BOR) |  |  | * |  |  | (6) |
| 7 |  |  |  |  | Bed Occupancy Rate (BOR)=Percentage of functional beds in a hospital occupied by the inpatients for a given time period. It is a measure of financial performance |  |  | * |  |  | (7) |
| 8 |  |  |  |  | Bed occupancy |  |  | * |  |  | (8) |
| 9 |  |  |  |  | The bed occupancy rate |  |  | * |  |  | (9) |
| 10 |  |  |  |  | BOR = Number of days occupied − number of beds/active day − number of active beds |  |  | * |  |  | (10) |
| 11 |  |  |  |  | Bed occupancy rate (BOR) |  |  | * |  |  | (11) |
| 12 |  |  |  |  | Bed occupancy rate |  |  | * |  |  | (12) |
| 13 |  |  |  |  | Bed occupancy ratio=Average percentage occupancy of hospital beds |  |  | * |  |  | (13) |
| 14 |  |  |  |  | Occupied-bed-days or total inpatient-days= Refers to the sum of total number of days all admitted patients spent in the hospital for a given year |  |  | * |  |  | (14) |
| **Row** | **Performance aspects** | **Category** | **Sub-category** | **Indicators** | **Sub-indicators** | **Input** | **Process** | **Output** | **Outcome** | **Effect** | **Ref** |
| 15 |  |  |  |  | Occupied bed‑days (OBD) |  |  | * |  |  | (6) |
| 16 | **Productivity** | **Organizational Management** | **Bed Utilization Rate** | **Bed Occupancy rate** | Bed Occupancy Rate |  |  | * |  |  | (15) |
| 17 |  |  |  |  | Bed occupancy Ratio= Average percentage occupancy of hospital beds |  |  | * |  |  | (16) |
| 18 |  |  |  |  | The inpatient bed occupancy rate =shows the relationship between the number of hospitalization days and the number of establishment beds. It is an access relevant measure as it is closely related to the waiting time and beds’ availability. Studies show that the ideal value for this indicator is around 85%. Values above generally represent a beds’ shortage in the hospital. It is a variable that should be maximized up to 85% and reduced after that. Because of BoD specifications, this variable should only increase; thus, we transformed values higher than 85%, subtracting the excess percentage above 85 from this value. |  |  | * |  |  | (17) |
| 19 |  |  |  |  | Number of hours on occupied beds |  |  | * |  |  | (18) |
| 20 |  |  |  |  | Average bed occupancy rate |  |  | * |  |  | (19) |
| 21 |  |  |  |  | Surgical Bed Occupancy Rate |  |  | * |  |  | 9 |
| 22 |  |  |  |  | OR occupancy rate |  |  | * |  |  | (20) |
| 23 |  |  |  |  | OR occupancy rate in relation to the anesthesiologists’ workload |  |  | * |  |  | (20) |
| 24 |  |  |  | **Bed Turnover Ratio** | Bed Turnover Ratio |  |  | * |  |  | (4) |
| 25 |  |  |  |  | Bed Turnover Rate |  |  | * |  |  | (15) |
| 26 |  |  |  |  | Bed turnover index= represents the extent to which the capacity of which is being used, expressed by the number of hospitalizations per bed in a given period. Although this may be considered one of the main indicators for productivity and efficiency, high values may indicate re-hospitalizations, unnecessary hospitalizations or early discharges. |  |  | * |  |  | (1) |
| 27 |  |  |  |  | Bed Turnover Rate (BTR)=The number of times each hospitals bed |  |  | * |  |  | (7) |
| **Row** | **Performance aspects** | **Category** | **Sub-category** | **Indicators** | **Sub-indicators** | **Input** | **Process** | **Output** | **Outcome** | **Effect** | **Ref** |
| 28 | **Productivity** | **Organizational Management** | **Bed Utilization Rate** | **Bed Turnover Ratio** | Turnover rate of hospital beds |  |  | * |  |  | (21) |
| 29 |  |  |  |  | BTR = Total number of admissions/average number of active beds |  |  | * |  |  | (10) |
| 30 |  |  |  |  | Medical institution bed turnover ratio, ≥19 times per year |  |  | * |  |  | (22) |
| 31 |  |  |  |  | Bed turnover rate (BTR) |  |  | * |  |  | (11) |
| 32 |  |  |  |  | Bed turnover rate |  |  | * |  |  | (8) |
| 33 |  |  |  |  | Bed turnover ratio (BTR) |  |  | * |  |  | (6) |
| 34 |  |  |  |  | Bed turnover interval |  |  | * |  |  | (12) |
| 35 |  |  |  |  | Bed turnover interval |  |  | * |  |  | (23) |
| 36 |  |  |  | **Length of stay** | Length of stay =Time that the patient pass in hospital from the entrance to the exit |  |  | * |  |  | (13) |
| 37 |  |  |  |  | Length of stay |  |  | * |  |  | (24) |
| 38 |  |  |  |  | Length of stay |  |  | * |  |  | (25) |
| 39 |  |  |  |  | Length of stays= All inpatient medical expenses declared by the hospital (ex. Western medicine, Chinese medicine, dentist, dialysis, etc.) in the second-generation storage and admission detail files of the NHI administration. |  |  | * |  |  | (26) |
| 40 |  |  |  |  | ICU length of stay |  |  | * |  |  | (15) |
| 41 |  |  |  |  | Length of Stay (ALOS) =A number of calendar days of a patient's admission to discharge. It is calculated after they are discharged from the hospital |  |  | * |  |  | (7) |
| 42 |  |  |  |  | Length of stay = Length of stay at the hospital, e.g., average length of stay, rate of length of stay exceeding 14 days, per cent long stay patient |  |  | * |  |  | (5) |
| 43 |  |  |  |  | Length of stay =Time that the patient passes in hospital from the entrance to the exit |  |  | * |  |  | (16) |
| 44 |  |  |  |  | Length of stay |  |  | * |  |  | (27) |
| 45 |  |  |  |  | LOS of non-admitted patients |  |  | * |  |  | (28) |
| 46 |  |  |  |  | Length of stay |  |  | * |  |  | (29) |
| **Row** | **Performance aspects** | **Category** | **Sub-category** | **Indicators** | **Sub-indicators** | **Input** | **Process** | **Output** | **Outcome** | **Effect** | **Ref** |
| 47 | **Productivity** | **Organizational Management** | **Bed Utilization Rate** | **Length of stay** | Length of stay (LOS) of admitted patients |  |  | * |  |  | (28) |
| 48 |  |  |  |  | Length of Stay – All ER Patients |  |  | * |  |  | (30) |
| 49 |  |  |  |  | Length of Stay – Patients Discharged Home |  |  | * |  |  | (30) |
| 50 |  |  |  |  | Length of Stay – Patients Admitted to Hospital |  |  | * |  |  | (30) |
| 51 |  |  |  |  | Days of stay (ordinary, day hospital, day surgery) |  |  | * |  |  | (31) |
| 52 |  |  |  |  | Length of stay |  |  | * |  |  | (32) |
| 53 |  |  |  |  | Length of stay in medical imaging ward |  |  | * |  |  | (32) |
| 54 |  |  |  |  | Length of hospital stay |  |  | * |  |  | (33) |
| 55 |  |  |  |  | Length of ED stay for non-admitted patients |  |  | * |  |  | (34) |
| 56 |  |  |  |  | Md to exit minutes =Length of emergency department stay in minutes |  |  | * |  |  | (35) |
| 57 |  |  |  |  | Average length of stay (ALS) |  |  | * |  |  | (11) |
| 58 |  |  |  |  | Average length of stay |  |  | * |  |  | (8) |
| 59 |  |  |  |  | ALoS = Number of days occupied − number of beds/number of patients discharged and deceased. |  |  | * |  |  | (10) |
| 60 |  |  |  |  | Average time of hospitalization (days) |  |  | * |  |  | (36) |
| 61 |  |  |  |  | Mean length of stay in emergency department |  |  | * |  |  | (8) |
| 62 |  |  |  |  | Average of length of stay (LOS) |  |  | * |  |  | (23) |
| 63 |  |  |  |  | Average quarter length of stay to average LOS ratio= Quarterly average patients LOS compared to the country or region’s normative average LOS for the ward’s specialty |  |  | * |  |  | (37) |
| 64 |  |  |  |  | Average length of hospital stay for AMI |  |  | * |  |  | (38) |
| 65 |  |  |  |  | Mean length of stay: represents the time spent hospitalized. This indicator usually varies according to the diagnosis and profile of the patient, technological development, and payment mechanisms. Payments per procedure generally encourage stay periods to be reduced, an opposite situation to when it is paid for on a per day basis. |  |  | * |  |  | (1) |
| **Row** | **Performance aspects** | **Category** | **Sub-category** | **Indicators** | **Sub-indicators** | **Input** | **Process** | **Output** | **Outcome** | **Effect** | **Ref** |
| 66 | **Productivity** | **Organizational Management** | **Bed Utilization Rate** | **Length of stay** | Average number of days in hospital, ≤15 days |  |  | * |  |  | (22) |
| 67 |  |  |  |  | Average length of stay (ALOS) |  |  | * |  |  | (6) |
| 68 |  |  |  |  | Average rate of hospital days per patient |  |  | * |  |  | (39) |
| 69 |  |  |  |  | Mean length of stay in the Post-Anesthesia Care Unit (PACU) |  |  | * |  |  | (20) |
| 70 |  |  |  |  | Average patient stay |  |  | * |  |  | (12) |
| 71 |  |  |  |  | Average hospital days for inpatients |  |  | * |  |  | (2) |
| 72 |  |  |  |  | ALOS, average length of stay |  |  | * |  |  | (4) |
| 73 |  |  |  |  | Average hospital stay=The average time which a patient stays in the hospital |  |  | * |  |  | (40) |
| 74 |  |  |  |  | Average length of stay |  |  | * |  |  | (19) |
| 75 |  |  |  |  | Average length of stay =Average number of days patients spent in a hospital |  |  | * |  |  | (3) |
| 76 |  |  |  |  | The Average length of stay= indicates the stay in a health facility by patients occupying a bed for more than 24 h, for diagnosis, treatment, or care palliative, in days’ number. It can be considered an organizational barrier to access |  |  | * |  |  | (17) |
| 77 |  |  |  |  | Average length of hospital stay for CVI |  |  | * |  |  | (38) |
| 78 |  |  |  |  | Average hospitalization days |  |  | * |  |  | (21) |
| 79 |  |  |  |  | Relative stay index (RSI)=RSI is calculated as the number of days spent in hospital for selected diagnostic-related groups (DRGs) divided by the expected number of days spent in hospital (calculated as the average rates across the 35 New Zealand hospitals for 2001–2009) and standardized by age and case-mix. An RSI greater than 1 for a hospital indicates that length of stay is higher than expected given the case-mix of admissions to that hospital. An RSI of less than 1 indicates that the length of stay was less than expected |  |  | * |  |  | (41) |
| 80 |  |  |  |  | Relative stay index (case-mix adjusted differential average LOS days)= Measure of the average difference from the standard LOS for admitted patients with adjustments for case-mix. |  |  | * |  |  | (42) |
| **Row** | **Performance aspects** | **Category** | **Sub-category** | **Indicators** | **Sub-indicators** | **Input** | **Process** | **Output** | **Outcome** | **Effect** | **Ref** |
| 81 | **Productivity** | **Organizational Management** | **Bed Utilization Rate** | **Length of stay** | Inpatient care days |  |  | * |  |  | (43) |
| 82 |  |  |  |  | N° stays (ordinary, day hospital, day surgery) |  |  | * |  |  | (44) |
| 83 |  |  |  |  | Number of inpatient stays (stays, admissions, discharges, patient days, inpatient-day |  |  | * |  |  | (29) |
| 84 |  |  |  |  | Rate of inpatients staying for more than 30 days. Hospital prolonged length of stay has consequences for the healthcare provided effectiveness and the patient health status quality. The increase in hospitalization days’ results in a higher risk of infection and deterioration in treatment quality. Therefore, 30 days may not be adequate |  |  | * |  |  | (17) |
| 85 |  |  |  |  | Actual vs. expected length of stay |  |  | * |  |  | (45) |
| 86 |  |  |  |  | Number of bed days |  |  | * |  |  | (29) |
| 87 |  |  |  |  | Active beds-days= This refers to the number of functional beds in the hospital for a given period, usually 1 year, and it is obtained as number of active beds multiply by 365 days |  |  | * |  |  | (14) |
| 88 |  |  | **Hospital Characteristics** | **Case-mix index** | Case-mix index (CMI)= Hospital DRG-points divided by number of patients |  |  | * |  |  | (46) |
| 89 |  |  |  |  | The case mix indexes (CMIs) |  |  | * |  |  | (27) |
| 90 |  |  |  |  | Service complexity |  |  | * |  |  | (29) |
| 91 |  |  |  | **Number of operations/ procedures** | Number of operations/ procedures= Number of operations/procedures carried out in the hospital per operator/year |  |  | * |  |  | (5) |
| 92 |  |  |  |  | Diagnostic procedures= Total number of technical and diagnostic procedures |  |  | * |  |  | (47) |
| 93 |  |  |  |  | The total RVUs for outpatient services= The total number of acute bed days and chronic bed days in each hospital in the 2nd generation storage and inpatient detail files of the NHI administration, except for when the declaration field “Not applicable to Taiwan Diagnosis elated Groups (Tw-DRGs) Case Special Note” reads “9: Cases of declared cut accounts that have not been discharged within 30 days of hospitalization”, which is not included in the calculation. |  |  | * |  |  | (26) |
| **Row** | **Performance aspects** | **Category** | **Sub-category** | **Indicators** | **Sub-indicators** | **Input** | **Process** | **Output** | **Outcome** | **Effect** | **Ref** |
| 94 | **Productivity** | **Organizational Management** | **Quality Improvement** | **Surgery delay** | Late starts %= Cases started more than 15 min late from scheduled time as a percentage of total cases. Measurement is based on the actual and planned time of first incision |  |  |  | * |  | (48) |
| 95 |  |  |  |  | Delay for Elective Surgical Admission |  |  |  | * |  | (49) |
| 96 |  |  |  |  | Delay to hip fracture surgery |  |  |  | * |  | (45) |
| 97 |  |  |  |  | Mean delay time in the start of surgeries (with only times longer than 20 minutes being considered as delays by the IRC) |  |  |  | * |  | (20) |
| 98 |  |  |  | **Cancellation of Surgery** | % Canceled surgeries |  |  |  | * |  | (8) |
| 99 |  |  |  |  | Rate of Cancellation of Elective Surgery |  |  |  | * |  | (49) |
| 100 |  |  |  |  | Rate and reasons for cancellations of surgeries |  |  |  | * |  | (20) |
| 101 |  |  |  |  | Absenteeism rate |  |  |  | * |  | (20) |
| 102 |  |  |  |  | Cancelations %=Cases canceled on the operating day as a percentage of total cases |  |  |  | * |  | (48) |
| 103 |  |  |  |  | Number of unscheduled surgical re-interventions |  |  |  | * |  | (20) |
| 104 |  |  |  |  | Cancellation rate=the cancellation rate, which refers to the number of operations scheduled to be performed under the MOS system that were cancelled |  |  |  | * |  | (50) |
| 105 |  |  |  |  | Suspension rate=which indicates the number of patients who were not admitted and were not operated on for some reason |  |  |  | * |  | (50) |
| 106 |  |  |  |  | Substitution rate (SR)=defined as the proportion of potentially outpatient procedures performed under the MOS system with respect to the total number of such procedures scheduled |  |  |  | * |  | (50) |
| 107 |  |  |  |  | Surgical Referrals Out |  |  |  | * |  | (49) |
| 108 |  |  |  | **Lab Cancellation Rate** | Rate of cancelled analyses over a given period |  |  |  | * |  | (32) |
| 109 |  |  |  |  | number of cancelled analyses over a given period |  |  |  | * |  | (32) |
| 110 |  |  |  |  | Number of cancelled analyses over a given period |  |  |  | * |  | (32) |
| **Row** | **Performance aspects** | **Category** | **Sub-category** | **Indicators** | **Sub-indicators** | **Input** | **Process** | **Output** | **Outcome** | **Effect** | **Ref** |
| 111 | **Productivity** | **Clinical Management** | **Service delivery and Treatment** | **Hospitalization rate (HR)** | Number Of Patients= Number of departmental/specialty discharges | * |  |  |  |  | (46) |
| 112 |  |  |  |  | No. of patients= The number of patients admitted | * |  |  |  |  | (51) |
| 113 |  |  |  |  | Number of patients | * |  |  |  |  | (29) |
| 114 |  |  |  |  | Number of patients =Total number of patient discharges adjusted for case-mix with Roemer Index | * |  |  |  |  | (47) |
| 115 |  |  |  |  | Number of patients who did not-wait |  | * |  |  |  | (34) |
| 116 |  |  |  |  | Patients hospitalized |  |  | * |  |  | (52) |
| 117 |  |  |  |  | Hospitalization rate (HR)=which indicates the proportion of patients who required hospitalization because they could not be discharged after undergoing surgery under the MOS system |  |  | * |  |  | (50) |
| 118 |  |  |  |  | Admitted to hospital=Based on exit code. Admitted if exit code is admission, ICU |  |  | * |  |  | (35) |
| 119 |  |  |  |  | The number of hospital admissions | * |  |  |  |  | (23) |
| 120 |  |  |  |  | Percentage of ER Patients Admitted to Hospital |  |  | * |  |  | (30) |
| 121 |  |  |  |  | Inpatient admissions= Total number of inpatient admissions | * |  |  |  |  | (3) |
| 122 |  |  |  |  | The patient population over time | * |  |  |  |  | (27) |
| 123 |  |  |  | **In‑patient visits (IVs)** | In‑patient visits (IVs) |  |  | * |  |  | (6) |
| 124 |  |  |  |  | Number of clinical examinations |  |  | * |  |  | (29) |
| 125 |  |  |  |  | Obstetrics and Gynecology Admissions | * |  |  |  |  | (4) |
| 126 |  |  |  | **ER Visits** | Number of emergency visits |  |  | * |  |  | (29) |
| 127 |  |  |  |  | Total Number of ER Visits |  |  | * |  |  | (30) |
| 128 |  |  |  |  | Average Daily ER Visits |  |  | * |  |  | (30) |
| 129 |  |  |  |  | Emergency visits (EVs) |  |  | * |  |  | (6) |
| 130 |  |  |  |  | ED visits/emergency admissions rate=It was used to highlight disparities of access to outpatient care |  |  | * |  |  | (53) |

| **Row** | **Performance aspects** | **Category** | **Sub-category** | **Indicators** | **Sub-indicators** | **Input** | **Process** | **Output** | **Outcome** | **Effect** | **Ref** |
| --- | --- | --- | --- | --- | --- | --- | --- | --- | --- | --- | --- |
| 131 | **Productivity** | **Clinical Management** | **Service delivery and Treatment** | **ER Visits** | Frequent ED visits=When 4 or more ED visits occurred by an individual per year |  |  | * |  |  | (53) |
| 132 |  |  |  |  | Rate of admission via emergency department | * |  |  |  |  | (2) |
| 133 |  |  |  | **Outpatient visits** | Medical examinations in specialist offices |  |  | * |  |  | (43) |
| 134 |  |  |  |  | No. of outpatient visits= Total number of outpatient visits |  |  | * |  |  | (51) |
| 135 |  |  |  |  | Out‑patient visits (OVs) |  |  | * |  |  | (6) |
| 136 |  |  |  |  | Outpatient department visits =Total number of outpatient visits |  |  | * |  |  | (3) |
| 137 |  |  |  |  | Number of outpatient visits |  |  | * |  |  | (29) |
| 138 |  |  |  |  | Number of outpatient and emergency visits |  |  | * |  |  | (29) |
| 139 |  |  |  |  | Rate of first medical appointments within time. There is a legislated maximum guaranteed time for (non-urgent) the first appointments in hospitals after the query appointment request. This indicator assesses the users’ proportion with their first appointment within the maximum period established. |  |  | * |  |  | (17) |
| 140 |  |  |  |  | Number of patients reviewed or seen at clinic |  |  | * |  |  | (54) |
| 141 | **Efficiency** | **Organizational Management** | **Resource management** | **ED Bed numbers** | Beds number | * |  |  |  |  | (4) |
| 142 |  |  |  |  | Bed =The number of hospital beds representing organizational size | * |  |  |  |  | (55) |
| 143 |  |  |  |  | N° beds (ordinary, day hospital) | * |  |  |  |  | (56) |
| 144 |  |  |  |  | Number of beds | * |  |  |  |  | (9) |
| 145 |  |  |  |  | No. of beds=Number of beds in facility to the date 31.12 of reporting period | * |  |  |  |  | (51) |
| 146 |  |  |  |  | Number of hospital beds | * |  |  |  |  | (25) |
| 147 |  |  |  |  | Number of beds=The total number of staffed operational beds in the hospitals | * |  |  |  |  | (47) |
| 148 |  |  |  |  | Number of beds | * |  |  |  |  | (19) |
| 149 |  |  |  |  | Beds =Total number of beds available | * |  |  |  |  | (3) |

| **Row** | **Performance aspects** | **Category** | **Sub-category** | **Indicators** | **Sub-indicators** | **Input** | **Process** | **Output** | **Outcome** | **Effect** | **Ref** |
| --- | --- | --- | --- | --- | --- | --- | --- | --- | --- | --- | --- |
| 150 | **Efficiency** | **Organizational Management** | **Resource management** | **ED Bed numbers** | The number of beds (b) | * |  |  |  |  | (57) |
| 151 |  |  |  |  | Number of beds | * |  |  |  |  | (29) |
| 152 |  |  |  |  | Hospital beds | * |  |  |  |  | (43) |
| 153 |  |  |  |  | Total hospital beds = The total number of hospital beds, including emergency room beds, hemodialysis beds, nursery beds, obstetric wards, other observation beds, peritoneal dialysis beds, and so forth. | * |  |  |  |  | (26) |
| 154 |  |  |  | **Bed number by types** | Acute Beds (%) | * |  |  |  |  | (58) |
| 155 |  |  |  |  | Active beds | * |  |  |  |  | (11) |
| 156 |  |  |  |  | Number of active beds=This refers to number of functional beds for each hospital-year | * |  |  |  |  | (14) |
| 157 |  |  |  |  | Average Number of Active ER Beds | * |  |  |  |  | (30) |
| 158 |  |  |  |  | Average number of open beds | * |  |  |  |  | (22) |
| 159 |  |  |  |  | Average Available Inpatient Beds | * |  |  |  |  | (30) |
| 160 |  |  |  |  | The number of available beds | * |  |  |  |  | (23) |
| 161 |  |  |  |  | Average Available ICU Beds | * |  |  |  |  | (30) |
| 162 |  |  |  |  | Percentage of specialized beds= Demonstrates the high complexity and resolvability (intensive care, intermediaries and isolation) in the area of available beds. | * |  |  |  |  | (1) |
| 163 |  |  |  |  | ED beds | * |  |  |  |  | (52) |
| 164 |  |  |  |  | ICU beds | * |  |  |  |  | (52) |
| 165 |  |  |  | **Bed-staff ratios** | Beds-nurses ratio, 1:0.4 | * |  |  |  |  | (22) |
| 166 |  |  |  |  | Ratio of beds to nurses | * |  |  |  |  | (21) |
| 167 |  |  |  |  | Nurse/bed ratio | * |  |  |  |  | (2) |
| 168 |  |  |  |  | Ratio of beds to nurses =Total number of beds per nurse | * |  |  |  |  | (3) |

| **Row** | **Performance aspects** | **Category** | **Sub-category** | **Indicators** | **Sub-indicators** | **Input** | **Process** | **Output** | **Outcome** | **Effect** | **Ref** |
| --- | --- | --- | --- | --- | --- | --- | --- | --- | --- | --- | --- |
| 169 | **Efficiency** | **Organizational Management** | **Resource management** | **Bed-staff ratios** | Ratio of hospital staff per bed: Institutions with a lower hospital staff per bed ratio are generally more productive.18 However, this may in turn indicate a lower quality in care. | * |  |  |  |  | (1) |
| 170 |  |  |  |  | Total Employees/Beds (log) | * |  |  |  |  | (58) |
| 171 |  |  |  | **Bed-Physician ratios** | Ratio of beds to physicians= Total number of beds per physician | * |  |  |  |  | (3) |
| 172 |  |  |  |  | Daily number of hospitalization bed-days for each doctor |  |  | * |  |  | (22) |
| 173 |  |  |  | **Bed-patients ratios** | Average number of patients per bed per year (persons) |  |  | * |  |  | (36) |
| 174 |  |  |  |  | Ratio of Daily ER Patients to ER Beds |  |  | * |  |  | (30) |
| 175 |  |  |  | **Staff-patients ratios** | ICUs Nurse To Patient Ratio |  |  | * |  |  | (15) |
| 176 |  |  |  |  | Nurse staffing per patient seen |  |  | * |  |  | (52) |
| 177 |  |  |  |  | Ratio of Daily ER Patients to ER Staff |  |  | * |  |  | (30) |
| 178 |  | **Clinical Management** |  |  | Percentage of sick time=The proportion of full-time patient care personnel hours that were paid sick hours Sick hours * 100 / Full-time earned ho |  |  | * |  |  | (59) |
| 179 |  |  |  |  | Standard patients per FTE nurse |  |  | * |  |  | (17) |
| 180 |  |  |  |  | Standard patients per FTE nurse is also an indicator of the entity’s availability of physical resources, in this case, of nurses |  |  | * |  |  | (17) |
| 181 |  | **Organizational Management** |  | **Physician-patients ratios** | Physician staffing per patient seen |  |  | * |  |  | (52) |
| 182 |  |  |  |  | Daily number of clinic patients for each doctor |  |  | * |  |  | (22) |

| **Row** | **Performance aspects** | **Category** | **Sub-category** | **Indicators** | **Sub-indicators** | **Input** | **Process** | **Output** | **Outcome** | **Effect** | **Ref** |
| --- | --- | --- | --- | --- | --- | --- | --- | --- | --- | --- | --- |
| 183 | **Efficiency** | **Clinical Management** | **Resource management** | **Physician-patients ratios** | Standard patients per Full-time Equivalent (FTE) doctor is an indicator of physical availability resources (doctors) in hospitals. High values of this indicator indicate doctors’ occupation and a barrier to health care access. This variable, expressed as a function of the standard patient, allows the comparison between different entities. The standard patient’s calculation is based on the hospital transformation activity, by heterogeneous nature, into a single production unit. |  |  | * |  |  | (17) |
| 184 |  |  |  |  | Standard patients per FTE doctor |  |  | * |  |  | (17) |
| 185 |  | **Organizational Management** |  | **Exams-Physicians ratios** | Number of exams relative to the number of physicians |  |  | * |  |  | (32) |
| 186 |  |  |  |  | Number of exams relative to the number of radiologists |  |  | * |  |  | (32) |
| 187 |  |  | **Human resource management** | **Medical Staff numbers** | The number of nurses | * |  |  |  |  | (23) |
| 188 |  |  |  |  | Registered nurses (in%)= The proportion of nursing care hours that were provided by registered nurses Acute inpatient registered nursing earned hours * 100 / Acute inpatient nursing earned hours | * |  |  |  |  | (59) |
| 189 |  |  |  |  | Nurses= Total number of nurses available | * |  |  |  |  | (3) |
| 190 |  |  |  |  | Full-time RN (%) | * |  |  |  |  | (58) |
| 191 |  |  |  |  | Number of Medical Officers and Assistant Medical Officers | * |  |  |  |  | (19) |
| 192 |  |  |  |  | Nurse staffing | * |  |  |  |  | (52) |
| 193 |  |  |  |  | Number of nursing staff | * |  |  |  |  | (29) |
| 194 |  |  |  |  | No. of nurses=Registered number converted to full-time jobs in professions of nurse and midwife | * |  |  |  |  | (51) |
| 195 |  |  |  |  | No. of Nurses | * |  |  |  |  | (11) |
| 196 |  |  |  |  | Number of people in the care area | * |  |  |  |  | (25) |
| 197 |  |  |  |  | Emergency department staffing= nurses (full-time equivalent) per patients | * |  |  |  |  | (28) |

| **Row** | **Performance aspects** | **Category** | **Sub-category** | **Indicators** | **Sub-indicators** | **Input** | **Process** | **Output** | **Outcome** | **Effect** | **Ref** |
| --- | --- | --- | --- | --- | --- | --- | --- | --- | --- | --- | --- |
| 198 | **Efficiency** | **Organizational Management** | **Human resource management** | **Medical Staff numbers** | Number of medical & nursing staff | * |  |  |  |  | (29) |
| 199 |  |  |  |  | Percentage of health technicians (%), ≥75% | * |  |  |  |  | (22) |
| 200 |  |  |  |  | % of Health technical professionals in all employees | * |  |  |  |  | (21) |
| 201 |  |  |  |  | Number of medical staff | * |  |  |  |  | (9) |
| 202 |  |  |  |  | Number of medical staff | * |  |  |  |  | (29) |
| 203 |  |  |  |  | ICU Human resources= Exclusive routine physician per 10 beds or fraction during every shift | * |  |  |  |  | (60) |
| 204 |  |  |  |  | Average Number of ER Staff | * |  |  |  |  | (30) |
| 205 |  |  |  | **Non-Medical Staff numbers** | Number of people in the administrative area | * |  |  |  |  | (25) |
| 206 |  |  |  |  | Total Employees (log) | * |  |  |  |  | (58) |
| 207 |  |  |  |  | Number of health workers | * |  |  |  |  | (19) |
| 208 |  |  |  |  | The number of health workers (hw) | * |  |  |  |  | (57) |
| 209 |  |  |  |  | Labor | * |  |  |  |  | (29) |
| 210 |  |  |  |  | Number of non-medical non-nursing staff | * |  |  |  |  | (29) |
| 211 |  |  |  |  | Number of non-medical staff | * |  |  |  |  | (29) |
| 212 |  |  |  |  | Midlevel provider staffing | * |  |  |  |  | (52) |
| 213 |  |  |  |  | Number of permanent staff roles= Staff Roles: Which of the following staff roles currently exist in your organization? (Nurse practitioner, Nurse specialist, Nurse educator in ED, Staff for professional practice, Clinical specialist, Hospitalist, Social worker, Case manager, Staff for physician recruitment, Volunteer coordinator, Decision support role, Telehealth coordinator, Utilization review analyst, Risk management analyst, Staff for equity issues, Ombudsperson) | * |  |  |  |  | (59) |

| **Row** | **Performance aspects** | **Category** | **Sub-category** | **Indicators** | **Sub-indicators** | **Input** | **Process** | **Output** | **Outcome** | **Effect** | **Ref** |
| --- | --- | --- | --- | --- | --- | --- | --- | --- | --- | --- | --- |
| 214 | **Efficiency** | **Organizational Management** | **Human resource management** | **Non-Medical Staff numbers** | Some or most professionals=What percentage of physicians with administrative roles participated in continuing education activities (e.g., formal in-service programs, internal/external courses and conferences) supported by your organization? What percentage of nursing staff participated in continuing education activities (e.g., formal in-service programs, internal/external courses and conferences) supported by your organization? What percentage of other patient care staff participated in continuing education activities (e.g., formal in-service programs, internal/external courses and conferences) supported by your organization? | * |  |  |  |  | (59) |
| 215 |  |  |  |  | Number of other personnel= The total number of other personnel (non-medical or nurses) who are full time employees in the hospitals | * |  |  |  |  | (47) |
| 216 |  |  |  |  | Number of other allied professionals | * |  |  |  |  | (29) |
| 217 |  |  |  |  | Other stuff= Registered number converted to full-time work - health care workers without professions of doctor, dentist, nurse, midwife | * |  |  |  |  | (51) |
| 218 |  |  |  |  | Other Professionals | * |  |  |  |  | (11) |
| 219 |  |  |  |  | % of Vice-senior titles or above in health technical professionals | * |  |  |  |  | (21) |
| 220 |  |  |  |  | Number of administrative staff | * |  |  |  |  | (29) |
| 221 |  |  |  |  | Total staff | * |  |  |  |  | (29) |
| 222 |  |  |  | **Physicians number** | No. of Physicians | * |  |  |  |  | (11) |
| 223 |  |  |  |  | No. of doctors= Registered number converted to full-time work in professions of doctor and dentist | * |  |  |  |  | (51) |
| 224 |  |  |  |  | Hospital doctors | * |  |  |  |  | (43) |
| 225 |  |  |  |  | Doctors =Total number of doctors (specialists and primary care physicians) | * |  |  |  |  | (3) |
| 226 |  |  |  |  | Number of board-certified physicians | * |  |  |  |  | (2) |
| 227 |  |  |  |  | Number of doctors =The total number of doctors who are full time employees (FTEs) in the hospitals | * |  |  |  |  | (47) |
| **Row** | **Performance aspects** | **Category** | **Sub-category** | **Indicators** | **Sub-indicators** | **Input** | **Process** | **Output** | **Outcome** | **Effect** | **Ref** |
| 228 | **Efficiency** | **Organizational Management** | **Human resource management** | **Physicians number** | Percentage of full-time attending physicians | * |  |  |  |  | (2) |
| 229 |  |  |  |  | BLS/ACLS/ATLS/PALS certification for all medical doctors working in ED (KPI outcome 80% of all doctors per any one certification) | * |  |  |  |  | (61) |
| 230 |  |  |  |  | Total physicians=The total number of Western and Chinese medicine practitioners and dentists in the latest hospital practice registration in the statistics file of the medical personnel category in the health care management subsystem | * |  |  |  |  | (26) |
| 231 |  |  |  | **Physicians-nurses ratio** | Doctors-nurses ratio, 1:2 | * |  |  |  |  | (22) |
| 232 |  |  |  |  | Overall rating- Nurses and doctors | * |  |  |  |  | (59) |
| 233 |  |  |  |  | Ratio of doctors to nurses | * |  |  |  |  | (21) |
| 234 |  |  | **Paramedical Assessment** | **Number of laboratory tests** | Number of laboratory tests |  |  | * |  |  | (29) |
| 235 |  |  |  |  | Labs (lab tests)= Count of number of tests performed for the following: Metabolic panel, comprehensive metabolic panel, complete blood count with differential count, blood culture, C-reactive protein. |  |  | * |  |  | (35) |
| 236 |  |  |  |  | Number of analyses over a given period |  |  | * |  |  | (32) |
| 237 |  |  |  |  | Number of analyses over a given period |  |  | * |  |  | (32) |
| 238 |  |  |  |  | number of exams relative to the number of physicians |  |  | * |  |  | (32) |
| 239 |  |  |  |  | number of exams per patient and per stay relative to the length of stay |  |  | * |  |  | (32) |
| 240 |  |  |  |  | Number of analyses per patient and per hospital stay |  |  | * |  |  | (32) |
| 241 |  |  |  |  | Mean number of analyses per patient and per day |  |  | * |  |  | (32) |
| 242 |  |  |  |  | Mean number of analyses per physician and per day |  |  | * |  |  | (32) |
| 243 |  |  |  |  | Mean number of blood samples per hospital stay |  |  | * |  |  | (32) |
| 244 |  |  |  |  | Mean number of blood samples per day of hospitalization |  |  | * |  |  | (32) |
| 245 |  |  |  | **Number of Imagines** | Number of CT (Computed Tomography devices) |  |  | * |  |  | (9) |
| **Row** | **Performance aspects** | **Category** | **Sub-category** | **Indicators** | **Sub-indicators** | **Input** | **Process** | **Output** | **Outcome** | **Effect** | **Ref** |
| 246 | **Efficiency** | **Organizational Management** | **Paramedical Assessment** | **Number of Imagines** | Head CT scan =Count of number of CT scan of the head? |  |  | * |  |  | (35) |
| 247 |  |  |  |  | Abdominal/pelvic CT scan =Did patient receive CT scan of the abdomen or pelvis? |  |  | * |  |  | (35) |
| 248 |  |  |  |  | Chest X-ray =Count of number of X-ray of the chest? |  |  | * |  |  | (35) |
| 249 |  |  |  |  | Abdominal X-ray =Count of number of X-ray of the abdomen? |  |  | * |  |  | (35) |
| 250 |  |  | **Medical Management** | **Number of Surgeries** | No. of Surgeries |  |  | * |  |  | (11) |
| 251 |  |  |  |  | Number of surgeries (NOS) |  |  | * |  |  | (6) |
| 252 |  |  |  |  | Number of inpatient surgeries |  |  | * |  |  | (29) |
| 253 |  |  |  |  | Number of outpatient surgeries |  |  | * |  |  | (29) |
| 254 |  |  |  |  | Surgical operation numbers |  |  | * |  |  | (2) |
| 255 |  |  |  |  | Total cases= Number of cases |  |  | * |  |  | (48) |
| 256 |  |  |  |  | Number of admissions in surgery |  |  | * |  |  | (19) |
| 257 |  |  |  |  | Number of surgical wards |  |  | * |  |  | (19) |
| 258 |  |  |  |  | Number of surgeries performed per month-team |  |  | * |  |  | (20) |
| 259 |  |  |  |  | Number of inpatient & outpatient surgeries |  |  | * |  |  | (29) |
| 260 |  |  |  |  | Surgical Volume |  |  | * |  |  | 9 |
| 261 |  |  |  |  | Number of major surgery performed |  |  | * |  |  | (19) |
| 262 |  |  |  |  | Mean number of surgeries per SR-day |  |  | * |  |  | (20) |
| 263 |  |  |  |  | Ratio between the number of SRs in use and the total number of SRs existing per day |  |  | * |  |  | (20) |
| 264 |  |  |  |  | Number of admissions per year |  |  | * |  |  | (19) |
| 265 |  |  |  |  | Number of surgeries performed |  |  | * |  |  | (20) |
| 266 |  |  |  |  | Ambulatory rate (AR)=which measures the proportion of surgical procedures performed on an outpatient basis in relation to the total number of surgical procedures performed |  |  | * |  |  | (50) |
| 267 |  |  |  |  | Emergency procedures in surgery |  |  | * |  |  | (19) |
| **Row** | **Performance aspects** | **Category** | **Sub-category** | **Indicators** | **Sub-indicators** | **Input** | **Process** | **Output** | **Outcome** | **Effect** | **Ref** |
| 268 | **Efficiency** | **Organizational Management** | **Medical Management** | **Number of Surgeries** | Emergency Surgical Access |  |  | * |  |  | (49) |
| 269 |  |  |  | **Number of coded diagnoses** | Number of coded diagnoses |  |  | * |  |  | (27) |
| 270 |  |  |  | **Number of deliveries** | Number of deliveries |  |  | * |  |  | (25) |
| 271 |  |  |  |  | Multiple birth |  |  | * |  |  | (62) |
| 272 |  |  |  | **Number of Cesarean birth** | Cesarean birth |  |  | * |  |  | (63) |
| 273 |  |  |  |  | Cesarean birth |  |  | * |  |  | (62) |
| 274 |  |  |  |  | Proportion of caesarean section |  |  |  | * |  | (42) |
| 275 |  |  |  |  | Caesarean-section deliveries= Total number of caesarean-section service provided |  |  | * |  |  | (3) |
| 276 |  |  |  |  | Cesarean section rate (without justification). According to the World Health Organization (WHO), cesarean sections, unless performed for justifiable medical reasons, should be avoided, as, like any surgery, they carry immediate and long-term risks |  |  | * |  |  | (17) |
| 277 |  |  |  | **Number of Medical records** | Number of referrals for research |  |  | * |  |  | (64) |
| 278 |  |  |  |  | Number of methods to informatics patients |  | * |  |  |  | (64) |
| 279 |  |  |  |  | Number of related books in the archive | * |  |  |  |  | (64) |
| 280 |  | **Administrative Management** | **Supportive Units Assessment** | **Facilities Management** | Average age of major facilities (e.g. chiller, boiler) | * |  |  |  |  | (65) |
| 281 |  |  |  |  | The facilities for families and visitors | * |  |  |  |  | (8) |
| 282 |  |  |  |  | Availability of fire services system | * |  |  |  |  | (65) |
| 283 |  |  |  |  | Availability of fire services system | * |  |  |  |  | (66) |
| 284 |  |  |  |  | Number of unwanted (false) fire incident calls per year |  |  |  | * |  | (65) |
| 285 |  |  |  |  | Availability of lift system | * |  |  |  |  | (66) |

| **Row** | **Performance aspects** | **Category** | **Sub-category** | **Indicators** | **Sub-indicators** | **Input** | **Process** | **Output** | **Outcome** | **Effect** | **Ref** | |
| --- | --- | --- | --- | --- | --- | --- | --- | --- | --- | --- | --- | --- |
| 286 | **Efficiency** | **Administrative Management** | **Supportive Units Assessment** | **Facilities Management** | Availability of lift system | * |  |  |  |  | (65) | |
| 287 |  |  |  |  | Work request response rate |  |  |  | * |  | (65) | |
| 288 |  |  |  |  | Work request response rate |  |  |  | * |  | (66) | |
| 289 |  |  |  |  | No. of accidents per year |  |  |  | * |  | (66) | |
| 290 |  |  |  |  | Number of accidents per year |  |  |  | * |  | (65) | |
| 291 |  |  |  |  | No. of statutory orders per year | * |  |  |  |  | (65) | |
| 292 |  |  |  |  | No. of statutory orders per 5 years | * |  |  |  |  | (65) | |
| 293 |  |  |  |  | No. of statutory orders per year | * |  |  |  |  | (66) | |
| 294 |  |  |  |  | Facility condition index (FCI) |  |  | * |  |  | (65) | |
| 295 |  |  |  |  | Zonal floor area ratio for Outpatient (m2) | * |  |  |  |  | (67) | |
| 296 |  |  |  |  | Zonal floor area ratio for Inpatient (m2) | * |  |  |  |  | (67) | |
| 297 |  |  |  |  | Zonal floor area ratio for Public area (m2) | * |  |  |  |  | (67) | |
| 298 |  |  |  |  | Zonal floor area ratio for Central supply (m2) | * |  |  |  |  | (67) | |
| 299 |  |  |  |  | Zonal floor area ratio for Funeral service (m2) | * |  |  |  |  | (67) | |
| 300 |  |  |  | **Supply Chain Management** | N° handled SKUs (stock, direct delivery) |  | * |  |  |  | (68) | |
| 301 |  |  |  |  | N° handled packs |  | * |  |  |  | (68) | |
| 302 |  |  |  |  | N° outgoing items |  | * |  |  |  | (68) | |
| 303 |  |  |  |  | Economic value outgoing items |  | * |  |  |  | (68) | |
| 304 |  |  |  |  | Economic value warehouse inventory |  | * |  |  |  | (68) | |
| 305 |  |  |  |  | Warehouse inventory turnover ratio |  | * |  |  |  | (68) | |
| 306 |  |  |  |  | Surplus inventory= Value of surplus inventory to total assets ratio |  |  | * |  |  | (13) | |
| 307 |  |  |  |  | N° material requests (ordinary, urgent) |  | * |  |  |  | (68) | |
| 308 |  |  |  |  | N° order lines (ordinary, urgent) |  | * |  |  |  | (68) | |
| **Row** | **Performance aspects** | **Category** | **Sub-category** | **Indicators** | **Sub-indicators** | **Input** | **Process** | **Output** | **Outcome** | **Effect** | **Ref** | |
| 309 | **Efficiency** | **Administrative Management** | **Supportive Units Assessment** | **Supply Chain Management** | Economic value ward inventory (drugs, diagnostic material, medical devices, dialysis material) |  |  | * |  |  | (69) | |
| 310 |  |  |  |  | LSP revenue (ordinary/urgent service, computerized prescription and administration, hospitalization services. Includes VAT) |  |  | * |  |  | (70) | |
| 311 |  |  |  |  | Inventory availability=Accessible services and products | * |  |  |  |  | (40) | |
| 312 |  |  |  |  | Information availability=The capacity of information technology to demonstrate accurate data in the whole supply chain |  | * |  |  |  | (40) | |
| 313 |  |  |  |  | Innovation and technology= Supply chain buildings, facilities, services, and products | * |  |  |  |  | (40) | |
| 314 |  |  |  |  | Frequency of incorrect invoices=number of incorrect invoices/number of received invoices*100 |  |  |  | * |  | (71) | |
| 315 |  |  |  |  | Terms of Reference (TOR))=(beginning inventory+ purchase- ending inventory)/average inventory |  | * |  |  |  | (71) | |
| 316 |  |  |  |  | Frequency of delayed payment at the due date=Difference between payment date and due date (days)/Due date*100 |  | * |  |  |  | (71) | |
| 317 |  |  |  |  | response Time=Deliver the on-time services and products |  | * |  |  |  | (40) | |
| 318 |  |  |  |  | Weekly time spent on logistics activities |  | * |  |  |  | (58) | |
| 319 |  |  |  | **Medical Equipment measures** | Number of medical equipment together | * |  |  |  |  | (9) | |
| 320 |  |  |  |  | Number of exams relative to the number of available machines |  |  | * |  |  | (32) | |
| 321 |  |  |  |  | Number of technicians per number of capital devices | * |  |  |  |  | (72) | |
| 322 |  |  |  |  | Number of SM performed per number of capital devices | * |  |  |  |  | (72) | |
| 323 |  |  |  |  | No. of functional units | * |  |  |  |  | (73) | |
| 324 |  |  |  |  | Gross equipment = The gross amount of machinery and equipment in the balance sheet for public hospitals; the gross amount of medical equipment in the balance sheet for private hospitals | * |  |  |  |  | (26) | |
| 325 |  |  |  |  | Utilization rate of the Laboratory and Blood bank machines |  |  | * |  |  | (32) | |
| **Row** | **Performance aspects** | **Category** | **Sub-category** | **Indicators** | **Sub-indicators** | **Input** | **Process** | **Output** | **Outcome** | **Effect** | **Ref** | |
| 326 | **Efficiency** | **Administrative Management** | **Supportive Units Assessment** | **Number of Discharge** | Number of discharges |  | * |  |  |  | (25) | |
| 327 |  |  | **Financial Management** |  | No. of the Discharged |  | * |  |  |  | (11) | |
| 328 |  |  |  |  | Number of admissions or discharges in a given year |  | * |  |  |  | 6 | |
| 329 |  |  |  |  | Discharge transition |  | * |  |  |  | (59) | |
| 330 |  |  |  |  | Percentage of ER Patients Discharged Home |  | * |  |  |  | (30) | |
| 331 |  |  |  |  | Percentage of CVI patients discharged from the hospital to a rehabilitation facility (%rehabilitation-cvi) |  | * |  |  |  | (38) | |
| 332 |  |  |  |  | Hospital output percentage by external transfer: indicates the percentage of hospital outputs that are a result of referrals to other institutions. High values usually point to low resolvability and a lack of structure that is necessary for appropriate patient treatment |  | * |  |  |  | (1) | |
| 333 |  |  |  |  | Medicaid Discharges (log) |  |  | * |  |  | (58) | |
| 334 |  |  |  |  | Medicare Discharges (log) |  |  | * |  |  | (58) | |
| 335 | **Effectiveness** | **Organizational Management** | **Patient Safety** | **Mortality rate** | Total hospital mortality rate (%) |  |  |  | * |  | (39) | |
| 336 |  |  |  |  | Hospital mortality rate= measures the proportion of patients who die during hospitalization. This indicator reflects the patient’s general state, complexity of the cases, resolvability and quality of care provided. The hospital mortality rate can also be associated with hospital reservation access and rates, admission type (emergency or voluntary), early discharges and severe cases being transferred to other institutions. |  |  |  | * |  | (1) | |
| 337 |  |  |  |  | Hospital mortality rate (HMR) |  |  |  | * |  | (6) | |
| 338 |  |  |  |  | Hospital Death Rate (HDR)= Percentage of patients discharged from the hospital alive or dead. It is the indicator of mortality in a healthcare facility |  |  |  | * |  | (7) | |
| 339 |  |  |  |  | Mortality/Death =Mortality/Death in healthcare organization |  |  |  | * |  | (16) | |
| 340 |  |  |  |  | Hospital deaths =HSMR (hospital standardized mortality ratio) |  |  |  | * |  | (74) | |
| **Row** | **Performance aspects** | **Category** | **Sub-category** | **Indicators** | **Sub-indicators** | **Input** | **Process** | **Output** | **Outcome** | **Effect** | **Ref** |  |
| 341 | **Effectiveness** | **Organizational Management** | **Patient Safety** | **Mortality rate** | In-hospital mortality =was used to reflect the quality of care during emergency care or surgery as reported in three articles identified in our review |  |  |  | * |  | (53) |  |
| 342 |  |  |  |  | Mortality during the birth hospitalization |  |  |  | * |  | (63) |  |
| 343 |  |  |  |  | Mortality during the birth hospitalization |  |  |  | * |  | (62) |  |
| 344 |  |  |  |  | Mortality rate |  |  |  | * |  | (8) |  |
| 345 |  |  |  |  | Mortality rate for all inpatients |  |  |  | * |  | (2) |  |
| 346 |  |  |  |  | Mortality |  |  |  | * |  | (24) |  |
| 347 |  |  |  |  | Mortality |  |  |  | * |  | (27) |  |
| 348 |  |  |  |  | Mortality (%) |  |  |  | * |  | (22) |  |
| 349 |  |  |  |  | Mortality/Death= Mortality/Death in healthcare organization, such as mortality of the patients discharged, stillbirths and infant deaths, deaths in hospital following surgery |  |  |  | * |  | (5) |  |
| 350 |  |  |  |  | Amenable mortality |  |  |  | * |  | (24) |  |
| 351 |  |  |  |  | Standardized mortality ratio (SMR) =The SMR corresponds to the ratio of the observed number of deaths to the expected number of deaths. The expected number of deaths was obtained by the sum of mortality probabilities obtained from the SAPS-3 standard equation or APACHE-IV risk models |  |  |  | * |  | (75) |  |
| 352 |  |  |  |  | Hospital Standardized Mortality Rao (HSMR)* |  |  |  | * |  | (45) |  |
| 353 |  |  |  |  | Mortality rate within the first 48 hours of hospitalization (%) |  |  |  | * |  | (39) |  |
| 354 |  |  |  |  | 30-day mortality=any admission that preceded the patient’s death (from any cause) by 30 days or less was considered a 30-day mortality admission |  |  |  | * |  | (41) |  |
| 355 |  |  |  |  | AMI with PTCA within 2 days=30-day mortality |  |  |  | * |  | (42) |  |
| 356 |  |  |  |  | AMI with PTCA after 2 days= 30-day mortality |  |  |  | * |  | (42) |  |
| 357 |  |  |  |  | Mortality 30days Last Admittance |  |  |  | * |  | (46) |  |
| 358 |  |  |  |  | PN morality rate (MORT_30_PN) |  |  |  | * |  | (69) |  |
| **Row** | **Performance aspects** | **Category** | **Sub-category** | **Indicators** | **Sub-indicators** | **Input** | **Process** | **Output** | **Outcome** | **Effect** | **Ref** |  |
| 359 | **Effectiveness** | **Organizational Management** | **Patient Safety** | **Mortality rate** | HF mortality rate (MORT_30_HF) |  |  |  | * |  | (69) |  |
| 360 |  |  |  |  | HA mortality rate (MORT_30_AMI) |  |  |  | * |  | (69) |  |
| 361 |  |  |  |  | Reversed 30-Day Mortality (outcome) |  |  |  | * |  | (58) |  |
| 362 |  |  |  |  | Valvuloplasty or heart valve replacement= 30-day mortality |  |  |  | * |  | (42) |  |
| 363 |  |  |  |  | Congestive heart failure= 30-day mortality |  |  |  | * |  | (42) |  |
| 364 |  |  |  |  | Ischemic stroke= 30-day mortality |  |  |  | * |  | (42) |  |
| 365 |  |  |  |  | Femur fracture= 30-day mortality |  |  |  | * |  | (42) |  |
| 366 |  |  |  |  | Colon cancer surgery= 30-day mortality |  |  |  | * |  | (42) |  |
| 367 |  |  |  |  | COPD exacerbation= 30-day mortality |  |  |  | * |  | (42) |  |
| 368 |  |  |  |  | AMI=30-day mortality |  |  |  | * |  | (42) |  |
| 369 |  |  |  |  | Isolated aortocoronary bypass= 30-day mortality |  |  |  | * |  | (42) |  |
| 370 |  |  |  |  | AMI without PTCA= 30-day mortality |  |  |  | * |  | (42) |  |
| 371 |  |  |  |  | Age and gender standardized CVI 30 days in-hospital (same hospital) mortality rate (mort-30-cvi) |  |  |  | * |  | (38) |  |
| 372 |  |  |  |  | Age and gender standardized AMI 30 days in-hospital (same hospital) mortality rate (mort-30-ami) |  |  |  | * |  | (38) |  |
| 373 |  |  |  |  | Mortality 90 days Last Admittance |  |  |  | * |  | (46) |  |
| 374 |  |  |  |  | Mortality 180 days Last Admittance |  |  |  | * |  | (46) |  |
| 375 |  |  |  |  | Mortality 365 days Last Admittance |  |  |  | * |  | (46) |  |
| 376 |  |  |  |  | AMI= 1-year mortality |  |  |  | * |  | (42) |  |
| 377 |  |  |  |  | Mortality rate for the intensive care unit patients |  |  |  | * |  | (2) |  |
| 378 |  |  |  |  | ICU mortality rate |  |  |  | * |  | (15) |  |
| 379 |  |  |  |  | Surgical mortality |  |  |  | * |  | (2) |  |
| 380 |  |  |  |  | Hospital deaths following major surgery |  |  |  | * |  | (74) |  |
| 381 |  |  |  |  | Operative mortality rate |  |  |  | * |  | (20) |  |
| 382 |  |  |  |  | Per-Operative Mortality Rate (POMR) |  |  |  | * |  | (49) |  |
| **Row** | **Performance aspects** | **Category** | **Sub-category** | **Indicators** | **Sub-indicators** | **Input** | **Process** | **Output** | **Outcome** | **Effect** | **Ref** |  |
| 383 | **Effectiveness** | **Organizational Management** | **Patient Safety** | **Mortality rate** | Case Fatality Rate for all surgical in-patients |  |  |  | * |  | (19) |  |
| 384 |  |  |  |  | failure to rescue rate=One distinguishes in-hospital mortality from failure to rescue. |  |  |  | * |  | (53) |  |
| 385 |  |  |  |  | Mortality rate to average mortality rate ratio =Half yearly or annual average patients mortality rate compared to the country or region’s normative average mortality rate for the ward’s specialty |  |  |  | * |  | (37) |  |
| 386 |  |  |  |  | Day mortality rate=mortality following emergency admission for hip fracture, reflecting quality of ED- and hospital-based care, as well as access to and quality of ambulatory follow-up care post-discharge |  |  |  | * |  | (53) |  |
| 387 |  |  |  |  | Case Fatality Rate for all in-patients |  |  |  | * |  | (19) |  |
| 388 |  |  |  |  | %CPR unsuccessful in ED |  |  |  | * |  | (76) |  |
| 389 |  |  |  | **Readmission rate (24h-72h RR)** | Readmission |  |  |  | * |  | (24) |  |
| 390 |  |  |  |  | Readmission rate overall |  |  |  | * |  | (45) |  |
| 391 |  |  |  |  | Readmission rates |  |  |  | * |  | (27) |  |
| 392 |  |  |  |  | Readmission rate=which measures the number of patients who were discharged after surgery under the MOS system and who required readmission in the hours or days following surgery due to some post-surgical complication |  |  |  | * |  | (50) |  |
| 393 |  |  |  |  | Medical readmissions (in%) |  |  |  | * |  | (59) |  |
| 394 |  |  |  |  | Medical patients readmitted to hospital |  |  |  | * |  | (74) |  |
| 395 |  |  |  |  | 24-h readmission rate |  |  |  | * |  | (60) |  |
| 396 |  |  |  |  | Revisit the emergency department 72 h after leaving |  |  |  | * |  | (2) |  |
| 397 |  |  |  |  | Revisit 72 hours= Patient returns within 72 h of a previous visit for Binary the same condition |  |  |  | * |  | (35) |  |
| 398 |  |  |  |  | Percentage of Revisits to Emergency Room within 3 days |  |  |  | * |  | (30) |  |
| 399 |  |  |  |  | Percentage of Revisits to Emergency Room within 7 days |  |  |  | * |  | (30) |  |
| 400 |  |  |  |  | Readmission within 14d after discharge for inpatients |  |  |  | * |  | (2) |  |
| **Row** | **Performance aspects** | **Category** | **Sub-category** | **Indicators** | **Sub-indicators** | **Input** | **Process** | **Output** | **Outcome** | **Effect** | **Ref** |  |
| 401 | **Effectiveness** | **Organizational Management** | **Patient Safety** | **Readmission rate (24h-72h RR)** | Unscheduled readmission/return=Unscheduled readmission/return to the hospital, e.g., readmissions within 28 days, readmission rate within 14 days, unexpected returns after transferred out |  |  |  | * |  | (5) |  |
| 402 |  |  |  |  | Rate of readmissions within 30 days after discharge. Hospital readmissions, when unplanned, can represent deficiencies in satisfying the needs corresponding to a given disease. Thus, it is relevant for hospital entities to identify an entity’s effectiveness to provide care and the patient’s ability to recover |  |  |  | * |  | (17) |  |
| 403 |  |  |  |  | 30-Day Readmission |  |  |  | * |  | (58) |  |
| 404 |  |  |  |  | Readmission within 30 after discharge |  |  |  | * |  | (33) |  |
| 405 |  |  |  |  | Readmission rate for AMI within 30 days of discharge (readmission 30-ami) |  |  |  | * |  | (38) |  |
| 406 |  |  |  |  | Readmission rate for CVI within 30 days of discharge (readmission-30-cvi) |  |  |  | * |  | (38) |  |
| 407 |  |  |  |  | Ischemic stroke= 30-day readmission |  |  |  | * |  | (42) |  |
| 408 |  |  |  |  | COPD=30-day readmission |  |  |  | * |  | (42) |  |
| 409 |  |  |  |  | Readm30_Inpatient=Patient admitted to inpatient care in hospital within 30 days of the discharge and at least two days after discharge |  |  |  | * |  | (46) |  |
| 410 |  |  |  |  | Readm30_Emergency= Patient admitted acutely to inpatient care in hospital within 30 days of the discharge |  |  |  | * |  | (46) |  |
| 411 |  |  |  |  | Unplanned readmissions=All inpatient hospital admissions that were both acute (i.e. non-elective) and occurred 30 days or less after the patient’s most recent inpatient discharge were considered unplanned readmissions. In line with other studies (for example, [29]), such admissions were not required to be for the same or related condition, nor were they required to be at the same hospital |  |  |  | * |  | (41) |  |
| 412 |  |  |  |  | Percentage of readmissions=Ratio of readmissions within 40 days of discharge, related to the same medical problem |  |  |  | * |  | (16) |  |
| **Row** | **Performance aspects** | **Category** | **Sub-category** | **Indicators** | **Sub-indicators** | **Input** | **Process** | **Output** | **Outcome** | **Effect** | **Ref** |  |
| 413 | **Effectiveness** | **Organizational Management** | **Patient Safety** | **Readmission rate (24h-72h RR)** | Percentage of readmissions=Ratio of readmissions within 40 days of discharge, related to the same medical problem |  |  |  | * |  | (13) |  |
| 414 |  |  |  |  | ICU readmission rate |  |  |  | * |  | (15) |  |
| 415 |  |  |  |  | Surgical patients readmitted to hospital |  |  |  | * |  | (74) |  |
| 416 |  |  |  |  | ED readmission rate/Emergency re-hospitalization rate=to analyze the rate of hospital admissions through the ED in the year following a diagnosis of cancer. |  |  |  | * |  | (53) |  |
| 417 |  |  |  |  | Obstetric patients readmitted to hospital |  |  |  | * |  | (74) |  |
| 418 |  |  |  |  | Patients aged 19 years and younger readmitted to hospital |  |  |  | * |  | (74) |  |
| 419 |  | **Clinical Management** |  |  | Re-hospitalization rate =Half yearly or annual average patients re-hospitalization rate compared to the country or region’s normative average rate for the ward’s specialty |  |  |  | * |  | (37) |  |
| 420 | **Speed** | **Organizational Management** | **Time Management** | **Waiting time** | Waiting time= Total of time that a patient wait in hospital |  | * |  |  |  | (13) |  |
| 421 |  |  |  |  | Waiting time =Waiting time for healthcare service, such as outpatient waiting times, waiting time for admission, waiting time for treatment |  | * |  |  |  | (5) |  |
| 422 |  |  |  |  | Waiting time = Total of time that a patient waits for an initial rehabilitation service |  | * |  |  |  | (16) |  |
| 423 |  |  |  |  | The average waiting time for admission |  | * |  |  |  | (64) |  |
| 424 |  |  |  |  | Average length of waiting for the first scheduled health check at the institution (days) |  | * |  |  |  | (39) |  |
| 425 |  |  |  |  | Average Door to Doctor Time (Waiting Time) |  | * |  |  |  | (30) |  |
| 426 |  |  |  |  | Waiting time before surgery indicates the time between patient admission and surgery, in the number of days, and can be considered an organizational barrier to access. |  | * |  |  |  | (17) |  |
| 427 |  |  |  |  | Surgical wait measures |  | * |  |  |  | (45) |  |
| 428 |  |  |  |  | Wait measure in emergency department |  | * |  |  |  | (45) |  |
| 429 |  |  |  |  | Average length of waiting for the first scheduled surgical check (days) |  | * |  |  |  | (39) |  |
| **Row** | **Performance aspects** | **Category** | **Sub-category** | **Indicators** | **Sub-indicators** | **Input** | **Process** | **Output** | **Outcome** | **Effect** | **Ref** |  |
| 430 | **Speed** | **Organizational Management** | **Time Management** | **Waiting time** | Emergency room (ER) waiting time |  | * |  |  |  | (8) |  |
| 431 |  |  |  |  | Average Number of ER Patients Waiting for Treatment |  | * |  |  |  | (30) |  |
| 432 |  |  |  |  | Average Number of ER Patients Waiting for Admission |  | * |  |  |  | (30) |  |
| 433 |  |  |  |  | Patient waiting times for Australasian Triage Scale categories 2, 3, 4 and 5 ((ATS category 2 (maximum wait time for medical assessment and treatment: 10 min), ATS category 3 (maximum wait time for medical, assessment and treatment: 30 min), ATS category 4 (maximum wait time for medical assessment and treatment: 1hour) ATS category 5 (maximum wait time for medical assessment and treatment: 2hours) |  | * |  |  |  | (34) |  |
| 434 |  |  |  |  | Patients waiting time in the radiology services |  | * |  |  |  | (32) |  |
| 435 |  |  |  |  | Wait measure for diagnose imaging (magnet resonance imaging/computerized tomography) |  | * |  |  |  | (45) |  |
| 436 |  |  |  | **Interval between discharge process** | Interval between discharge and billing |  | * |  |  |  | (64) |  |
| 437 |  |  |  |  | Doctor Decision to Patient Discharge (ER Bed Turnaround Time) |  | * |  |  |  | (30) |  |
| 438 |  |  |  |  | Discharge ED LOS < 4 hours |  | * |  |  |  | (52) |  |
| 439 |  |  |  | **Laboratory sample/report intervals** | Time between report of the result and reading of the result |  | * |  |  |  | (32) |  |
| 440 |  |  |  |  | Time between report of the result and consequential therapeutic change |  | * |  |  |  | (32) |  |
| 441 |  |  |  |  | time between sample collection and report of the result |  | * |  |  |  | (32) |  |
| 442 |  |  |  |  | Time between sample collection and arrival in the lab |  | * |  |  |  | (32) |  |
| 443 |  |  |  |  | Time between sample collection and report of the result |  | * |  |  |  | (32) |  |
| 444 |  |  |  |  | Time between sample collection and arrival in the lab |  | * |  |  |  | (32) |  |
| 445 |  |  |  |  | Number of exams over a given period |  | * |  |  |  | (32) |  |
| 446 |  |  |  |  | Time between sample collection and report of the result |  | * |  |  |  | (32) |  |

| **Row** | **Performance aspects** | **Category** | **Sub-category** | **Indicators** | **Sub-indicators** | **Input** | **Process** | **Output** | **Outcome** | **Effect** | **Ref** |
| --- | --- | --- | --- | --- | --- | --- | --- | --- | --- | --- | --- |
| 447 | **Speed** | **Organizational Management** | **Time Management** | **Laboratory sample/report intervals** | Time between sample collection and arrival in the lab |  | * |  |  |  | (32) |
| 448 |  |  |  |  | Time between sample collection and report of the result |  | * |  |  |  | (32) |
| 449 |  |  |  |  | Time between sample collection and arrival in the lab |  | * |  |  |  | (32) |
| 450 |  |  |  |  | Time between sample collection and report of the result (TAT) |  | * |  |  |  | (32) |
| 451 |  |  |  |  | Time between sample collection and arrival in the lab |  | * |  |  |  | (32) |
| 452 |  |  |  |  | Time between arrival in the lab and start of analysis |  | * |  |  |  | (32) |
| 453 |  |  |  |  | Time between start of analysis and technical validation |  | * |  |  |  | (32) |
| 454 |  |  |  |  | Time between technical validation and biological validation |  | * |  |  |  | (32) |
| 455 |  |  |  |  | Time between start of analysis and report of the result |  | * |  |  |  | (32) |
| 456 |  |  |  |  | Time between the report of a drug dosage result and the consequential therapeutic change |  | * |  |  |  | (32) |
| 457 |  |  |  |  | Time between the report of a hyperkalemia and the consequential therapeutic change |  | * |  |  |  | (32) |
| 458 |  |  |  |  | Time between report of the result and reading of the result |  | * |  |  |  | (32) |
| 459 |  |  |  | **Lab Turnaround time (TAT)** | Turnaround time (TAT)= TAT is the time from receipt of the sample in the laboratory to final delivery or dispatch of the report of said test |  | * |  |  |  | (77) |
| 460 |  |  |  |  | Turnaround time (TAT) in clinical chemistry and hematology tests |  | * |  |  |  | (78) |
| 461 |  |  |  |  | The Turn Around Time (TAT)= TAT of samples which improves the patient care in the organization. STAT tests are defined as tests to be reported in a short turnaround time, usually below 1 h. |  | * |  |  |  | (79) |
| 462 |  |  |  |  | Turnaround Time (TAT) of Blood Issues= Sum of the time taken for cross-match /(Total number of blood and blood components cross-matched∕ reserved) |  | * |  |  |  | (80) |
| 463 |  |  |  |  | Turnaround time and associated measures |  | * |  |  |  | (32) |
| 464 |  |  |  | **Imaging exam/result intervals** | Time between prescription and report of the result |  | * |  |  |  | (32) |
| **Row** | **Performance aspects** | **Category** | **Sub-category** | **Indicators** | **Sub-indicators** | **Input** | **Process** | **Output** | **Outcome** | **Effect** | **Ref** |
| 465 | **Speed** | **Organizational Management** | **Time Management** | **Imaging exam/result intervals** | Time between prescription and receipt of the request in the radiology service |  | * |  |  |  | (32) |
| 466 |  |  |  |  | Time between prescription and receipt of the request in the radiology service |  | * |  |  |  | (32) |
| 467 |  |  |  |  | Time between the end of the exam and the availability of the images for the requesting physician |  | * |  |  |  | (32) |
| 468 |  |  |  |  | Time between the end of the exam and the availability of a first report for the requesting physician |  | * |  |  |  | (32) |
| 469 |  |  |  |  | Time between the availability of the report and taking into account the results of the exam |  | * |  |  |  | (32) |
| 470 |  |  |  |  | Time between the end of the exam and the availability of the final report for the requesting physician |  | * |  |  |  | (32) |
| 471 |  |  |  |  | Exam length |  | * |  |  |  | (32) |
| 472 |  |  |  |  | Report writing time by the radiologist |  | * |  |  |  | (32) |
| 473 |  |  |  |  | Time between prescription and report of the result (TAT) |  | * |  |  |  | (32) |
| 474 |  |  |  |  | Time between prescription and receipt of the request in the radiology service |  | * |  |  |  | (32) |
| 475 |  |  |  |  | Time between receipt of the request and appointment scheduling |  | * |  |  |  | (32) |
| 476 |  |  |  |  | Time between appointment scheduling and execution of the exam |  | * |  |  |  | (32) |
| 477 |  |  |  |  | Timer between the scheduled time of the exam and the actual time of the exam |  | * |  |  |  | (32) |
| 478 |  |  |  |  | Number of exams over a given period |  | * |  |  |  | (32) |
| 479 |  |  |  |  | Time between receipt of the request and appointment scheduling, |  | * |  |  |  | (32) |
| 480 |  |  |  |  | Time between appointment scheduling and execution of the exam |  | * |  |  |  | (32) |
| 481 |  |  |  |  | Percentage of CVI patients with CT scan or MRI done within 3 h of admission (%CT-MRI-cvi) |  | * |  |  |  | (38) |
| 482 |  |  |  | **Time of surgery** | Average surgery time= Time from first incision to wound closure |  | * |  |  |  | (48) |
| 483 |  |  |  |  | Time of surgery |  | * |  |  |  | (31) |
| 484 |  |  |  |  | Overrun time %= Overrun hours as a percentage of total hours |  | * |  |  |  | (48) |
| **Row** | **Performance aspects** | **Category** | **Sub-category** | **Indicators** | **Sub-indicators** | **Input** | **Process** | **Output** | **Outcome** | **Effect** | **Ref** |
| 485 | **Speed** | **Organizational Management** | **Time Management** | **Time of surgery** | Total hours= Total time from patient entering to patient leaving the OR |  | * |  |  |  | (48) |
| 486 |  |  |  |  | Total surgery time= Total time from first incision to wound closure |  | * |  |  |  | (48) |
| 487 |  |  |  |  | Share of non-elective surgery= Non-elective cases as a percentage of total cases |  | * |  |  |  | (48) |
| 488 |  |  |  |  | Rate of surgeries within time. There is a legislated time waiting for surgery. This indicator assesses the proportion of registered patients waiting for surgical intervention within the maximum legal time. |  | * |  |  |  | (17) |
| 489 |  |  |  |  | Time between start of anesthesia and start of surgery |  | * |  |  |  | (31) |
| 490 |  |  |  |  | Time between end of surgery and end of anesthesia |  | * |  |  |  | (31) |
| 491 |  |  |  |  | Time between end of anesthesia and exit the operating |  | * |  |  |  | (31) |
| 492 |  |  |  |  | Time of patient in operating room |  | * |  |  |  | (31) |
| 493 |  |  |  |  | Time of anesthesia |  | * |  |  |  | (31) |
| 494 |  |  |  |  | Adequate timing for prophylactic antibiotics before surgery |  | * |  |  |  | (2) |
| 495 |  |  |  |  | Mean Duration of In-Hospital, Pre-Operative Stay |  | * |  |  |  | (49) |
| 496 |  |  |  |  | Rate of First Elective Case On Time Theater Performance |  | * |  |  |  | (49) |
| 497 |  |  |  |  | Time between entry into the operating room and start of anesthesia |  | * |  |  |  | (31) |
| 498 |  |  |  |  | Mean time for cleaning the SR in the interval between two surgeries |  | * |  |  |  | (20) |
| 499 |  |  |  |  | Size of the surgeries performed (by duration) |  | * |  |  |  | (20) |
| 500 |  |  |  |  | Standard Operating Times (SOTs) |  | * |  |  |  | (44) |
| 501 |  |  |  |  | Average turnover time=Time from patient leaving the OR to next patient entering the OR if scheduled in sequence |  | * |  |  |  | (48) |
| 502 |  |  |  |  | Mean turnover time in the SR (including only times below 60 minutes9); |  | * |  |  |  | (20) |
| 503 |  |  |  |  | Utilization= Raw utilization, calculated as (total hours – overrun time)/resource hours |  | * |  |  |  | (48) |

| **Row** | **Performance aspects** | **Category** | **Sub-category** | **Indicators** | **Sub-indicators** | **Input** | **Process** | **Output** | **Outcome** | **Effect** | **Ref** |
| --- | --- | --- | --- | --- | --- | --- | --- | --- | --- | --- | --- |
| 504 | **Speed** | **Organizational Management** | **Time Management** | **ED Requests Turnaround Time** | Average Registration Time |  | * |  |  |  | (30) |
| 505 |  |  |  |  | Average Arrival to Triage Time |  | * |  |  |  | (30) |
| 506 |  |  |  |  | Time from arrival to initial triage |  | * |  |  |  | (28) |
| 507 |  |  |  |  | Triage duration (hours) |  | * |  |  |  | (76) |
| 508 |  |  |  |  | Time to triage |  | * |  |  |  | (52) |
| 509 |  |  |  |  | Average Triage to Bed Time |  | * |  |  |  | (30) |
| 510 |  |  |  |  | The rate of emergency transfer in-patient stay over 48 h = Number of cases with >48 h in the emergency department/number of cases transferred from emergency department to admission) × 100%. |  | * |  |  |  | (26) |
| 511 |  |  |  |  | Average Doctor Examination to Decision Made (Treatment Time) |  | * |  |  |  | (30) |
| 512 |  |  |  |  | Percentage of ED green-coded patients visited within 1 hour= Measure of timely emergency care for ED patients whose treatment may be delayed without risk |  | * |  |  |  | (42) |
| 513 |  |  |  |  | Admit ED LOS < 6 hours |  | * |  |  |  | (52) |
| 514 |  |  |  |  | Percentage of ED patients referred for hospital admission with ED LOS≤8 hours= Measure of overall timely emergency care. |  | * |  |  |  | (42) |
| 515 |  |  |  |  | Percentage of ER Patients with LOS More than 6 hours |  | * |  |  |  | (30) |
| 516 |  |  |  |  | Average ER Lab Requests Turnaround Time |  | * |  |  |  | (30) |
| 517 |  |  |  |  | Time from arrival to chest radiography for admitted patients |  | * |  |  |  | (28) |
| 518 |  |  |  |  | Time from arrival to chest radiography for non-admitted patients |  | * |  |  |  | (28) |
| 519 |  |  |  |  | Average ER Radiology Requests Turnaround Time |  | * |  |  |  | (30) |
| 520 |  |  |  |  | Time from arrival to CT |  | * |  |  |  | (28) |
| 521 |  |  |  |  | Time from arrival to brain CT for patients presenting within 4hours of onset of symptoms consistent with a stroke |  | * |  |  |  | (28) |

| **Row** | **Performance aspects** | **Category** | **Sub-category** | **Indicators** | **Sub-indicators** | **Input** | **Process** | **Output** | **Outcome** | **Effect** | **Ref** |
| --- | --- | --- | --- | --- | --- | --- | --- | --- | --- | --- | --- |
| 522 | **Speed** | **Organizational Management** | **Time Management** | **ED Requests Turnaround Time** | Time from arrival to first ECG in suspected cardiac chest pain or acute myocardial infarction |  | * |  |  |  | (28) |
| 523 |  |  |  |  | ECG within 10min of arrival for patients presenting with chest pain |  | * |  |  |  | (28) |
| 524 |  |  |  |  | Time from arrival to reperfusion for patients with acute myocardial infarction |  | * |  |  |  | (28) |
| 525 |  |  |  |  | Average ER Medications Requests Turnaround Time |  | * |  |  |  | (30) |
| 526 |  |  |  |  | Doctor Decision to Patient Admission (Inpatient Boarding Time) |  | * |  |  |  | (30) |
| 527 |  |  |  |  | Door to time to be seen by doctors/nurses in semi-critical (yellow) zone (KPI: maximum 30 min) |  | * |  |  |  | (61) |
| 528 |  |  |  |  | Average Bed to Doctor Time |  | * |  |  |  | (30) |
| 529 |  |  |  |  | Time from arrival to provider |  | * |  |  |  | (28) |
| 530 |  |  |  |  | Time from arrival to pain management |  | * |  |  |  | (28) |
| 531 |  |  |  |  | Time to start of treatment |  | * |  |  |  | (52) |
| 532 |  |  |  |  | Time to treatment condition |  | * |  |  |  | (52) |
| 533 |  |  |  |  | %Patient condition determined in 6 hours |  | * |  |  |  | (76) |
| 534 |  |  |  |  | %Left in 12 hours (%) |  | * |  |  |  | (76) |
| 535 |  |  |  |  | Time from arrival to intravenous tissue plasminogen activator within 4.5 hours of symptom onset in patients with acute ischemic stroke |  | * |  |  |  | (28) |
| 536 |  |  |  |  | ED Throughput (Emergency Department) =ED throughput measures considered median times from ED arrival to ED departure for both admitted and discharged ED patients as well as admit decision time, time to pain management, time to fibrinolytic therapy, and patients left without being seen. |  | * |  |  |  | (24) |
| 537 |  |  |  | **Medical information request time** | Average hours of Internet use |  | * |  |  |  | (64) |
| 538 |  |  |  |  | The average time required for billing |  | * |  |  |  | (64) |
| 539 |  |  |  |  | Response time to customers in archive |  | * |  |  |  | (64) |
| **Row** | **Performance aspects** | **Category** | **Sub-category** | **Indicators** | **Sub-indicators** | **Input** | **Process** | **Output** | **Outcome** | **Effect** | **Ref** |
| 540 | **Speed** | **Organizational Management** | **Time Management** | **Medical information request time** | Time to retrieve an archived file |  | * |  |  |  | (64) |
| 541 |  |  |  | **Medical Equipment Time** | Medical Equipment Delinquent work-orders” (i.e. not completed within 30 days) |  | * |  |  |  | (72) |
| 542 |  |  |  |  | Downtime |  | * |  |  |  | (72) |
| 543 |  |  |  |  | %Medical equipment downtime |  | * |  |  |  | (73) |
| 544 |  |  |  |  | Repair Time |  | * |  |  |  | (72) |
| 545 |  |  |  |  | Uptime |  | * |  |  |  | (72) |
| 546 |  |  |  |  | %Medical equipment uptime |  | * |  |  |  | (73) |
| 547 |  |  |  |  | Response time (in days) |  | * |  |  |  | (73) |
| 548 |  |  |  |  | Mean time to repair = total downtime/number of breakdowns (in days) |  | * |  |  |  | (73) |
| 549 | **Development** | **Organizational Management** | **Training and Education Management** | **staff training number** | Nursing and midwifery group personnel’s participation in training courses outside the hospital |  | * |  |  |  | (81) |
| 550 |  |  |  |  | Paramedical personnel’s participation in training courses outside the hospital |  | * |  |  |  | (81) |
| 551 |  |  |  |  | Administrative personnel’s participation in training courses outside the hospital |  | * |  |  |  | (81) |
| 552 |  |  |  |  | Trained new staff (%) |  | * |  |  |  | (81) |
| 553 |  |  |  |  | Total number of training courses held in a year |  | * |  |  |  | (81) |
| 554 |  |  |  |  | Percentage of passing student in doctors’ training (%) |  | * |  |  |  | (22) |
| 555 |  |  |  |  | Physicians’ participation in training courses outside the hospital |  | * |  |  |  | (81) |
| 556 |  |  |  |  | Frequency per 100 medical workers of training basic medical unit staff |  | * |  |  |  | (21) |
| 557 |  |  |  |  | Frequency per medical worker of further study in upper-level hospitals |  | * |  |  |  | (21) |
| 558 |  |  |  |  | Frequency per medical worker of external short-term training |  | * |  |  |  | (21) |
| 559 |  |  |  | **Hours of training** | Average hours of training =Number of hours of training per year |  | * |  |  |  | (13) |
| **Row** | **Performance aspects** | **Category** | **Sub-category** | **Indicators** | **Sub-indicators** | **Input** | **Process** | **Output** | **Outcome** | **Effect** | **Ref** |
| 560 | **Development** | **Organizational Management** | **Training and Education Management** | **Hours of training** | Number of hours spent on consultation or training |  | * |  |  |  | (18) |
| 561 |  |  |  |  | Total hours of training per year |  | * |  |  |  | (81) |
| 562 |  |  |  |  | Annual CME hours among the housemen/junior residence (KPI: minimum 20 h per rotation) |  | * |  |  |  | (61) |
| 563 |  |  |  |  | Annual continuing medical education (CME) hours among the postgraduate MMed Emergency Medicine (KPI: minimum 50 h annually) |  | * |  |  |  | (61) |
| 564 |  |  |  | **Per capita teaching hours** | Administrative personnel’s per capita teaching hours |  | * |  |  |  | (81) |
| 565 |  |  |  |  | Hospital managers’ per capita teaching hours |  | * |  |  |  | (81) |
| 566 |  |  |  |  | Physicians’ per capita teaching hours |  | * |  |  |  | (81) |
| 567 |  |  |  |  | Nursing and midwifery group personnel’s per capita teaching hours |  | * |  |  |  | (81) |
| 568 |  |  |  |  | Paramedical personnel’s per capita teaching hours |  | * |  |  |  | (81) |
| 569 |  |  |  |  | Continuing education per capita hours |  | * |  |  |  | (81) |
| 570 |  |  |  | **Training courses** | Participation in internal training programs= Annual quality metric, defined as percentage of participation in internal training programs or individual year goals |  | * |  |  |  | (37) |
| 571 |  |  |  |  | Joint training courses with foreign organizations in a year |  | * |  |  |  | (81) |
| 572 |  |  |  |  | Rate of training courses organized based on PDP |  | * |  |  |  | (81) |
| 573 |  |  |  |  | Approved courses held in a year (%) |  | * |  |  |  | (81) |
| 574 |  |  |  |  | Ratio lecturer to undergraduate in EM (KPI: 1:2) |  | * |  |  |  | (61) |
| 575 |  |  |  |  | Ratio lecturer to housemen in EM (KPI: 1:2) |  | * |  |  |  | (61) |
| 576 |  |  |  |  | Percentage of lecturers in ED with subspecialty certification (KPI: minimum 10%) |  | * |  |  |  | (61) |
| 577 |  |  |  |  | Ratio lecturer to trainee in EM (KPI: 1:4) |  | * |  |  |  | (61) |
| 578 |  |  |  |  | Percentage of trained personnel in continuing education courses |  | * |  |  |  | (81) |
| 579 |  |  |  |  | Rate of skill-based training |  | * |  |  |  | (81) |

| **Row** | **Performance aspects** | **Category** | **Sub-category** | **Indicators** | **Sub-indicators** | **Input** | **Process** | **Output** | **Outcome** | **Effect** | **Ref** |
| --- | --- | --- | --- | --- | --- | --- | --- | --- | --- | --- | --- |
| 580 | **Development** | **Organizational Management** | **Training and Education Management** | **Training courses** | % of Junior college education or above in all employees |  | * |  |  |  | (21) |
| 581 |  |  |  |  | Personnel who have a personal development plan (%) |  | * |  |  |  | (81) |
| 582 |  |  |  |  | Employee growth (education and training)= Provide staff with complete medical professional training to improve the medical quality of staff |  | * |  |  |  | (82) |
| 583 |  |  |  |  | Proportion of nurses with basic qualification (%), ≥90% |  | * |  |  |  | (22) |
| 584 |  |  |  |  | Number of Grand rounds and Journal clubs |  | * |  |  |  | (54) |
| 585 |  |  |  |  | Rate of book using by hospital personnel in a year |  | * |  |  |  | (81) |
| 586 |  |  |  |  | Rate of documenting the training of doctors |  | * |  |  |  | (64) |
| 587 |  |  |  | **Student Training** | Number of residents |  | * |  |  |  | (29) |
| 588 |  |  |  |  | Number of precepted residents/students |  | * |  |  |  | (64) |
| 589 |  |  |  |  | Number of nursing students |  | * |  |  |  | (29) |
| 590 |  |  |  |  | Percentage of undergraduate students (MD) satisfied with rotation program in ED (KPI: minimum 80% of all students) |  |  | * |  |  | (61) |
| 591 |  |  |  |  | Percentage of passing student in nurses’ training (%) |  |  | * |  |  | (22) |
| 592 |  |  |  |  | Percentage of postgraduate EM qualify at stipulated time (KPI: minimum 75% of all candidates) |  |  | * |  |  | (61) |
| 593 |  |  |  |  | Percentage of postgraduate EM able to complete dissertation at stipulated time (KPI: minimum 75% of all candidates) |  |  | * |  |  | (61) |
| 594 | **safety** | **Clinical Management** | **Patient Safety** | **Hospital infection rate** | Hospital infection rate |  |  |  | * |  | (8) |
| 595 |  |  |  |  | Hospital-acquired infection rate |  |  |  | * |  | (2) |
| 596 |  |  |  |  | Hospital Acquired Infections (HAIs) (post-surgical) |  |  |  | * |  | (24) |
| 597 |  |  |  |  | Infection rate =Number of infected patients to total of patient treated |  |  |  | * |  | (13) |
| 598 |  |  |  |  | Hospital Infection Rate (HIR)=Percentage of infection that occurs in various departments in a hospital. It is a common measure to determine morbidity rates |  |  |  | * |  | (7) |

| **Row** | **Performance aspects** | **Category** | **Sub-category** | **Indicators** | **Sub-indicators** | **Input** | **Process** | **Output** | **Outcome** | **Effect** | **Ref** |
| --- | --- | --- | --- | --- | --- | --- | --- | --- | --- | --- | --- |
| 599 | **safety** | **Clinical Management** | **Patient Safety** | **Hospital infection rate** | In-hospital sepsis |  |  |  | * |  | (74) |
| 600 |  |  |  | **Surgical Site Infection** | Surgical Site Infection |  |  |  | * |  | (49) |
| 601 |  |  |  |  | Surgical site infection rate |  |  |  | * |  | (20) |
| 602 |  |  |  |  | Surgical site infection (SSI) |  |  |  | * |  | (45) |
| 603 |  |  |  | **ICU infection rate** | ICU infection rate |  |  |  | * |  | (15) |
| 604 |  |  |  |  | Central line associated blood stream infection (CLABSI) rates |  |  |  | * |  | (27) |
| 605 |  |  |  |  | Health care-associated bacterial or fungal infection |  |  |  | * |  | (63) |
| 606 |  |  |  |  | Health care-associated bacterial or fungal infection |  |  |  | * |  | (62) |
| 607 |  |  |  |  | Urinary tract infection (UTI) incidence density |  |  |  | * |  | (60) |
| 608 |  |  |  | **Nosocomial infection** | Nosocomial infection =Nosocomial infection in the hospital |  |  |  | * |  | (16) |
| 609 |  |  |  |  | Incidence of nosocomial infection (%), ≤10% |  |  |  | * |  | (22) |
| 610 |  |  |  |  | Nosocomial infection =Nosocomial infection in the hospital, including surgical wound infection, infection of Methicillin-resistant Staphylococcus aureus (MRSA), incision-wound infection, etc. |  |  |  | * |  | (5) |
| 611 |  |  |  |  | Incidence of nosocomial infection among staff (KPI: 0 incidence) |  |  |  | * |  | (61) |
| 612 |  |  |  | **Other infection** | Methicillin-resistant Staphylococcus aureus (MRSA) |  |  |  | * |  | (45) |
| 613 |  |  |  |  | Vancomycin-resistant Enterococci (VRE) |  |  |  | * |  | (45) |
| 614 |  |  |  |  | Clostridium difficile Infection (CDI) |  |  |  | * |  | (45) |
| 615 |  |  |  |  | Postoperative septicemia rate evaluates cases of sepsis in 100,000 surgical procedures. It is a significant cause of hospital mortality and a significant contributor to health spending in developed countries. However, treatments are not always consistently administered. This indicator assesses the hospital’s capacity to handle these epidemics. |  |  |  | * |  | (17) |
| **Row** | **Performance aspects** | **Category** | **Sub-category** | **Indicators** | **Sub-indicators** | **Input** | **Process** | **Output** | **Outcome** | **Effect** | **Ref** |
| 616 | **safety** | **Clinical Management** | **Patient Safety** | **Other infection** | Percentage of antibacterial prescription (%), 30–45% |  |  |  | * |  | (22) |
| 617 |  |  |  |  | Transfusion transmitted infection (TTI%)= (Combined TTI cases ( HIV + HBV + HCV + Syphilis + Malaria ))/ Total no. of Donors × 100 |  |  |  | * |  | (80) |
| 618 |  |  |  |  | Prevention of health care related infections: Research on infections related to invasive devices and multi-resistance for clinical epidemiology |  |  |  | * |  | (60) |
| 619 |  |  |  | **Accidents/Adverse Events** | Accidents/Adverse Events= Accident/adverse events occurred in healthcare treatment process, e.g., number of critical incidents per 100 operations |  | * |  |  |  | (5) |
| 620 |  |  |  |  | Accidents/Adverse events =Accident/adverse events occurring in healthcare treatment process |  | * |  |  |  | (16) |
| 621 |  |  |  |  | Anesthetic Adverse Outcome |  | * |  |  |  | (49) |
| 622 |  |  |  |  | Medical nurse-sensitive adverse events (in%) |  | * |  |  |  | (59) |
| 623 |  |  |  |  | Risk management= Adverse events registering routine |  | * |  |  |  | (60) |
| 624 |  |  |  |  | Major adverse event rate=It represented the rate of specific complications following an emergency general surgery |  | * |  |  |  | (53) |
| 625 |  |  |  |  | Number of adverse drug reaction reported |  | * |  |  |  | (54) |
| 626 |  |  |  |  | Adverse drug reaction numbers |  | * |  |  |  | (2) |
| 627 |  |  |  |  | Accidental cut, puncture, or hemorrhage during medical care |  | * |  |  |  | (46) |
| 628 |  |  |  |  | Medical accident rate per 10,000 inpatients |  | * |  |  |  | (22) |
| 629 |  |  |  |  | Operating Room accident rate |  | * |  |  |  | (20) |
| 630 |  |  |  |  | Adverse Transfusion Reaction Rate (ATRR) %= No. of adverse transfusion reactions /Total no. of blood and components issued × 100 |  | * |  |  |  | (80) |
| 631 |  |  |  |  | Adverse Donor Reaction Rate % =No. of donors experiencing adverse reaction/Total no. of donors × 100 |  | * |  |  |  | (80) |
| 632 |  |  |  | **Incidents/Errors rate** | Medical errors |  | * |  |  |  | (52) |
| 633 |  |  |  |  | Incidents/Errors=Incidents/errors occurred in healthcare treatment process, including medication errors, diagnosis, etc. |  | * |  |  |  | (16) |
| **Row** | **Performance aspects** | **Category** | **Sub-category** | **Indicators** | **Sub-indicators** | **Input** | **Process** | **Output** | **Outcome** | **Effect** | **Ref** |
| 634 | **safety** | **Clinical Management** | **Patient Safety** | **Incidents/Errors rate** | Incidents/Errors =Incidents/errors occurred in healthcare treatment process, including medication errors, diagnosis and treatment errors, blood transfusion errors, etc. |  | * |  |  |  | (5) |
| 635 |  |  |  |  | Number of medication errors reported |  | * |  |  |  | (64) |
| 636 |  |  |  |  | Error rate in exam result reports |  | * |  |  |  | (32) |
| 637 |  |  |  |  | Clinical errors |  | * |  |  |  | (8) |
| 638 |  |  |  |  | Errors rate= Number of errors to number of patients treated, errors includes coding errors ,medication errors |  | * |  |  |  | (13) |
| 639 |  |  |  |  | Incident report numbers |  | * |  |  |  | (2) |
| 640 |  |  |  |  | Incidence of needle prick injury in department (KPI: 0 incidence annually) |  | * |  |  |  | (61) |
| 641 |  |  |  |  | ICU Needle Stick |  | * |  |  |  | (15) |
| 642 |  |  |  |  | ICU Injury Rate |  | * |  |  |  | (15) |
| 643 |  |  |  |  | ICU Patient Fall Rate |  | * |  |  |  | (15) |
| 644 |  |  |  |  | Ventilator-associated pneumonia (VAP) incidence density |  | * |  |  |  | (60) |
| 645 |  |  |  |  | Incidents of participation in resolving and troubleshooting medication supply issues |  | * |  |  |  | (64) |
| 646 |  |  |  |  | Central-line associated bloodstream (CLABSI) incidence density |  | * |  |  |  | (60) |
| 647 |  |  |  | **Complications** | Complications |  | * |  |  |  | (24) |
| 648 |  |  |  |  | ICU Complication |  | * |  |  |  | (15) |
| 649 |  |  |  |  | Postoperative complications rate =Half yearly or annual average patients’ postoperative complications rate compared to the country or region’s normative average rate for the ward’s specialty |  | * |  |  |  | (37) |
| 650 |  |  |  |  | Surgical schedule compliance rate |  | * |  |  |  | (20) |

| **Row** | **Performance aspects** | **Category** | **Sub-category** | **Indicators** | **Sub-indicators** | **Input** | **Process** | **Output** | **Outcome** | **Effect** | **Ref** |
| --- | --- | --- | --- | --- | --- | --- | --- | --- | --- | --- | --- |
| 651 | **safety** | **Clinical Management** | **Patient Safety** | **Complications** | Postoperative pulmonary embolism/ deep vein thrombosis rate evaluates cases of pulmonary embolism/ deep vein thrombosis in 100,000 surgical procedures. It is the third leading cause of hospital death, although it is the most preventable Hence, this indicator reveals the hospital entity’s capacity to deal with these episodes, namely, in the pulmonary embolism/ deep vein thrombosis prophylaxis. |  | * |  |  |  | (17) |
| 652 |  |  |  |  | Pulmonary embolism |  | * |  |  |  | (46) |
| 653 |  |  |  |  | Deep vein thrombosis |  | * |  |  |  | (46) |
| 654 |  |  |  |  | Percentage of CVI patients with anticoagulant therapy administrated (%anticoag-ther-cvi) |  | * |  |  |  | (38) |
| 655 |  |  |  |  | Percentage of CVI patients with clinical state index assessed (%clinical-ix-cvi) |  | * |  |  |  | (38) |
| 656 |  |  |  |  | Percentage of CVI patients with admission time recorded in the medical record (%admission-time-cvi) |  | * |  |  |  | (38) |
| 657 |  |  |  |  | Door to needle for thrombolysis in CVA (KPI: within 90 min of arrival) |  | * |  |  |  | (61) |
| 658 |  |  |  |  | Percentage of AMI patients with comorbidity index assessed (%comorb-ix-ami) |  | * |  |  |  | (38) |
| 659 |  |  |  |  | Rate of hospital days per patient with acute myocardial infarction (AMI) |  | * |  |  |  | (39) |
| 660 |  |  |  |  | Percentage of AMI patients discharged from the hospital to a rehabilitation facility (%rehabilitation-ami) |  | * |  |  |  | (38) |
| 661 |  |  |  |  | Percentage of AMI patients with admission time recorded in the medical record (%admission-time-ami) |  | * |  |  |  | (38) |
| 662 |  |  |  |  | Heart attack patients given PCI within 90 minutes of arrival (AMI-8a) |  | * |  |  |  | (69) |
| 663 |  |  |  |  | Heart attack patients given fibrinolytic agent within 30 minutes of arrival (AMI-7a) |  | * |  |  |  | (69) |
| 664 |  |  |  |  | Door to thrombolytic for AMI (KPI: within 30 min of arrival) |  | * |  |  |  | (61) |
| 665 |  |  |  |  | Rate of coronary intervention for acute myocardial infarction |  | * |  |  |  | (2) |
| 666 |  |  |  |  | AMI= MACCE after 1 year |  | * |  |  |  | (42) |
| **Row** | **Performance aspects** | **Category** | **Sub-category** | **Indicators** | **Sub-indicators** | **Input** | **Process** | **Output** | **Outcome** | **Effect** | **Ref** |
| 667 | **safety** | **Clinical Management** | **Patient Safety** | **Complications** | Surgery patients who were given an antibiotic at the right time (within one hour before surgery) to help prevent infection (SCIP-INF-1) |  | * |  |  |  | (69) |
| 668 |  |  |  |  | Surgery patients who were given the right kind of antibiotic to help prevent infection (SCIP-INF-2) |  | * |  |  |  | (69) |
| 669 |  |  |  |  | Surgery patients whose preventive antibiotics were stopped at the right time (SCIP-INF-3) |  | * |  |  |  | (69) |
| 670 |  |  |  |  | Heart surgery patients whose blood sugar (blood glucose) is kept under good control in the days right after surgery (SCIP-INF-4) |  | * |  |  |  | (69) |
| 671 |  |  |  |  | Surgery patients whose urinary catheters were removed on the first or second day after surgery (SCIP-INF-9) |  | * |  |  |  | (69) |
| 672 |  |  |  |  | Surgery patients on beta blockers before coming to the hospital who were kept on the beta blockers during the perioperative period (SCIP-CARD-2) |  | * |  |  |  | (69) |
| 673 |  |  |  |  | Surgery patients whose doctors ordered treatments to prevent blood clots after certain types of surgeries (SCIP-VTE-1) |  | * |  |  |  | (69) |
| 674 |  |  |  |  | Pneumonia patients whose initial emergency room blood culture was performed prior to the administration of the first hospital dose of antibiotics (PN-3b) |  | * |  |  |  | (69) |
| 675 |  |  |  |  | Pneumonia patients given the most appropriate initial antibiotic (PN-6) |  | * |  |  |  | (69) |
| 676 |  |  |  |  | Obstetric trauma |  | * |  |  |  | (46) |
| 677 |  |  |  |  | Obstetric trauma (with instrument) |  | * |  |  |  | (74) |
| 678 |  |  |  |  | Trauma on vaginal delivery (instrumented and non-instrumented) with lacerations of third and fourth degree. This indicator assesses the obstetric care quality in hospitals. Patient safety during delivery is assessed through potentially perineum preventable lacerations. Lacerations are not always preventable, but they can be reduced through quality obstetric care. |  | * |  |  |  | (17) |
| 679 |  |  |  | **Surgical safety checklist** | Surgical safety checklist |  | * |  |  |  | (45) |
| 680 |  |  |  |  | Rate of Safe Surgery Checklist (SSC) Utilization |  | * |  |  |  | (49) |
| **Row** | **Performance aspects** | **Category** | **Sub-category** | **Indicators** | **Sub-indicators** | **Input** | **Process** | **Output** | **Outcome** | **Effect** | **Ref** |
| 681 | **safety** | **Clinical Management** | **Patient Safety** | **Surgical safety checklist** | Rate of Surgical Safety Checklist Utilization (Any Method) |  | * |  |  |  | (83) |
| 682 |  |  |  |  | Rate of Surgical Safety Checklist Utilization (On Paper, Read Aloud) |  | * |  |  |  | (83) |
| 683 |  |  |  |  | Patient Identity, Consent, and Procedure Confirmation Rate |  | * |  |  |  | (83) |
| 684 |  |  |  | **Surgical safety measures** | Pulse Oximetry Usage Rate |  | * |  |  |  | (83) |
| 685 |  |  |  |  | Rate of Surgical Instruments Free of Visible Soil |  | * |  |  |  | (83) |
| 686 |  |  |  |  | Rate of Observed Chemical Sterilization Tape |  | * |  |  |  | (83) |
| 687 |  |  |  |  | Rate of Appropriate Operative Site Cleansing |  | * |  |  |  | (83) |
| 688 |  |  |  |  | Rate of Vaginal Cleansing with Povidone-Iodine (for caesarean sections only) |  | * |  |  |  | (83) |
| 689 |  |  |  |  | Instrument, Sponge, and Needle Count Verifications |  | * |  |  |  | (83) |
| 690 |  |  |  |  | Rate of Post-op Decontamination of All (used and unused) Instruments |  | * |  |  |  | (83) |
| 691 |  |  |  |  | Appropriate Prophylactic Antibiotic Administration Rate |  | * |  |  |  | (83) |
| 692 |  |  |  |  | Surgery, Anesthesia, Obstetrics (SAO) Provider Density |  | * |  |  |  | (49) |
| 693 |  |  |  |  | Surgical Scheduling Adequacy values (SA1 and SA2) (by year for major/minor/total surgery) |  | * |  |  |  | (44) |
| 694 |  |  |  |  | Femur fracture= percentage of operations carried out within 2 days |  | * |  |  |  | (42) |
| 695 |  |  |  |  | Hip fracture surgery in the first 48 h. Hip fractures represent a significant mortality cause, mainly if they occur in elderly users. Postoperative complications have a high incidence. Although there is no consensus on the surgery’s ideal waiting time, the procedure should be carried out in the first 48 h after admission. In this study, this variable is considered in the access group, as it assesses the opportune time for orthopedic surgeons to deal with this type of case. |  | * |  |  |  | (17) |
| 696 |  |  |  | **ED safety measures** | Brain imaging in stroke suspicious patients |  | * |  |  |  | (28) |

| **Row** | **Performance aspects** | **Category** | **Sub-category** | **Indicators** | **Sub-indicators** | **Input** | **Process** | **Output** | **Outcome** | **Effect** | **Ref** |
| --- | --- | --- | --- | --- | --- | --- | --- | --- | --- | --- | --- |
| 697 | **safety** | **Clinical Management** | **Patient Safety** | **ED safety measures** | Determination of the respiratory rate at admission for patients with outpatient-acquired pneumonia |  | * |  |  |  | (28) |
| 698 |  |  |  |  | Patient care compromised |  | * |  |  |  | (52) |
| 699 |  |  |  |  | Patient transfers |  | * |  |  |  | (52) |
| 700 |  |  |  |  | Increase diagnostic test |  | * |  |  |  | (52) |
| 701 |  |  |  |  | Missed diagnoses in ED rate=highlighted disparities of missed diagnoses of acute myocardial infarction according to insurance status or median household income |  | * |  |  |  | (53) |
| 702 |  |  |  |  | Ambulatory care sensitive conditions (ACSCs)x ED visits/ACSC emergency admission rate ((Also called Preventable ED visits/Preventable emergency admissions)) |  | * |  |  |  | (53) |
| 703 |  |  |  |  | Amount of Personal Protective Equipment provided and supplied annually (KPI: adequate to fulfil the use requirement) |  | * |  |  |  | (61) |
| 704 |  |  |  |  | Hand hygiene practice among staff (KPI: 100% compliance) |  | * |  |  |  | (61) |
| 705 |  |  |  |  | ECG for patients with non-traumatic chest pain |  | * |  |  |  | (28) |
| 706 |  |  |  |  | ECG performed for syncope |  | * |  |  |  | (28) |
| 707 |  |  |  | **ICU safety measures** | ICU Pressure Sore Rate |  | * |  |  |  | (15) |
| 708 |  |  |  |  | Mechanical ventilation (MV) utilization rate |  | * |  |  |  | (60) |
| 709 |  |  |  |  | Duration Of Mechanical Ventilation |  | * |  |  |  | (15) |
| 710 |  |  |  |  | Standardized resource use (SRU)= SRU corresponds to the observed resource use to the expected resource use ratio. For this purpose, we considered the ICU LOS as a surrogate measure of ICU resource use. Following the SRU methodology, the observed use of resources was calculated as the total ICU LOS and the expected use of resources was the average ICU LOS per surviving patient. |  | * |  |  |  | (75) |
| 711 |  |  |  |  | Central venous catheter (CVC) utilization rate |  | * |  |  |  | (60) |
| 712 |  |  |  |  | Number Of Unplanned Extubation |  | * |  |  |  | (15) |
| **Row** | **Performance aspects** | **Category** | **Sub-category** | **Indicators** | **Sub-indicators** | **Input** | **Process** | **Output** | **Outcome** | **Effect** | **Ref** |
| 713 | **safety** | **Clinical Management** | **Patient Safety** | **ICU safety measures** | APACHEII score – SOFA score-Upper Body Elevation- Stress Ulcer Prophylaxis -Therapeutic Hypothermia-GCS On Admission- RASS Score- Blood Glucose- TV- bpsVAS/NRS-Map-Pip |  | * |  |  |  | (15) |
| 714 |  |  |  |  | Transport of patients: Transportation of patient with adequate equipment |  | * |  |  |  | (60) |
| 715 |  |  |  |  | Number Of Clinical Transport |  | * |  |  |  | (15) |
| 716 |  |  |  |  | Intensivist Cover 24/7 |  | * |  |  |  | (15) |
| 717 |  |  |  |  | Return to the intensive care unit within 48 h |  | * |  |  |  | (2) |
| 718 |  |  |  |  | Clostridium difficile (C. diff.) colitis rates |  | * |  |  |  | (27) |
| 719 |  |  |  |  | APACHEII score – SOFA score-Upper Body Elevation- Stress Ulcer Prophylaxis -Therapeutic Hypothermia-GCS On Admission- RASS Score- Blood Glucose- TV- bpsVAS/NRS-Map-Pip |  | * |  |  |  | (27) |
| 720 |  |  |  | **Safety standards in the archives** | Percent of safety standards in the archives |  | * |  |  |  | (64) |
| 721 |  |  |  | **Nutrition Risk Screening** | Nutrition Risk Screening |  | * |  |  |  | (33) |
| 722 |  |  |  | **Other safety measures** | Pressure ulcers |  | * |  |  |  | (45) |
| 723 |  |  |  |  | Patients who got treatment at the right time to help prevent blood clots after certain types of surgery (SCIP-VTE-2) |  |  |  | * |  | (69) |
| 724 |  |  |  |  | Staff always explained new medications |  |  |  | * |  | (69) |
| 725 |  |  |  |  | Rate of thrombolytic therapy for acute ischemic stroke patients |  |  |  | * |  | (2) |
| 726 |  |  |  |  | Patient safety=Provide trusty services and keep patients from errors, infections, and delays |  |  |  | * |  | (40) |
| 727 | **Quality of work life** | **Organizational Management** | **Human Recourse Management** | **Staff turnover (staff leaving, transfer, ...)** | Staff turnover =Staff turnover of the hospital, e.g., staff leaving the hospital in one year/total staffs |  | * |  |  |  | (5) |
| **Row** | **Performance aspects** | **Category** | **Sub-category** | **Indicators** | **Sub-indicators** | **Input** | **Process** | **Output** | **Outcome** | **Effect** | **Ref** |
| 728 | **Quality of work life** | **Organizational Management** | **Human Recourse Management** | **Staff turnover (staff leaving, transfer, ...)** | Employee turnover=The turnover rate of employees leaving the hospital |  | * |  |  |  | (16) |
| 729 |  |  |  |  | Staff turnover |  | * |  |  |  | (8) |
| 730 |  |  |  |  | Staff turnover rate=Staff leaving the hospital in one year/total staffs |  | * |  |  |  | (13) |
| 731 |  |  |  | **Staff workload** | Workload of the staff |  | * |  |  |  | (32) |
| 732 |  |  |  |  | Employee fatigue in ED |  | * |  |  |  | (52) |
| 733 |  |  |  |  | Physician burden of medical treatment per day |  |  |  | * |  | (21) |
| 734 |  |  |  |  | Physician burden of hospitalization duration per day |  |  |  | * |  | (21) |
| 735 |  |  |  | **Staff work condition (absenteeism, sickness leave, …)** | Employee absenteeism rate |  | * |  |  |  | (8) |
| 736 |  |  |  |  | Sickness leave =Employee sick leave in the hospital, such as sickness leave of doctor, mental sickness, physical sickness |  | * |  |  |  | (5) |
| 737 |  |  |  |  | Availability of staff |  | * |  |  |  | (59) |
| 738 |  |  |  |  | Frequency per 100 medical workers of providing counterpart assistance to basic medical units |  | * |  |  |  | (21) |
| 739 |  |  |  |  | Frequency per 100 medical workers of undertaking sudden public health events and emergency medical rescue |  | * |  |  |  | (21) |
| 740 |  |  |  |  | Average year of experience at work for staff |  | * |  |  |  | (2) |
| 741 |  |  |  | **Staff/Physicians working hours** | Number of working hours for specialist doctors |  | * |  |  |  | (18) |
| 742 |  |  |  |  | Number of working hours for supporting medical personnel (nurses, therapists, etc.) |  | * |  |  |  | (18) |
| 743 |  |  |  |  | Average nursing time in days |  | * |  |  |  | (9) |
| 744 |  |  |  |  | Number of completed work orders per staff |  | * |  |  |  | (65) |
| 745 |  |  |  |  | Resource hours= Number of hours that the OR has planned staffing. Includes block time and open time but not overrun time. The current upper limit is 37.5 hours per week for one OR |  | * |  |  |  | (48) |

| **Row** | **Performance aspects** | **Category** | **Sub-category** | **Indicators** | **Sub-indicators** | **Input** | **Process** | **Output** | **Outcome** | **Effect** | **Ref** |
| --- | --- | --- | --- | --- | --- | --- | --- | --- | --- | --- | --- |
| 746 | **Quality** | **Organizational Management** | **Quality Improvement** | **ED Left against medical advice** | Percentage of Leaving Before Screening |  |  |  | * |  | (30) |
| 747 |  |  |  |  | Percentage of Leaving Without Being Seen |  |  |  | * |  | (30) |
| 748 |  |  |  |  | Left against medical advice |  |  |  | * |  | (28) |
| 749 |  |  |  |  | Left before treatment completion |  |  |  | * |  | (28) |
| 750 |  |  |  |  | %Leaving hospital with the personal responsibility |  |  |  | * |  | (76) |
| 751 |  |  |  |  | Percentage of Patients Leaving Before Complete Treatment |  | * |  |  |  | (30) |
| 752 |  |  |  |  | Percentage of patients leaving ED against/without medical advice= Proxy of patient satisfaction on ED services and waiting times. |  | * |  |  |  | (42) |
| 753 |  |  |  |  | Percentage hospitalized patients leaving against medical advice= Proxy of patient satisfaction for the inpatient activity. |  |  |  | * |  | (42) |
| 754 |  |  |  |  | Left before/without being seen |  |  |  | * |  | (28) |
| 755 |  |  |  | **Lab QC failures** | number of analyses rejected as non-compliant over a given period |  |  |  | * |  | (32) |
| 756 |  |  |  |  | number of analyses rejected as non-compliant over a given period, |  |  |  | * |  | (32) |
| 757 |  |  |  |  | Rate of analyses rejected as non-compliant over a given period |  |  |  | * |  | (32) |
| 758 |  |  |  |  | False diagnostic rate |  |  |  | * |  | (32) |
| 759 |  |  |  |  | Irradiation dose |  |  |  | * |  | (32) |
| 760 |  |  |  |  | Rate of unread reports |  |  |  | * |  | (32) |
| 761 |  |  |  |  | Rate of lost reports |  |  |  | * |  | (32) |
| 762 |  |  |  |  | Delay in transfusion beyond 30 min after issue |  |  |  | * |  | (80) |
| 763 |  |  |  |  | Wastage rate %: (excluding discards due to TTI reactivity)= (No. of blood∕ blood components discarded)/(Total no of blood ∕ blood components issued) × 100 |  |  |  | * |  | (80) |

| **Row** | **Performance aspects** | **Category** | **Sub-category** | **Indicators** | **Sub-indicators** | **Input** | **Process** | **Output** | **Outcome** | **Effect** | **Ref** |
| --- | --- | --- | --- | --- | --- | --- | --- | --- | --- | --- | --- |
| 764 | **Quality** | **Organizational Management** | **Quality Improvement** | **Lab QC failures** | Component QC failures (for each component)= No. of component QC failures/Total no. of component tested× 100 |  |  |  | * |  | (80) |
| 765 |  |  |  |  | Donor Deferral Rate %= No. of donor deferrals/(Total no. of donation + total no. of deferrals)× 100 |  |  |  | * |  | (80) |
| 766 |  |  |  |  | Blood Unavailability Ratio in OR(operating room) |  |  |  | * |  | (49) |
| 767 |  |  |  |  | Percentage (%) of components =Total component issues/(Total whole blood + component issues)× 100 |  |  |  | * |  | (80) |
| 768 |  |  |  |  | TTI outliers % age = No. of deviations beyond ± 2SD × 100 |  |  |  | * |  | (80) |
| 769 |  |  |  |  | Quality of the exam request filling |  |  |  | * |  | (32) |
| 770 |  |  |  |  | Proportion of samples that cannot be analyzed |  |  |  | * |  | (32) |
| 771 |  |  |  | **Imaging QC failures** | number of redundant exams per hospital stay |  |  |  | * |  | (32) |
| 772 |  |  |  |  | Number of redundant exams per hospital stay |  |  |  | * |  | (32) |
| 773 |  |  |  |  | rate of exams requests modified by radiologists |  |  |  | * |  | (32) |
| 774 |  |  |  |  | Proportion of lost exams in patients files Absorbed radiation dose per patient |  |  |  | * |  | (32) |
| 775 |  |  |  |  | Proportion of exams requests modified by radiologists |  |  |  | * |  | (32) |
| 776 |  |  |  |  | Proportion of images viewed by requesting physicians |  |  |  | * |  | (32) |
| 777 |  |  |  |  | Proportion of reports read by requesting physicians |  |  |  | * |  | (32) |
| 778 |  |  |  | **Error in Medical report** | Percent error in the coding |  |  |  | * |  | (64) |
| 779 |  |  |  |  | The average errors in the statistics report |  |  |  | * |  | (64) |
| 780 |  |  |  | **Medical Equipment**  **QC failure** | Class failure rate |  |  |  | * |  | (72) |
| 781 |  |  | **Quality Improvement** |  | Age failure rate |  |  |  | * |  | (72) |
| 782 |  |  |  |  | Percentage of SM with problems |  |  |  | * |  | (64) |
| 783 |  | **Clinical Management** | **Patient Safety** | **Compliance rate** | Laboratory Compliance rate with guidelines |  | * |  |  |  | (32) |

| **Row** | **Performance aspects** | **Category** | **Sub-category** | **Indicators** | **Sub-indicators** | **Input** | **Process** | **Output** | **Outcome** | **Effect** | **Ref** |
| --- | --- | --- | --- | --- | --- | --- | --- | --- | --- | --- | --- |
| 784 | **Quality** | **Clinical Management** | **Patient Safety** | **Compliance Rate** | Imaging compliance rate of exam requests with guidelines |  | * |  |  |  | (32) |
| 785 |  |  |  |  | Imaging Compliance rate of exam requests with guidelines |  | * |  |  |  | (32) |
| 786 |  |  |  |  | Imaging Compliance rate of exam requests with guidelines |  | * |  |  |  | (32) |
| 787 |  |  |  |  | Imaging compliance rate of exam requests with guidelines |  | * |  |  |  | (32) |
| 788 |  |  |  |  | Imaging Compliance rate of exam requests with guidelines |  | * |  |  |  | (32) |
| 789 |  |  |  |  | Percentage of medical inpatient discharges within 2 days (National Healthcare Agreement 2010)= Measure of hospital compliance in avoiding short ordinary hospitalizations for patients who could be treated in outpatient clinics or in other care settings |  | * |  |  |  | (42) |
| 790 |  | **Organizational Management** |  |  | Percentage of day case surgery for specific procedures (National Healthcare Agreement 2010)= Measure of hospital compliance with Italian Ministry of Health standards for delivering specific, not complex, surgical procedures in day case surgery or in outpatient clinics rather than through ordinary hospitalizations. |  | * |  |  |  | (42) |
| 791 |  |  |  |  | Standardized day surgery rate= This is calculated as the ratio of the ‘actual’ to ‘expected’ day surgery rate for each hospital, multiplied by the overall day surgery rate for the 35 hospitals, expressed as a percentage. This ratio represents, for each hospital, the day surgery rate adjusted for the DRG-mix of surgeries in each hospital |  | * |  |  |  | (41) |
| 792 |  | **Clinical Management** |  | **Pain Management** | Pain management |  | * |  |  |  | (59) |
| 793 |  |  |  |  | Pain was always well controlled |  | * |  |  |  | (69) |
| 794 | **Satisfaction** | **Organizational Management** | **Human Recourse Management** | **Staff Satisfaction rate** | Staff satisfaction rate |  |  |  | * |  | (8) |
| 795 |  |  |  |  | Staff satisfaction rate= Number of staffs expressed ""satisfaction"" in surveys/total surveyed staff |  |  |  | * |  | (13) |
| 796 |  |  |  |  | Staff satisfaction =Number of staffs expressed “satisfaction” |  |  |  | * |  | (16) |
| **Row** | **Performance aspects** | **Category** | **Sub-category** | **Indicators** | **Sub-indicators** | **Input** | **Process** | **Output** | **Outcome** | **Effect** | **Ref** |
| 797 | **Satisfaction** | **Organizational Management** | **Human Recourse Management** | **Staff Satisfaction rate** | Employee satisfaction |  |  |  | * |  | (52) |
| 798 |  |  |  |  | Satisfaction of the staff, feeling of efficiency |  |  |  | * |  | (32) |
| 799 |  |  |  |  | Satisfaction of the staff, feeling of efficiency |  |  |  | * |  | (32) |
| 800 |  |  |  |  | Employee satisfaction=Satisfaction of staff |  |  |  | * |  | (40) |
| 801 |  |  |  |  | Overall satisfaction = Overall satisfaction with healthcare organization, including satisfaction with job, colleagues, supervisors etc. |  |  |  | * |  | (5) |
| 802 |  | **Clinical Management** | **Service Recipients rights** | **Patient Satisfaction** | Patient Satisfaction |  |  |  | * |  | (84) |
| 803 |  |  |  |  | Patients’ Satisfaction= The most accurately it is defined by Eriksen: “Patients’ satisfaction is the assessment of patients’ subjective, cognitive and emotional response that results from interaction between expectations about nursing and actual attitude of nurses.” |  |  |  | * |  | (85) |
| 804 |  |  |  |  | HCAPHS Surveys (Hospital Consumer Assessment of Healthcare Providers and Systems) |  |  |  | * |  | (24) |
| 805 |  |  |  |  | Patient satisfaction rate (C13) =Number of patients expressed ""satisfaction"" in surveys/total surveyed patients |  |  |  | * |  | (13) |
| 806 |  |  |  |  | Discharge with personal satisfaction |  |  |  | * |  | (8) |
| 807 |  |  |  |  | Inpatient satisfaction |  |  |  | * |  | (21) |
| 808 |  |  |  |  | Outpatient satisfaction |  |  |  | * |  | (21) |
| 809 |  |  |  |  | Satisfaction of the patients |  |  |  | * |  | (2) |
| 810 |  |  |  |  | Patients satisfaction percentage |  |  |  | * |  | (8) |
| 811 |  |  |  |  | Patient Satisfaction |  |  |  | * |  | (49) |
| 812 |  |  |  |  | Patient satisfaction level =Half yearly or annual quality metric defined and digitalized through patient surveys. |  |  |  | * |  | (37) |
| 813 |  |  |  |  | Patient satisfaction=Patients’ expectations of all services, products, transportation, and inventory |  |  |  | * |  | (40) |
| 814 |  |  |  |  | Statistics satisfaction |  |  |  | * |  | (64) |
| 815 |  |  |  |  | Patient Satisfaction |  |  |  | * |  | (58) |
| **Row** | **Performance aspects** | **Category** | **Sub-category** | **Indicators** | **Sub-indicators** | **Input** | **Process** | **Output** | **Outcome** | **Effect** | **Ref** |
| 816 | **Satisfaction** | **Clinical Management** | **Service Recipients rights** | **Patient Satisfaction** | Patient satisfaction (%) |  |  |  | * |  | (22) |
| 817 |  |  |  |  | Admission satisfaction |  |  |  | * |  | (64) |
| 818 |  |  |  |  | Overall satisfaction (P1) =Overall satisfaction with healthcare service, including satisfaction with physicians, waiting time, treatment, etc. |  |  |  | * |  | (5) |
| 819 |  |  |  |  | Overall satisfaction (F1)= Satisfaction with healthcare service, including satisfaction with physicians, wait time etc. |  |  |  | * |  | (16) |
| 820 |  |  |  |  | % of patients satisfied with the information they received about their medications while in hospital |  |  |  | * |  | (86) |
| 821 |  |  |  | **Rate of complaints** | Overall complaint=Number of patients expressing complaint |  |  |  | * |  | (16) |
| 822 |  |  |  |  | Patient complaint rate =Number of patients expressing complaint in the information system |  |  |  | * |  | (13) |
| 823 |  |  |  |  | Overall complaints=Overall complaints about healthcare service, e.g., patient complaints per 1000 patient, rate of complaints per patient per year |  |  |  | * |  | (5) |
| 824 |  |  |  |  | Rate of patient complaints |  |  |  | * |  | (8) |
| 825 |  |  |  |  | Number of complaints and complications filed |  |  |  | * |  | (18) |
| 826 |  |  |  |  | The rate of complaint in archive |  |  |  | * |  | (64) |
| 827 | **Innovation** | **Organizational Management** | **Research Assessment** | **Number of publications** | Number of scientific publications |  |  |  | * |  | (29) |
| 828 |  |  |  |  | Average number of publications per physician |  |  |  | * |  | (87) |
| 829 |  |  |  |  | Academic paper=Number of academic paper per year |  |  |  | * |  | (13) |
| 830 |  |  |  |  | Nursing and midwifery group personnel’s scientific papers per capita |  |  |  | * |  | (81) |
| 831 |  |  |  |  | Paramedical personnel scientific papers per capita |  |  |  | * |  | (81) |
| 832 |  |  |  |  | Administrative personnel’s scientific papers per capita |  |  |  | * |  | (81) |
| 833 |  |  |  |  | Physicians’ scientific papers per capita |  |  |  | * |  | (81) |
| 834 |  |  |  | **Average impact factor** | Average impact factor per physician |  |  |  | * |  | (87) |
| **Row** | **Performance aspects** | **Category** | **Sub-category** | **Indicators** | **Sub-indicators** | **Input** | **Process** | **Output** | **Outcome** | **Effect** | **Ref** |
| 835 |  | **Organizational Management** | **Research Assessment** | **Average impact factor** | Median impact factor per specialty |  |  |  | * |  | (87) |
| 836 | **Innovation** |  |  |  | Median impact factor variation per specialty |  |  |  | * |  | (87) |
| 837 |  |  |  |  | Percentage of publications with an average impact factor higher than the benchmark specialty impact factor reported in ISI |  |  |  | * |  | (87) |
| 838 |  |  |  |  | Percentage of publications with a median impact factor higher than the benchmark specialty impact factor reported in ISI |  |  |  | * |  | (87) |
| 839 |  |  |  | **Participation in scientific events** | Physicians’ participation in scientific conferences |  |  | * |  |  | (81) |
| 840 |  |  |  |  | Nursing and midwifery group personnel’s participation in scientific conferences |  |  | * |  |  | (81) |
| 841 |  |  |  |  | Paramedical personnel participation in scientific conferences |  |  | * |  |  | (81) |
| 842 |  |  |  |  | Administrative personnel’s participation in scientific conferences |  |  | * |  |  | (81) |
| 843 |  |  |  |  | Percentage of lecturers in ED hold research grant at any time (KPI: minimum 10%) |  |  | * |  |  | (61) |
| 844 | **Appropriateness** | **Clinical Management** | **Service delivery and Treatment** | **Treatment Rate** | Cure rate |  |  |  | * |  | (21) |
| 845 |  |  |  |  | Improvement rate |  |  |  | * |  | (21) |
| 846 |  |  |  |  | Success rate of rescue (%) |  |  |  | * |  | (22) |
| 847 |  |  |  |  | Therapeutic response rate (%) |  |  |  | * |  | (22) |
| 848 |  |  |  |  | No. of treatment days= Total number of days that patients were treated |  |  | * |  |  | (51) |
| 849 |  |  |  |  | Proportion of inpatients diagnosed within 3 days (%) |  |  |  | * |  | (22) |
| 850 |  |  |  |  | Coincidence rate of admission and clinic diagnosis |  |  |  | * |  | (21) |
| 851 |  |  |  |  | Coincidence rate of admission and discharge diagnosis |  |  |  | * |  | (21) |
| 852 |  |  |  |  | Percentage of agreement between admission and discharge diagnoses (%), ≥95% |  |  | * |  |  | (22) |

| **Row** | **Performance aspects** | **Category** | **Sub-category** | **Indicators** | **Sub-indicators** | **Input** | **Process** | **Output** | **Outcome** | **Effect** | **Ref** |
| --- | --- | --- | --- | --- | --- | --- | --- | --- | --- | --- | --- |
| 853 | **Appropriateness** | **Clinical Management** | **Service delivery and Treatment** | **Treatment Rate** | Number of medical disputes per 1000 discharged patients |  |  |  | * |  | (21) |
| 854 |  |  |  |  | Percentage of medical discharges with LOS over the threshold for patients aged 65 and over= Measure of the hospital compliance with the Italian Ministry of Health standards for the LOS for medical inpatient activity for elderly patients. This measure is a proxy for the effective implementation of integrated pathways between home, community-based and hospital care for elderly patients |  |  |  | * |  | (42) |
| 855 |  |  |  |  | Increase ED treatment |  |  |  | * |  | (52) |
| 856 |  |  |  |  | Emergency-specific procedure rate=This indicator comprised a combination of different procedures performed during emergency care, highlighting disparities in the quality or access to care for specific emergency conditions such as a brain scan for the diagnosis of acute stroke, reperfusion therapy in acute stroke, and cardiac catheterization after myocardial infarction or cardiac arrest. |  |  |  | * |  | (53) |
| 857 |  |  |  |  | Neurological recovery rate=analyzing the neurological recovery over time of patients who presented to the ED with a cardiac arrest. |  |  |  | * |  | (53) |
| 858 |  |  |  |  | Delay to diagnosis or treatment rate=disparities in time to access to a diagnostic, or therapeutic procedure. |  |  |  | * |  | (53) |
| 859 |  |  |  | **NICU treatment measures** | Any antenatal steroid administration |  |  | * |  |  | (63) |
| 860 |  |  |  |  | Any antenatal steroid administration |  |  | * |  |  | (62) |
| 861 |  |  |  |  | Moderate hypothermia (36°C) on admission |  |  | * |  |  | (63) |
| 862 |  |  |  |  | Moderate hypothermia (36°C) on admission |  |  | * |  |  | (62) |
| 863 |  |  |  |  | Chronic lung disease (oxygen requirement at 36 weeks’ gestational age) |  |  | * |  |  | (63) |
| 864 |  |  |  |  | Chronic lung disease (oxygen requirement at 36 weeks’ gestational age) |  |  | * |  |  | (62) |
| 865 |  |  |  |  | Discharge on any human breast milk |  |  | * |  |  | (63) |
| 866 |  |  |  |  | Discharge on any human breast milk |  |  | * |  |  | (62) |
| **Row** | **Performance aspects** | **Category** | **Sub-category** | **Indicators** | **Sub-indicators** | **Input** | **Process** | **Output** | **Outcome** | **Effect** | **Ref** |
| 867 | **Appropriateness** | **Clinical Management** | **Service delivery and Treatment** | **NICU treatment measures** | Timely eye exam (retinopathy of prematurity screening at the age recommended by the American Academy of Pediatrics) |  |  | * |  |  | (63) |
| 868 |  |  |  |  | Timely eye exam (retinopathy of prematurity screening at the age recommended by the American Academy of Pediatrics) |  |  | * |  |  | (62) |
| 869 |  |  |  |  | Growth velocity (less or more than the median of 12.4 g/kg/day) |  |  | * |  |  | (63) |
| 870 |  |  |  |  | Growth velocity (less or more than the median of 12.4 g/kg/day) |  |  | * |  |  | (62) |
| 871 |  |  |  |  | Weight for gestational age below the 10th percentile |  |  | * |  |  | (62) |
| 872 |  |  |  |  | Weight for gestational age below the 10th percentile |  |  | * |  |  | (63) |
| 873 |  |  |  |  | Non–surgically-induced pneumothorax |  |  | * |  |  | (63) |
| 874 |  |  |  |  | Non–surgically-induced pneumothorax |  |  | * |  |  | (62) |
| 875 |  |  |  |  | Neonatologist coverage (in-house or at home) |  |  | * |  |  | (62) |
| 876 |  |  |  |  | minute Apgar score |  |  | * |  |  | (62) |
| 877 |  |  |  | **Drug and medication measures** | Number of verified medication orders |  |  | * |  |  | (64) |
| 878 |  |  |  |  | Percentage of accepted interventions |  |  | * |  |  | (64) |
| 879 |  |  |  |  | % of inpatients with a complete and accurate list of their current medications (including over-the-counter and complementary medications) documented and verified within a day of admission |  | * |  |  |  | (86) |
| 880 |  |  |  |  | % of inpatients with a correctly completed record (medication and reaction) of prior adverse drug reaction (ADR) and allergy documented within a day of admission |  | * |  |  |  | (86) |
| 881 |  |  |  |  | Intravenous antibiotics =Did patient receive one of the commonly used intravenous antibiotics during the ED visit? |  | * |  |  |  | (35) |
| 882 |  |  |  |  | Percentage conformity of drug types available at the pharmacy warehouse …..number of available drug types/number of drug types listed in the hospital formulary*100 |  | * |  |  |  | (71) |
| **Row** | **Performance aspects** | **Category** | **Sub-category** | **Indicators** | **Sub-indicators** | **Input** | **Process** | **Output** | **Outcome** | **Effect** | **Ref** |
| 883 | **Appropriateness** | **Clinical Management** | **Service delivery and Treatment** | **Drug and medication measures** | Percentage conformity of drug planning to actual use for each drug type=number of drug types in drug planning/number of drug types in real use*100 |  | * |  |  |  | (71) |
| 884 |  |  |  |  | Percentage conformity of drug type listed on the stock card vs. actual use=number of drug types listed on stock card/number of drug types in real use*100 |  | * |  |  |  | (71) |
| 885 |  |  |  |  | Percentage conformity of damaged or expired drugs=number of one-year-unused drug types/B = number of available drug types*100 |  |  |  | * |  | (71) |
| 886 |  |  |  |  | Percentage conformity of dead stock=number of damaged or expired drugs/number of stock-takings*100 |  |  |  | * |  | (71) |
| 887 |  |  |  |  | The percentage conformity of drugs serviced to the patients at the pharmacy warehouse=number of serviced drug types/number of prescribed drug type*100 |  |  | * |  |  | (71) |
| 888 |  |  |  |  | Percentage of patients with antibiotic prescribed respecting the national guidelines (%antibiotic-apc) |  |  |  | * |  | (38) |
| 889 |  |  |  |  | Percentage of patients with a dose of antibiotics prescribed respecting the national guidelines (%dose-apc) |  |  |  | * |  | (38) |
| 890 |  |  |  |  | Percentage of patients with antibiotic administered respecting the national guidelines (%apply-apc) |  |  |  | * |  | (38) |
| 891 |  |  |  |  | Percentage of patients with antibiotic therapy started respecting the national guidelines (%start-apc) |  |  |  | * |  | (38) |
| 892 |  |  |  |  | Percentage of patients with antibiotic therapy ended respecting the national guidelines (%end-apc) |  |  |  | * |  | (38) |
| 893 |  |  |  |  | Intravenous antiemetic= Did patient receive a costly intravenous anti-vomiting medication during the ED visit? |  |  |  | * |  | (35) |
| 894 |  |  |  |  | % of patients reviewed by a pharmacist within a day of admission |  |  |  | * |  | (86) |
| 895 |  |  |  |  | % of discharge summaries that document an accurate medication list and the reasons for all medication therapy changes from medications taken prior to admission |  |  |  | * |  | (86) |
| 896 |  |  |  |  | % of discharge prescriptions reviewed and reconciled by a pharmacist prior to dispensing |  | * |  |  |  | (86) |

| **Row** | **Performance aspects** | **Category** | **Sub-category** | **Indicators** | **Sub-indicators** | **Input** | **Process** | **Output** | **Outcome** | **Effect** | **Ref** |
| --- | --- | --- | --- | --- | --- | --- | --- | --- | --- | --- | --- |
| 897 | **Appropriateness** | **Clinical Management** | **Service delivery and Treatment** | **Drug and medication measures** | % of patients prescribed salbutamol on discharge who are given a written action plan for acute exacerbations of respiratory disease and a copy is communicated to the primary care clinician |  | * |  |  |  | (86) |
| 898 |  |  |  |  | % of hospital in patients who receive verbal counselling and/or written information about their medicines prior to discharge |  | * |  |  |  | (86) |
| 899 |  |  |  |  | % of patients commenced on warfarin during their admission who received counselling and written drug information prior to discharge |  | * |  |  |  | (86) |
| 900 |  |  |  |  | Number of guidelines and protocol reviews |  | * |  |  |  | (64) |
| 901 |  |  |  |  | Number of committee meetings attended |  | * |  |  |  | (64) |
| 902 |  |  |  |  | The percentage of appropriate prescriptions (%) |  |  |  | * |  | (22) |
| 903 |  |  |  |  | % of patients with an International Normalized Ratio result >4.0 whose dosage has been adjusted or reviewed prior to the next warfarin dose |  |  | * |  |  | (86) |
| 904 |  |  |  |  | % of patients with a toxic or sub therapeutic aminoglycoside concentration whose dosage has been adjusted or reviewed prior to the next aminoglycoside dose |  |  | * |  |  | (86) |
| 905 |  |  |  | **Obstetrics and Gynecology measures** | Normal deliveries =Total number of normal deliveries |  |  | * |  |  | (3) |
| 906 |  |  |  |  | Natural Delivery Portion (NDP) |  |  | * |  |  | (6) |
| 907 |  |  |  |  | Birth outside a regional center |  |  | * |  |  | (63) |
| 908 |  |  |  |  | Birth outside a regional center |  |  | * |  |  | (62) |
| 909 |  |  |  |  | At least 4 ANC recipients =Total number of women received 4 ANC services |  | * |  |  |  | (3) |
| 910 |  |  |  |  | PNC recipients= Total number of women received PNC service |  | * |  |  |  | (3) |
| 911 |  | **Administrative Management** | **Supportive Units Assessment** | **Nutritional measures** | Body weight |  | * |  |  |  | (33) |

| **Row** | **Performance aspects** | **Category** | **Sub-category** | **Indicators** | **Sub-indicators** | **Input** | **Process** | **Output** | **Outcome** | **Effect** | **Ref** |
| --- | --- | --- | --- | --- | --- | --- | --- | --- | --- | --- | --- |
| 912 | **Appropriateness** | **Administrative Management** | **Supportive Units Assessment** | **Nutritional measures** | Midarm and calf circumference |  | * |  |  |  | (33) |
| 913 |  |  |  |  | Serum albumin |  | * |  |  |  | (33) |
| 914 |  |  |  |  | Handgrip strength (HGS) |  | * |  |  |  | (33) |
| 915 |  |  |  |  | NIs at admission (SGA; SGA A = well nourished, SGA B =mild or moderate malnutrition, and SGA C = severe malnutrition) | * |  |  |  |  | (33) |
| 916 |  |  |  |  | Patient self-assessment of food intake |  | * |  |  |  | (33) |
| 917 | **Evaluation** | **Clinical Management** | **Service delivery and Treatment** | **ED Patients Acuity Levels** | Low acuity |  | * |  |  |  | (52) |
| 918 |  |  |  |  | Average ER Patients Acuity Level |  | * |  |  |  | (30) |
| 919 |  |  |  |  | Patient acuity level |  | * |  |  |  | (52) |
| 920 |  |  |  |  | Differential Percentages of ER Patients Acuity Levels |  | * |  |  |  | (30) |
| 921 |  |  | **Paramedical Assessment** | **Lab scoring** | Clinical chemistry laboratory scoring |  |  | * |  |  | (22) |
| 922 |  |  |  |  | Hematology laboratory scoring |  |  | * |  |  | (22) |
| 923 |  |  |  |  | Immunology laboratory scoring |  |  | * |  |  | (22) |
| 924 |  |  |  |  | Bacteriological laboratory scoring |  |  | * |  |  | (22) |
| 925 |  |  |  | **Imaging inspection** | Rate of CT inspection (%), ≥70% |  |  | * |  |  | (22) |
| 926 |  |  |  |  | Rate of MRI inspection (%), ≥70% |  |  | * |  |  | (22) |
| 927 |  |  |  |  | Rate of X-ray inspection (%), ≥70% |  |  | * |  |  | (22) |
| 928 |  |  |  | **Medication Use Evaluations (MUEs)** | % of admitted days that patients receive medication review by a pharmacist |  | * |  |  |  | (86) |
| 929 |  | **Clinical Management** | **Paramedical Assessment** |  | Number of medication reconciliations for new admissions |  | * |  |  |  | (64) |
| 930 |  |  |  |  | Number of medication use evaluations (MUEs) |  |  |  | * |  | (64) |
| 931 |  |  |  |  | Number of interventions during clinical rounds |  |  | * |  |  | (64) |

| **Row** | **Performance aspects** | **Category** | **Sub-category** | **Indicators** | **Sub-indicators** | **Input** | **Process** | **Output** | **Outcome** | **Effect** | **Ref** |
| --- | --- | --- | --- | --- | --- | --- | --- | --- | --- | --- | --- |
| 932 | **Evaluation** | **Clinical Management** | **Paramedical Assessment** | **Medication Use Evaluations (MUEs)** | Number of unapproved indication form evaluation |  |  |  | * |  | (64) |
| 933 |  | **Administrative Management** | **Supportive Units Assessment** | **Medical information measures** | Percent of total approvals to implement the decisions of the committee |  |  |  | * |  | (64) |
| 934 |  |  |  |  | The correct application of terminal digital |  |  |  | * |  | (64) |
| 935 |  |  |  |  | Completion rate of discharge summary |  |  |  | * |  | (2) |
| 936 |  |  |  |  | Percentage of class A medical records in all medical records (%), ≥95% |  |  |  | * |  | (22) |
| 937 |  |  |  |  | The percentage of appropriate written nursing documents (%) |  |  |  | * |  | (22) |
| 938 |  |  |  |  | Number of areas where electronic records and data are used=Are electronic records and data currently being used in your organization as a primary source of information? (Patient visit registration information; Diagnostic imaging reports; Electronic medical images; Diagnostic laboratory results; Patient-based pharmacy/drug profiles; Nursing and physician clinical documentation; Clinical documentation by other health professionals) |  |  |  | * |  | (59) |
| 939 |  |  |  |  | Medical Records Turnover |  |  | * |  |  | (64) |
| 940 |  | **Organizational Management** | **Hospital Characteristics** | **Hospital Rate** | The share of accredited hospitals (%) |  |  |  | * |  | (36) |
| 941 |  |  |  |  | Hospital rated 9-10 |  | * |  |  |  | (69) |
| 942 |  |  |  |  | Proportion of high complexity hospital outputs: The complexity of the patient is generally influenced by gender, age group, primary and secondary diagnoses, admission and output type and the need to perform surgical procedures. The complexity of the procedures performed, considered for this study, were only those as stated by the SUS Table of Procedures, Medicines, Orthotics, Prosthetics, and Special Materials. | * |  |  |  |  | (1) |
| 943 |  |  |  |  | Credit (Dummy)= The value is 1 if the hospital receives accreditation by Korea Institute for Healthcare Accreditation. Otherwise, the value is 0. |  |  |  | * |  | (55) |
| **Row** | **Performance aspects** | **Category** | **Sub-category** | **Indicators** | **Sub-indicators** | **Input** | **Process** | **Output** | **Outcome** | **Effect** | **Ref** |
| 944 | **Profitability** | **Organizational Management** | **Financial Management** | **Profit** | Net profit margin =(Total operating revenue-total operating expenses)/total operating revenue |  |  | * |  |  | (13) |
| 945 |  |  |  |  | Cash flow margin – [(net income - (contributions, investment and appropriations)) + depreciation expense + interest expense + amortization]/[net patient revenue + other income - (contributions, investments, and appropriations)] |  |  | * |  |  | (56) |
| 946 |  |  |  |  | Net profit per physician (PLN) |  |  | * |  |  | (36) |
| 947 |  |  |  |  | Modified EBITDA =The health care profits of each hospital adding back the expenditures of depreciation and amortization |  |  | * |  |  | (26) |
| 948 |  |  |  |  | Net profit margin =Total operating revenue-total operating expenses/total operating revenue |  |  | * |  |  | (16) |
| 949 |  |  |  |  | Profitability Index |  |  | * |  |  | (88) |
| 950 |  |  |  |  | Performance= Net Profit to Total Assets =(Net profit of this term /Total Assets) X 100 |  |  | * |  |  | (89) |
| 951 |  |  |  |  | Ratio of total revenue to total costs |  |  | * |  |  | (8) |
| 952 |  |  |  | **Margin ratio** | Operating margin= It indicates the profit generated per unit of sale and service provided after considering the production’s variable costs. An increasing value of this indicator shows that the entity is increasing its efficiency |  |  | * |  |  | (17) |
| 953 |  |  |  |  | Total margin =The percent by which a hospital's total revenue differs from its total expenses, excluding the impact of facility amortization (land, building and building service equipment). (Revenue – Expenses) * 100 / Revenues |  |  | * |  |  | (59) |
| 954 |  |  |  |  | Total margin - net income/total revenues; |  |  | * |  |  | (56) |
| 955 |  |  |  | **Assets ratios** | The Operating leverage= evaluates the impact of fixed costs on entity activity. The higher the value of fixed costs, the greater the entity rigidity and the higher its operational risk, since a large part of the contribution margin is absorbed by fixed costs. Thus, the higher this indicator, the greater the business risk. |  |  | * |  |  | (17) |
| 956 |  |  |  |  | Percentage of fixed assets in total assets (%) |  |  | * |  |  | (22) |

| **Row** | **Performance aspects** | **Category** | **Sub-category** | **Indicators** | **Sub-indicators** | **Input** | **Process** | **Output** | **Outcome** | **Effect** | **Ref** |
| --- | --- | --- | --- | --- | --- | --- | --- | --- | --- | --- | --- |
| 957 | **Profitability** | **Organizational Management** | **Financial Management** | **Assets ratios** | Fixed assets net value =The net fixed assets items in the public financial statement of each hospital. |  |  | * |  |  | (26) |
| 958 |  |  |  |  | The asset-liability ratio (%) |  |  | * |  |  | (22) |
| 959 |  |  |  |  | Efficiency- asset ratio |  |  | * |  |  | (88) |
| 960 |  |  |  |  | Asset-liability ratio |  |  | * |  |  | (21) |
| 961 |  |  |  |  | Return on Assets (ROA) reveals the entity’s performance in the period considered from its assets. ROA assesses the entity’s capacity to generate financial results through its assets. This indicator is computed before the impact of depreciation and amortization expenses, financing expenses, and income tax. Higher values of ROA indicate that the entity has a better performance in the use of its assets. |  |  | * |  |  | (17) |
| 962 |  |  |  | **Revenue/Income** | Patient revenue |  |  | * |  |  | (29) |
| 963 |  |  |  |  | Outpatient revenue |  |  | * |  |  | (29) |
| 964 |  |  |  |  | Income indices |  |  | * |  |  | (88) |
| 965 |  |  |  |  | EBIT/operating income =The impact of the cost structure (ROS) |  |  | * |  |  | (90) |
| 966 |  |  |  |  | Operating income (sales) / total assets =Activity indicator |  |  | * |  |  | (90) |
| 967 |  |  |  |  | Percentage of medicine income of the total income, ≤45% |  |  | * |  |  | (22) |
| 968 |  |  |  |  | Hospital revenue |  |  | * |  |  | (29) |
| 969 |  |  |  |  | Income generated by each staff member |  |  | * |  |  | (22) |
| 970 |  |  |  |  | Medical income per 100 Yuan of fixed assets |  |  | * |  |  | (22) |
| 971 |  |  |  |  | % of Drug income in business income |  |  | * |  |  | (21) |
| 972 |  |  |  |  | % of Examination income in medical income |  |  | * |  |  | (21) |
| 973 |  |  |  |  | Business income from per 100 RMB fixed assets |  |  | * |  |  | (21) |
| 974 |  |  |  |  | Medical income of the institution= Increase health insurance and the proportion of self-paid income |  |  | * |  |  | (82) |

| **Row** | **Performance aspects** | **Category** | **Sub-category** | **Indicators** | **Sub-indicators** | **Input** | **Process** | **Output** | **Outcome** | **Effect** | **Ref** |
| --- | --- | --- | --- | --- | --- | --- | --- | --- | --- | --- | --- |
| 975 | **Profitability** | **Organizational Management** | **Financial Management** | **Revenue/Income** | Self-pay income = For public hospitals, this is defined as [medical income–(medical expenses in the report of the hospitals’ health care service declaration status of the National Health Insurance Administration × regional point value)]; for private hospitals, it is defined as the non-health insurance income in detail files of health care income. |  |  | * |  |  | (26) |
| 976 |  |  |  |  | Increase ED revenue |  |  | * |  |  | (52) |
| 977 |  |  |  |  | % of Government grants in total income |  |  | * |  |  | (21) |
| 978 |  |  |  |  | Compensation as a percentage of total income (%) |  |  | * |  |  | (22) |
| 979 |  |  |  |  | Financial rewards= Average expenditure in financial rewards per total budget" |  |  | * |  |  | (13) |
| 980 | **Cost** | **Organizational Management** | **Financial Management** | **Budget allocation** | Percentage conformity of available budgets to required budgets=available budgets in drug planning/required budgets in drug planning *100 |  |  | * |  |  | (71) |
| 981 |  |  |  |  | Actual costs within budgeted costs |  |  | * |  |  | (65) |
| 982 |  |  |  |  | Actual costs within budgeted costs |  |  | * |  |  | (66) |
| 983 |  |  |  |  | Annual budget allocation for point of care test (KPI: adequate to fulfill all tests request) |  |  | * |  |  | (61) |
| 984 |  |  |  |  | Resource allocation to information technology=Percentage of the cost of new information systems purchased compared to the total budget |  |  | * |  |  | (13) |
| 985 |  |  |  |  | Percentage of budget used for purchase of new technology=Percentage of the cost of new technologies purchased compared to the total budget |  |  | * |  |  | (13) |
| 986 |  |  |  |  | Percentage conformity of allocated budgets for drug procurement=required budgets in drug procurement/available budgets in drug procurement*100 |  |  | * |  |  | (71) |
| 987 |  |  |  | **Personnel costs** | %Personnel costs of total costs |  |  | * |  |  | (8) |
| 988 |  |  |  |  | Personnel cost per total hours= Wage costs and employer costs divided by total hours |  |  | * |  |  | (48) |
| 989 |  |  |  |  | Wage costs= Direct wage costs of personnel (includes: nurses, anesthesiologists, and unit administration) |  |  | * |  |  | (48) |
| **Row** | **Performance aspects** | **Category** | **Sub-category** | **Indicators** | **Sub-indicators** | **Input** | **Process** | **Output** | **Outcome** | **Effect** | **Ref** |
| 990 | **Cost** | **Organizational Management** | **Financial Management** | **Personnel costs** | Employer costs= Social security premiums and other employer costs |  |  | * |  |  | (48) |
| 991 |  |  |  |  | Medical staff Cost=‘medical staff salary expenditures’ /‘medical staff cost’ |  |  | * |  |  | (29) |
| 992 |  |  |  |  | Non-medical staff cost= non-medical staff salary expenditures |  |  | * |  |  | (29) |
| 993 |  |  |  |  | Nursing staff cost= ‘nursing staff salary expenditures’ /‘nursing staff cost’ |  |  | * |  |  | (29) |
| 994 |  |  |  |  | % of Staff expenses in business expenditure |  |  | * |  |  | (21) |
| 995 |  |  |  |  | Personals expenses=Total of doctors, nurses, administrators and personals expenses |  |  | * |  |  | (13) |
| 996 |  |  |  |  | Personnel expenses per standard patient= Personnel expenses consider the governing bodies and staff remuneration and supplements, holidays, and Christmas allowances. Remuneration supplements include overtime. |  |  | * |  |  | (17) |
| 997 |  |  |  |  | Cost of employee benefits / operating income (sales) =Wage productivity |  |  | * |  |  | (90) |
| 998 |  |  |  |  | hourly cost of Medical Equipment technicians (internal and external) |  |  | * |  |  | (72) |
| 999 |  |  |  | **Non-personnel costs** | Non-medical non-nursing staff cost |  |  | * |  |  | (29) |
| 1000 |  |  |  |  | Increase in non-labor cost |  |  | * |  |  | (52) |
| 1001 |  |  |  |  | The cost of drugs and materials |  |  | * |  |  | (8) |
| 1002 |  |  |  |  | Material costs= Material costs from the income statement - cost of medicines, medical devices costs, costs of additional assortment in the pharmacy, the cost of blood |  |  | * |  |  | (51) |
| 1003 |  |  |  |  | Material costs= Costs of materials consumed at the unit (implant costs excluded) |  |  | * |  |  | (48) |
| 1004 |  |  |  |  | Medical material expenses= Total expenses of material medical |  |  | * |  |  | (13) |
| 1005 |  |  |  |  | Equipment expenses (in%) =How much a hospital spends in a given year to operate and maintain its computer systems, x-ray machines, and other capital equipment, compared to its total expenses. Equipment expense * 100 / Expenses |  |  | * |  |  | (59) |

| **Row** | **Performance aspects** | **Category** | **Sub-category** | **Indicators** | **Sub-indicators** | **Input** | **Process** | **Output** | **Outcome** | **Effect** | **Ref** |
| --- | --- | --- | --- | --- | --- | --- | --- | --- | --- | --- | --- |
| 1006 | **Cost** | **Organizational Management** | **Financial Management** | **Non-personnel costs** | Spare parts (and supplies) costs of Medical Equipment |  |  | * |  |  | (72) |
| 1007 |  |  |  |  | Service purchases= Costs of purchased services (e.g. imaging, laboratory diagnostics) |  |  | * |  |  | (48) |
| 1008 |  |  |  |  | Warehouse management cost (before outsourcing) |  |  | * |  |  | (24) |
| 1009 |  |  |  |  | Cost indices |  |  | * |  |  | (88) |
| 1010 |  |  |  |  | O&M cost per building area |  |  | * |  |  | (65) |
| 1011 |  |  |  |  | O&M cost per building area (HK$/m2) |  |  | * |  |  | (66) |
| 1012 |  |  |  |  | Energy cost per building area |  |  | * |  |  | (65) |
| 1013 |  |  |  |  | Energy cost per building area (HK$/m2) |  |  | * |  |  | (66) |
| 1014 |  |  |  |  | CM (and SM) expense as a percentage of total CE expense |  |  | * |  |  | (72) |
| 1015 |  |  |  |  | Facilities management cost per building area |  |  | * |  |  | (65) |
| 1016 |  |  |  |  | Maintenance cost per building area |  |  | * |  |  | (65) |
| 1017 |  |  |  |  | Drug expenses per standard patient. According to the Official Accountability Plan of the Health Ministry (POCMS), drugs represent all the products registered in the National Form of Drugs. This ratio expresses the expenses that a standard patient represents in terms of these products. Reduced values indicate a higher efficiency and productivity of the hospital since a standard patient represents a lower cost. |  |  | * |  |  | (17) |
| 1018 |  |  |  |  | Medicine expenses=Total expenses of medicine expenses |  |  | * |  |  | (13) |
| 1019 |  |  |  |  | Protection Against Catastrophic Expenditure |  |  | * |  |  | (49) |
| 1020 |  |  |  |  | Committed expenditure |  |  | * |  |  | (25) |
| 1021 |  |  |  |  | Percentage of expenditures in service revenue |  |  | * |  |  | (22) |
| 1022 |  |  |  |  | Medical research=Average expenditure on medical research |  |  | * |  |  | (13) |

| **Row** | **Performance aspects** | **Category** | **Sub-category** | **Indicators** | **Sub-indicators** | **Input** | **Process** | **Output** | **Outcome** | **Effect** | **Ref** |
| --- | --- | --- | --- | --- | --- | --- | --- | --- | --- | --- | --- |
| 1023 | **Cost** | **Organizational Management** | **Financial Management** | **Non-personnel costs** | The total RVUs for inpatient services= All outpatient medical expenses declared by the hospital (ex. Western medicine, Chinese medicine, dentist, dialysis, etc.), including application points and copayments in the second-generation storage and admission detail files of the NHI administration. |  |  | * |  |  | (26) |
| 1024 |  |  |  |  | Annual maintenance expenditure of total replacement value |  |  | * |  |  | (65) |
| 1025 |  |  |  |  | Total CE expense as a percentage of total cost of acquisition (cost of acquisition ratio) |  |  | * |  |  | (72) |
| 1026 |  |  |  |  | In-house (and external contracts) expense as a percentage of total CE expense |  |  | * |  |  | (72) |
| 1027 |  |  |  |  | Outpatient surgeries on potential outpatient procedures= Outpatient surgeries include scheduled surgical procedures carried out on an inpatient basis, in which the patient is admitted and discharged to her/his home on the day of the intervention or within a maximum of 24 h. This type of surgery represents a relevant instrument for increasing the hospital’s effectiveness, quality of care, and efficiency. Indeed, it allows not only the hospitalization dedication to situations more complicated but also health expenditure rationalization. |  |  | * |  |  | (17) |
| 1028 |  |  |  |  | Average age of plant -accumulated depreciation/depreciation expense |  |  | * |  |  | (56) |
| 1029 |  |  |  |  | Days cash on hand - (cash + short-term investments)/((total expenses-depreciation)/365) |  |  | * |  |  | (56) |
| 1030 |  |  |  |  | % of Public welfare expenses in total expenditure |  |  | * |  |  | (21) |
| 1031 |  |  |  |  | Ln Pay Count= All the medical care statements from health insurance expenses, medical aid expenses, and veterans expenses transformed by natural logarithm |  |  | * |  |  | (55) |
| 1032 |  |  |  |  | The amount of deductions due to defects in the MR |  |  | * |  |  | (64) |
| 1033 |  |  |  | **Average Costs** | Average Costs= Costs divided by total DRG-points |  |  | * |  |  | (46) |
| 1034 |  |  |  |  | Average costs of hospital treatment (PLN) |  |  | * |  |  | (36) |
| **Row** | **Performance aspects** | **Category** | **Sub-category** | **Indicators** | **Sub-indicators** | **Input** | **Process** | **Output** | **Outcome** | **Effect** | **Ref** |
| 1035 | **Cost** | **Organizational Management** | **Financial Management** | **Average Costs** | Average cost per weighted case =Measure of the ratio of a hospital acute inpatient care expenses to the number of acute inpatient cases weighted for the DRG complexity. The weighting enhances comparability across hospitals. The measure includes the percentage cost of hospital university staff financed by the regional administration for their patient care activity. This allows to take into account the overall hospital staff costs. |  |  | * |  |  | (42) |
| 1036 |  |  |  |  | Total costs= Sum of all cost items listed above |  |  | * |  |  | (48) |
| 1037 |  |  |  |  | Average expenditure per diagnostic imaging weighted for tariff= Measure of efficiency that compares costs and the value of the delivered diagnostic activity (sum of ambulatory tariffs). |  |  | * |  |  | (42) |
| 1038 |  |  |  |  | Cost per unit input |  |  | * |  |  | (18) |
| 1039 |  |  |  |  | Costs per bed= The total cost of income statement/beds |  |  | * |  |  | (51) |
| 1040 |  |  |  |  | Cost per weighted cas |  |  | * |  |  | (45) |
| 1041 |  |  |  |  | Hospital Cost= ‘hospital expenditures’ / ‘hospital cost’ /‘operating expenses’ / ‘medical expenditures, medical/pharmaceutical supplies |  |  | * |  |  | (29) |
| 1042 |  |  |  |  | Total cost per total OR hours= Total costs divided by total hours |  |  | * |  |  | (48) |
| 1043 |  |  |  |  | Internal costs =Costs of general administration allocated to the unit |  |  | * |  |  | (48) |
| 1044 |  |  |  |  | Operating expenses per standard patient. Operating expenses are all costs involved in the entity’s production process, excluding costs with drugs and staff. This ratio indicates the operating expenses that a standard patient represents. |  |  | * |  |  | (17) |
| 1045 |  |  |  |  | Other operating costs= Facility costs and maintenance |  |  | * |  |  | (48) |
| 1046 |  |  |  |  | Expenditure =Total expenditures of hospital other than labor cost (in thousand €) |  |  | * |  |  | (47) |
| 1047 |  |  |  |  | Expenses per inpatient |  |  | * |  |  | (21) |
| 1048 |  |  |  |  | Hospitalization expenses per day |  |  | * |  |  | (21) |
| 1049 |  |  |  |  | Expenses per outpatient |  |  | * |  |  | (21) |
| **Row** | **Performance aspects** | **Category** | **Sub-category** | **Indicators** | **Sub-indicators** | **Input** | **Process** | **Output** | **Outcome** | **Effect** | **Ref** |
| 1050 | **Cost** | **Organizational Management** | **Financial Management** | **Average Costs** | Training expenditures per capita |  |  | * |  |  | (8) |
| 1051 |  |  |  |  | Average outpatient expenditures |  |  | * |  |  | (22) |
| 1052 |  |  |  |  | Standard patient per expenses with supplies and external services. "External supplies and services" include "subcontracts" and "services." According to POCMS,17 "subcontracts" item includes the necessary work for the production process itself. There are three main accounts for supply and external services such as electricity, water, books, office supplies, representation expenses, communications, insurance, transportation, travel, litigation and notary services, publicity and advertising, cleaning, hygiene and comfort, and specialized jobs (food, laundry, computers, and others). Thus, this ratio indicates how many standard patients are included in the same cost with supplies and external services. |  |  | * |  |  | (17) |
| 1053 |  |  |  |  | Patient medical expenses =Per capita medical expenses for patients include hospitalization expenses, outpatient expenses, etc. |  |  | * |  |  | (16) |
| 1054 |  |  |  |  | Average hospitalization expenditures |  |  | * |  |  | (22) |
| 1055 |  |  |  |  | Average expenditures per bed per day |  |  | * |  |  | (22) |
| 1056 |  |  |  |  | Average expenditures per bed per day |  |  | * |  |  | (8) |
| 1057 | **Economy** | **Organizational Management** | **Financial Management** | **Financial Ratios** | Current ratio |  |  | * |  |  | (21) |
| 1058 |  |  |  |  | Quick ratio |  |  | * |  |  | (21) |
| 1059 |  |  |  |  | Rate of bed utilization |  |  | * |  |  | (21) |
| 1060 |  |  |  |  | Medical institution bed utilization ratio (%), ≥90% |  |  | * |  |  | (22) |
| 1061 |  |  |  |  | Other financial ratios |  |  | * |  |  | (88) |
| 1062 |  |  |  |  | Liquidity =(Liability/Total Assets) X 100 |  |  | * |  |  | (89) |
| 1063 |  |  |  |  | Turnover Ratio: Total Assets Turnover =(Operating Revenues)/Total Assets) X 100 |  |  | * |  |  | (89) |
| 1064 |  |  |  |  | Quick ratio (acid test) |  |  | * |  |  | (88) |
| **Row** | **Performance aspects** | **Category** | **Sub-category** | **Indicators** | **Sub-indicators** | **Input** | **Process** | **Output** | **Outcome** | **Effect** | **Ref** |
| 1065 | **Economy** | **Organizational Management** | **Financial Management** | **Financial Ratios** | Operating expense Ratio =Operating expenses to operating revenues ratio. Measure about management efficiency |  |  | * |  |  | (13) |
| 1066 |  |  |  |  | The Current liability ratio indicates whether the entity debt is mostly short or medium-long term. Values close to the unit reveal that most of the entity obligations are short-term, which is not favorable, as the entity may not (f4) Equity ratio. It indicates the extent to which the asset is financed by equity. That is, it reflects the financial strength and the entity’s ability to meet its non-current obligations. A low value of this indicator reflects the entity’s high dependence on third-party capital. |  |  | * |  |  | (17) |
| 1067 |  |  |  | **Financial Indices** | Exploitation indices |  |  | * |  |  | (88) |
| 1068 |  |  |  |  | Capital structure indices |  |  | * |  |  | (88) |
| 1069 |  |  |  |  | Liquidity Index |  |  | * |  |  | (88) |
| 1070 |  |  |  |  | % Deductions of hospital |  |  | * |  |  | (8) |
| 1071 |  |  |  |  | Growth Rate= Growth Rate of Inpatient, Outpatient, and Operating Revenue= [(current term - preceding term)/Net profit of preceding term] X 100 |  |  | * |  |  | (89) |
| 1072 |  |  |  |  | Productivity= Value Added Ratio to Gross Revenue= (value added /Operating Revenue) X 100) |  |  | * |  |  | (89) |
| 1073 |  |  |  |  | (Net profit +depreciation) / total debt =EAT to total debts |  |  | * |  |  | (90) |
| 1074 |  |  |  |  | Cash flow to total debt - (net income + depreciation expense)/(current liabilities + long-term debt); |  |  | * |  |  | (56) |
| 1075 |  |  |  |  | Market Competition |  |  | * |  |  | (58) |
| 1076 |  |  |  |  | Long-term debt to capitalization - long-term debt/(long term debt + owners’ equity); |  |  | * |  |  | (56) |
| 1077 |  |  |  |  | Surplus or deficit of appropriation = For public hospitals, it is the value of the remaining (short) items in the income and expenditure balance sheet of the current period; for private hospitals, it is the value of the after-tax items in the income and expenditure balance sheet of the current period. |  |  | * |  |  | (26) |

| **Row** | **Performance aspects** | **Category** | **Sub-category** | **Indicators** | **Sub-indicators** | **Input** | **Process** | **Output** | **Outcome** | **Effect** | **Ref** |
| --- | --- | --- | --- | --- | --- | --- | --- | --- | --- | --- | --- |
| 1078 | **Economy** | **Organizational Management** | **Financial Management** | **Financial Indices** | % of Management expenses in business expenditure |  |  | * |  |  | (21) |
| 1079 |  |  |  |  | Days in accounts receivable - net patient accounts receivable/(net patient service revenue/365) |  |  | * |  |  | (56) |
| 1080 |  |  |  |  | Capital |  |  | * |  |  | (29) |
| 1081 |  |  |  |  | Cash flow of the institution= Manage cash inflow and expenditures of medical institutions and the financial situations that have reached stable levels |  |  | * |  |  | (82) |
| 1082 |  |  |  |  | Economic added value= Vertical integration of medical institutions, referral of departments, and collective procurement of medical appliances and drugs |  |  | * |  |  | (82) |
| 1083 |  |  |  |  | Financial measures =Financial measures for healthcare organization, including cash-flow, profit margin, net operating margin, asset turnover, return on assets, etc. |  |  | * |  |  | (5) |
| 1084 |  |  |  |  | The average payment period indicates the average time elapsed, in days, between the goods and services purchase and the respective payment. High values of this indicator reveal that the entity has great negotiating capacity. Therefore, it can extend the payment period, or on the other hand, that it has difficulty in fulfilling its obligations. As such, it takes longer to settle them. |  |  | * |  |  | (17) |
| 1085 |  |  |  |  | The Current ratio reflects the ability to pay short-term obligations to current assets. The higher the value of this indicator, the better the hospital’s financial situation in the short term. Ideally, it should be higher than the unit. |  |  | * |  |  | (17) |
| 1086 |  |  |  |  | Return on Investment (ROI) indicates the leverage degree influence on results and return on equity. It allows assessing the possibility of taking advantage of financial leverage to increase the company’s results and profitability. The higher the value of ROI, the better the company’s performance in using its investments. |  |  | * |  |  | (17) |
| 1087 |  |  |  |  | Return on Sales (ROS)= It indicates the profit that is generated by each sales unit or service provided. The interpretation of this indicator is like the operating margin indicator (f6). |  |  | * |  |  | (17) |

| **Row** | **Performance aspects** | **Category** | **Sub-category** | **Indicators** | **Sub-indicators** | **Input** | **Process** | **Output** | **Outcome** | **Effect** | **Ref** |
| --- | --- | --- | --- | --- | --- | --- | --- | --- | --- | --- | --- |
| 1088 | **Economy** | **Organizational Management** | **Financial Management** | **Financial Indices** | The Solvability reveals the entity’s ability to settle its obligations with third parties. When taking the unit value, this indicator suggests that the entity has enough capital to cover its credits. |  |  | * |  |  | (17) |
| 1089 |  |  |  |  | Utilization of human resources ratio =Average percentage utilization of human resource |  |  | * |  |  | (13) |
| 1090 |  |  |  |  | Mean HAA value paid =represents the mean cost of each hospitalization. |  |  | * |  |  | (1) |
| 1091 |  |  |  |  | Gross domestic product (GDP) as non-controllable output |  |  | * |  |  | (43) |
| 1092 | **Coherence** | **Organizational Management** | **Financial Management** | **Discharge process** | Staff gave discharge information |  | * |  |  |  | (69) |
| 1093 |  |  |  |  | Heart failure patients given discharge instructions |  | * |  |  |  | (69) |
| 1094 | **Patient centeredness** | **Clinical Management** | **Service Recipients rights** | **Patient experience** | Doctors always communicated well |  | * |  |  |  | (58) |
| 1095 |  |  |  |  | Nurses always communicated well |  | * |  |  |  | (69) |
| 1096 |  |  |  |  | Communication with patient |  | * |  |  |  | (59) |
| 1097 |  |  |  |  | Communication with family |  | * |  |  |  | (59) |
| 1098 |  |  |  |  | Hospital always clean |  | * |  |  |  | (69) |
| 1099 |  |  |  |  | Hospital always quiet |  | * |  |  |  | (69) |
| 1100 |  |  |  |  | Patients always received help |  | * |  |  |  | (69) |
| 1101 |  |  |  |  | Overall rating Patient-centered care |  | * |  |  |  | (59) |
| 1102 |  |  |  |  | Patient experience (overall) |  | * |  |  |  | (45) |
| 1103 |  |  |  |  | Process of Care |  | * |  |  |  | (58) |
| 1104 |  |  |  |  | Technical Support |  | * |  |  |  | (58) |
| 1105 |  |  |  |  | % Alternate Level of Care (ALC) cases and days*) |  | * |  |  |  | (45) |
| 1106 |  |  |  |  | Continuous follow-up service for patients discharged from hospital= Continuous follow-up care services for patients after they have completed their medical treatment in order to increase the warm feeling of medical institutions |  |  |  | * |  | (82) |
| **Row** | **Performance aspects** | **Category** | **Sub-category** | **Indicators** | **Sub-indicators** | **Input** | **Process** | **Output** | **Outcome** | **Effect** | **Ref** |
| 1107 | **Equity** | **Clinical Management** | **Service Recipients rights** | **Health Equity** | Social equity = The percentage of the number of medical payment statements for patients under medical care and veterans care ... The ratio of payment statement of medical aid and veteran relief to total payment statements for each hospital |  |  |  | * |  | (55) |
| 1108 |  |  |  |  | Overall equity = The percentage of medical aid and veteran payments in total hospital medical payment...The ratio of payment of medical aid and veteran relief to total amount of medical payments for each hospital |  |  |  | * |  | (55) |
| 1109 |  |  |  |  | Equity ratio= It indicates the extent to which the asset is financed by equity. That is, it reflects the financial strength and the entity’s ability to meet its non-current obligations. A low value of this indicator reflects the entity’s high dependence on third-party capital. |  |  |  | * |  | (17) |
| 1110 |  |  |  |  | Return on Equity (ROE) denotes the capacity of the entity equity to generate a financial return. ROE evaluates the efficiency and capacity of investment management to produce financial results. The higher its value, the better the entity’s performance in the use of investments. |  |  |  | * |  | (17) |
| 1111 |  |  |  |  | Socio-economic equity= Socio-economic equity was assessed in relation to the five deprivation groups described above, and was assessed in the same way as for ethnic equity, with socio-economic groups replacing ethnic groups in calculations. |  |  |  | * |  | (41) |
| 1112 |  |  |  |  | Financial inequity= The percentage of out-of-pocket payments of the medical expenses for those with medical aid. The ratio of out-of-pocket payments over medical care expenses for the poor in the Medical Aid program for each hospital |  |  |  | * |  | (55) |

| **Row** | **Performance aspects** | **Category** | **Sub-category** | **Indicators** | **Sub-indicators** | **Input** | **Process** | **Output** | **Outcome** | **Effect** | **Ref** |
| --- | --- | --- | --- | --- | --- | --- | --- | --- | --- | --- | --- |
| 1113 | **Equity** | **Clinical Management** | **Service Recipients rights** | **Health Equity** | Ethnic equity=Ethnic equity was assessed for each hospital for the four efficiency and effectiveness indicators. To this end, scores for each indicator were calculated for each ethnic group within each hospital. For the efficiency indicators, scores were calculated by assessing (i) the relative stay index for each ethnic group within each hospital, relative to the expected length of stay for each hospital; and (ii) the standardized day surgery rate for each ethnic group within each hospital, and then expressed as a percentage of the average standardized day surgery rate across ethnic groups within each hospital. For the effectiveness indicators, adjusted rates of 30-day mortality and unplanned readmissions for each ethnic group in each hospital were calculated using the logistic regression models. These were then divided by the overall rate for each hospital to give an indication of the performance for each ethnic group on each indicator relative to the overall hospital performance for each indicator. An indicator of ethnic equity for each of the four indicators for each hospital was calculated as the coefficient of variation across ethnic groups. |  |  |  | * |  | (41) |
| 1114 |  |  |  |  | Years of potential lives lost |  |  |  | * |  | (24) |
| 1115 |  |  |  |  | Life expectancy Identified |  |  |  | * |  | (24) |
| 1116 |  |  |  |  | DSH Index (Disadvantaged Patient Populations) |  |  |  | * |  | (58) |
| 1117 |  |  |  |  | Poverty Rate |  |  |  | * |  | (58) |
| 1118 |  |  |  |  | Poverty headcount =Proportion of people living below the poverty line in the DHs area |  |  |  | * |  | (3) |
| 1119 |  |  |  |  | Population as non-controllable input |  |  |  | * |  | (43) |
| 1120 |  |  |  |  | Inequities following emergency admission according to social deprivation=Length of stay/Bed days(after emergency admission) |  |  |  | * |  | (53) |

| **Row** | **Performance aspects** | **Category** | **Sub-category** | **Indicators** | **Sub-indicators** | **Input** | **Process** | **Output** | **Outcome** | **Effect** | **Ref** |
| --- | --- | --- | --- | --- | --- | --- | --- | --- | --- | --- | --- |
| 1121 | **Relationship** | **Clinical Management** | **Service delivery and Treatment** | **Medical Consultations and Discussions** | Number of urgent consultations |  | * |  |  |  | (25) |
| 1122 |  |  |  |  | Number of elective consultations |  | * |  |  |  | (25) |
| 1123 |  |  |  |  | Number of pharmacokinetic consultations |  | * |  |  |  | (64) |
| 1124 |  |  |  |  | Number of total parenteral nutrition (TPN) consultations and follow-ups |  | * |  |  |  | (64) |
| 1125 |  |  |  |  | Number of discharge consultations |  | * |  |  |  | (64) |
| 1126 |  |  |  |  | Rate of Discussion on Risk for Blood Loss |  | * |  |  |  | (83) |
| 1127 |  |  |  |  | Rate of Discussion on Risk for Airway Difficulty/Aspiration |  | * |  |  |  | (83) |
| 1128 |  |  |  |  | Rate of Discussion on Sterility of Instruments and Equipment |  | * |  |  |  | (83) |
| 1129 |  |  |  |  | Rate of Surgeon’s Discussion on Patient-specific Concerns |  | * |  |  |  | (83) |
| 1130 |  |  |  |  | Rate of Anesthetist’s Discussion on Patient-Specific Concerns |  | * |  |  |  | (83) |
| 1131 |  |  |  |  | Rate of Surgeon’s Discussion on Anticipated Procedure level of Difficulty and Duration |  | * |  |  |  | (83) |
| 1132 |  |  |  |  | Rate of Discussion on Equipment/Instrument Problems During Surgery |  | * |  |  |  | (83) |
| 1133 |  |  |  |  | Rate of Discussion on Patient’s Post-op Recovery |  | * |  |  |  | (83) |
| 1134 | **Sustainability** | **Administrative Management** | **Supportive Units Assessment** | **Energy Management** | Energy use by zone |  |  | * |  |  | (67) |
| 1135 |  |  |  |  | Energy use in Outpatient zone |  |  | * |  |  | (67) |
| 1136 |  |  |  |  | Energy use in Inpatient zone |  |  | * |  |  | (67) |
| 1137 |  |  |  |  | Energy use in Public area zone |  |  | * |  |  | (67) |
| 1138 |  |  |  |  | Energy use in Central supply zone |  |  | * |  |  | (67) |
| 1139 |  |  |  |  | Energy use in Funeral services zone |  |  | * |  |  | (67) |
| 1140 |  |  |  |  | Electricity Energy use by zone |  |  | * |  |  | (67) |

| **Row** | **Performance aspects** | **Category** | **Sub-category** | **Indicators** | **Sub-indicators** | **Input** | **Process** | **Output** | **Outcome** | **Effect** | **Ref** |
| --- | --- | --- | --- | --- | --- | --- | --- | --- | --- | --- | --- |
| 1141 | **Sustainability** | **Administrative Management** | **Supportive Units Assessment** | **Energy Management** | District heat Energy use by zone |  |  | * |  |  | (67) |
| 1142 |  |  |  |  | LNG (Liquefied Natural Gas) Energy use by zone |  |  | * |  |  | (67) |
| 1143 |  |  |  |  | The annual water usage per bed (m3/bed/year) |  |  | * |  |  | (91) |
| 1144 |  |  |  |  | Per built area (m3/m2/year) for the different locations |  |  | * |  |  | (91) |
| 1145 |  |  |  |  | The covered area (ca) in square meters | * |  |  |  |  | (57) |
| 1146 |  |  |  |  | Power usage ratio of Lighting/plug systems |  |  | * |  |  | (67) |
| 1147 |  |  |  |  | Power usage ratio of HVAC (Heating Ventilation and Air Conditioning) systems |  |  | * |  |  | (67) |
| 1148 |  |  |  |  | Power usage ratio of Medical devices systems |  |  | * |  |  | (67) |
| 1149 |  |  |  |  | The energy yield ratio (EYR)=The EYR is the ratio between U, the total energy output given by R+N+F, and what purchased from the economy, F, thus indicating how much the energy output of a system actually depends on purchased resources, whose energy is in principle already available at the society level. It therefore addresses the question of how well the environmental resources are used for a given input from the economy, without distinguishing between renewables and nonrenewable. |  |  | * |  |  | (70) |
| 1150 |  |  |  |  | The environmental loading ratio (ELR)=The ELR=(F+N)/R indicator compares what is received from the economy plus local nonrenewable to the energy coming from local renewables. It indicates in general the level of environmental impact. |  |  | * |  |  | (70) |
| 1151 |  |  |  |  | The areal empower intensity (AEI) |  |  | * |  |  | (70) |
| 1152 |  |  |  |  | The renewable energy percentage (%Ren) |  |  |  | * |  | (70) |
| 1153 |  |  |  |  | Energy utilization index |  |  |  | * |  | (65) |
| 1154 |  |  |  |  | The energy sustainability index (ESI)=The Energy Sustainability Index, ESI=EYR/ELR, is an integrated measure of economic yield and environmental performance, and addresses the contribution to the overall support environment per unit load of the local system. |  |  |  | * |  | (70) |
| **Row** | **Performance aspects** | **Category** | **Sub-category** | **Indicators** | **Sub-indicators** | **Input** | **Process** | **Output** | **Outcome** | **Effect** | **Ref** |
| 1155 | **Sustainability** | **Administrative Management** | **Supportive Units Assessment** | **Energy Management** | Energy utilization index (EUI) (MJ/m2) |  |  |  | * |  | (66) |
| 1156 |  |  |  |  | Total energy on the base of TOE (Tonne of oil equivalent) |  |  | * |  |  | (67) |
| 1157 |  |  |  |  | Total energy on the base of kgoe/m2 (Kilogram of oil equivalent=kgoe) |  |  | * |  |  | (67) |
| 1158 |  |  |  |  | Carbon emissions per building area |  |  |  | * |  | (65) |
| 1159 |  |  |  |  | Carbon emissions per building area (tones’ CO2-e/m2) |  |  |  | * |  | (66) |
| 1160 | **Flexibility** | **Administrative Management** | **Supportive Units Assessment** | **Emergency response rate** | Volume flexibility rate= Based on the data of overcrowded patients. It represents number of times that the hospital can response to overcrowded situation per year |  | * |  |  |  | (13) |
| 1161 |  |  |  |  | Process flexibility rate =Number of cases that has been transferred to other hospital and time taken to transfer those cases to other hospital to number total of patient treated |  | * |  |  |  | (13) |

# References:

1. Ramos MCA, da Cruz LP, Kishima VC, Pollara WM, de Lira ACO, Couttolenc BF. Performance evaluation of hospitals that provide care in the public health system, Brazil. Rev Saude Publica. 2015;49.

2. Hung K-Y, Jerng J-S. Time to have a paradigm shift in health care quality measurement. J Formos Med Assoc. 2014;113(10):673–9.

3. Ahmed S, Hasan MZ, Laokri S, Jannat Z, Ahmed MW, Dorin F, et al. Technical efficiency of public district hospitals in Bangladesh: a data envelopment analysis. COST Eff Resour Alloc. 2019;17.

4. Shaqura II, Gholami M, Akbari Sari A. Assessment of public hospitals performance in Gaza governorates using the Pabón Lasso Model. Int J Health Plann Manage. 2021;36(4):1223–35.

5. Si S-L, You X-Y, Liu H-C, Huang J. Identifying Key Performance Indicators for Holistic Hospital Management with a Modified DEMATEL Approach. Int J Environ Res Public Health. 2017 Aug 19;14(8):934.

6. Sajadi HS, Sajadi ZS, Sajadi FA, Hadi M, Zahmatkesh M. The comparison of hospitals’ performance indicators before and after the Iran’s hospital care transformations plan. J Educ Health Promot. 2017;6:89.

7. Rahman MH, Tumpa TJ, Ali SM, Paul SK. A grey approach to predicting healthcare performance. Meas J Int Meas Confed. 2019;134:307–25.

8. Rahimi H, Bahmaei J, Shojaei P, Kavosi Z, Khavasi M. Developing a strategy map to improve public hospitals performance with balanced scorecard and dematel approach. Shiraz E Med J. 2018;19(7).

9. Jaskova D. EFFICIENCY OF MANAGEMENT PROCESSES IN A PRIVATE HOSPITAL. Entrep Sustain ISSUES. 2021;9(1):436–46.

10. Khalilabad T, Amir N, Asl P, Raeissi, Shali M, Niknam N. Assessment of clinical and paraclinical departments of military hospitals based on the Pabon Lasso Model. J Educ Health Promot. 2020 Apr 4;9.

11. Mehrtak M, Yusefzadeh H, Jaafaripooyan E. Pabon Lasso and Data Envelopment Analysis: a complementary approach to hospital performance measurement. Glob J Health Sci. 2014;6(4):107–16.

12. Mohammadi M, Ziapoor A, Mahboubi M, Faroukhi A, Amani N, Pour FH, et al. Performance evaluation of hospitals under supervision of Kermanshah medical sciences using pabonlasoty diagram of a five-year period (2008-2012). Life Sci J. 2014;11(1 SPECL. ISSUE):77–81.

13. Regragui H, Sefiani N, Azzouzi H. Improving performance through measurement: The application of BSC and AHP in healthcare organization. In Equipe de Recherche, Ingénierie, Innovation et Management des Systèmes Industriels, Université Abdelmalek Saadi, Tanger, Morocco: Institute of Electrical and Electronics Engineers Inc.; 2018. p. 51–6.

14. Aloh HE, Onwujekwe OE, Aloh OG, Nweke CJ. Is bed turnover rate a good metric for hospital scale efficiency? A measure of resource utilization rate for hospitals in Southeast Nigeria. COST Eff Resour Alloc. 2020;18(1).

15. Jebraeily M, Valizadeh MA, Rahimi B, Saeidi S. The Development of a Clinical Dashboard for Monitoring of Key Performance Indicators in ICU. J Iran Med Counc. 2022;5(2):308–17.

16. Zhang L, Liu R, Jiang S, Luo G, Liu H-C. Identification of Key Performance Indicators for Hospital Management Using an Extended Hesitant Linguistic DEMATEL Approach. Healthc (Basel, Switzerland). 2019 Dec 25;8(1):7.

17. Matos R, Ferreira D, Pedro MI. Economic Analysis of Portuguese Public Hospitals Through the Construction of Quality, Efficiency, Access, and Financial Related Composite Indicators. Soc Indic Res. 2021;157(1):361–92.

18. Aksezer CS. A nonparametric approach for optimal reliability allocation in health services. Int J Qual Reliab Manag. 2016;33(2):284–94.

19. Bosse G, Mtatifikolo F, Abels W, Strosing C, Breuer J-P, Spies C. Immediate Outcome Indicators in Perioperative Care: A Controlled Intervention Study on Quality Improvement in Hospitals in Tanzania. PLoS One. 2013;8(6).

20. De Sousa LR, Mazzo A, De Almeida ACF, Tonello C, Lourençone LFM. Evaluation of quality indicators in the management of an operating room at a tertiary-level hospital. Med. 2022;55(1).

21. Gao H, Chen H, Feng J, Qin X, Wang X, Liang S, et al. Balanced scorecard-based performance evaluation of Chinese county hospitals in underdeveloped areas. J Int Med Res. 2018;46(5):1947–62.

22. Li CH, Yu CH. Performance Evaluation of Public Non-Profit Hospitals Using a BP Artificial Neural Network: The Case of Hubei Province in China. Int J Environ Res Public Health. 2013;10(8):3619–33.

23. Pirani N, Zahiri M, Engali KA, Torabipour A. Hospital Efficiency Measurement Before and After Health Sector Evolution Plan in Southwest of Iran: a DEA-Panel Data Study. Acta Inform Med. 2018 Jun;26(2):106–10.

24. Craig KJT, McKillop MM, Huang HT, George J, Punwani ES, Rhee KB. US hospital performance methodologies: a scoping review to identify opportunities for crossing the quality chasm. BMC Health Serv Res. 2020;20(1).

25. Galloa AJO, Ramírez CA. Evaluating Colombian public hospitals productivity during 2004-2015. A Luenberger-Indicator approach. Rev Gerenc y Polit Salud. 2020;19.

26. Lin CS, Chiu CM, Huang YC, Lang HC, Chen MS. Evaluating the Operational Efficiency and Quality of Tertiary Hospitals in Taiwan: The Application of the EBITDA Indicator to the DEA Method and TOBIT Regression. HEALTHCARE. 2022;10(1).

27. Flatow VH, Ibragimova N, Divino CM, Eshak DSA, Twohig BC, Bassily-Marcus AM, et al. Quality Outcomes in the Surgical Intensive Care Unit after Electronic Health Record Implementation. Appl Clin Inform. 2015;6(4):611–8.

28. Drynda S, Schindler W, Slagman A, Pollmanns J, Horenkamp-Sonntag D, Schirrmeister W, et al. Evaluation of outcome relevance of quality indicators in the emergency department (ENQuIRE): study protocol for a prospective multicentre cohort study. BMJ Open. 2020;10(9).

29. Hadji B, Meyer R, Melikeche S, Escalon S, Degoulet P. Assessing the Relationships Between Hospital Resources and Activities: A Systematic Review. J Med Syst. 2014;38(10).

30. Khalifa M, Zabani I. Developing emergency room key performance indicators: What to measure and why should we measure it? J. M, A. H, P. G, A. K, M.S. H, editors. Vol. 226. King Faisal Specialist Hospital and Research Center, Jeddah, Saudi Arabia: IOS Press BV; 2016. p. 179–82.

31. Costa Jr A aS., Leão LE, Novais MA, Zucchi P. An assessment of the quality indicators of operative and non-operative times in a public university hospital. Einstein (Sao Paulo). 2015;13(4):594–9.

32. Schuers M, Joulakian MB, Griffon N, Pachéco J, Périgard C, Lepage E, et al. Quality indicators from laboratory and radiology information systems. A. G, I.N. S, P.M. de AM, editors. Vol. 216. Department of Biomedical Informatics, Rouen University Hospital, Rouen Cedex, 76031, France: IOS Press; 2015. p. 212–6.

33. Jeejeebhoy KN, Keller H, Gramlich L, Allard JP, Laporte M, Duerksen DR, et al. Nutritional assessment: Comparison of clinical assessment and objective variables for the prediction of length of hospital stay and readmission. Am J Clin Nutr. 2015;101(5):956–65.

34. Middleton S, Gardner G, Gardner A, Considine J, FitzGerald G, Christofis L, et al. Are service and patient indicators different in the presence or absence of nurse practitioners? the EDPRAC cohort study of Australian emergency departments. BMJ Open. 2019;9(7).

35. Liu K, Jain S, Shi J. Physician performance assessment using a composite quality index. Stat Med. 2013;32(15):2661–80.

36. Kocisova K, Hass-Symotiuk M, Kludacz-Alessandri M. USE OF THE DEA METHOD TO VERIFY THE PERFORMANCE MODEL FOR HOSPITALS. E M Ekon A Manag. 2018;21(4):125–40.

37. Niaksu O, Zaptorius J. Applying operational research and data mining to performance based medical personnel motivation system. In Vilnius University, Institute of Mathematics and Informatics, Lithuania: IOS Press; 2014. p. 63–70.

38. Kadoic N, Simic D, Mesaric J, Redep NB. Measuring Quality of Public Hospitals in Croatia Using a Multi-Criteria Approach. Int J Environ Res Public Health. 2021;18(19).

39. Petrovic GM, Vukovic M, Vranes AJ. The impact of accreditation on health care quality in hospitals. Vojnosanit Pregl. 2018;75(8):803–8.

40. Dolatabad AH, Mahdiraji HA, Babgohari AZ, Garza-Reyes JA, Ai A. Analyzing the key performance indicators of circular supply chains by hybrid fuzzy cognitive mapping and Fuzzy DEMATEL: evidence from healthcare sector. Environ Dev Sustain. 2022;

41. Davis P, Milne B, Parker K, Hider P, Lay-Yee R, Cumming J, et al. Efficiency, effectiveness, equity (E-3). Evaluating hospital performance in three dimensions. Health Policy (New York). 2013;112(1–2):19–27.

42. Nuti S, Grillo Ruggieri T, Podetti S. Do university hospitals perform better than general hospitals? A comparative analysis among Italian regions. BMJ Open. 2016;6(8).

43. Rabar D, Pap N. EVALUATION OF CROATIA’S REGIONAL HOSPITAL EFFICIENCY: AN APPLICATION OF DATA ENVELOPMENT ANALYSIS. Bacher U, Barkovic D, Dernoscheg KH, LamzaMaronic M, Matic B, Runzheimer B, editors. Vol. 9, INTERDISCIPLINARY MANAGEMENT RESEARCH IX. 2013. p. 649–59.

44. Córdoba S, Caballero I, Navalón R, Martínez-Sánchez D, Martínez-Morán C, Borbujo J. Analysis of the surgical activity in the Dermatology Department of Fuenlabrada University Hospital, Madrid, Spain, between 2005 and 2010: determination of the standard operating times. Actas Dermosifiliogr. 2013 Mar;104(2):141–7.

45. Backman C, Vanderloo S, Forster AJ. Measuring and improving quality in university hospitals in Canada: The Collaborative for Excellence in Healthcare Quality. Health Policy (New York). 2016;120(9):982–6.

46. Kittelsen SAC, Anthun KS, Goude F, Huitfeldt IMS, Häkkinen U, Kruse M, et al. Costs and quality at the hospital level in the nordic countries. Heal Econ (United Kingdom). 2015;24:140–63.

47. Xenos P, Yfantopoulos J, Nektarios M, Polyzos N, Tinios P, Constantopoulos A. Efficiency and productivity assessment of public hospitals in Greece during the crisis period 2009-2012. Cost Eff Resour Alloc. 2017;15(1).

48. Helkio P, Aantaa R, Virolainen P, Tuominen R. Productivity benchmarks for operative service units. ACTA Anaesthesiol Scand. 2016;60(4):450–6.

49. Bari S, Incorvia J, Iverson KR, Bekele A, Garringer K, Ahearn O, et al. Surgical data strengthening in Ethiopia: results of a Kirkpatrick framework evaluation of a data quality intervention. Glob Health Action. 2021;14(1).

50. Ramírez Calazans A, Paredes Esteban RM, Grijalva Estrada OB, Ibarra Rodríguez MR. Assessment of quality indicators in pediatric major outpatient surgery. Influence of the COVID-19 pandemic. Cir Pediatr. 2023;36(1):17–21.

51. Hajduová Z, Herbrik G, Beslerová S. Application of DEA in the environment of Slovak hospitals. Invest Manag Financ Innov. 2015;12(4):148–53 and 131.

52. Etu E-E, Monplaisir L, Aguwa C, Arslanturk S, Masoud S, Markevych I, et al. Identifying indicators influencing emergency department performance during a medical surge: A consensus-based modified fuzzy Delphi approach. PLoS One. 2022;17(4 April).

53. Morisod K, Luta X, Marti J, Spycher J, Malebranche M, Bodenmann P. Measuring Health Equity in Emergency Care Using Routinely Collected Data: A Systematic Review. Heal EQUITY. 2021;5(1):801–17.

54. Al-Jazairi AS, Alnakhli AO. Quantifying Clinical Pharmacist Activities in a Tertiary Care Hospital Using Key Performance Indicators. Hosp Pharm. 2021;56(4):321–7.

55. Kang Y, Kim M, Jung K. The Equity of Health Care Spending in South Korea: Testing the Impact of Publicness. Int J Environ Res Public Health. 2020;17(5).

56. Coyne JS, Helton J. How prepared are US hospitals for the affordable care act? A financial condition analysis of US hospitals in 2011. J Health Care Finance. 2015;41(3).

57. Dadi D, Introna V, Santolamazza A, Salvio M, Martini C, Pastura T, et al. Private Hospital Energy Performance Benchmarking Using Energy Audit Data: An Italian Case Study. Energies. 2022;15(3).

58. Cheon O, Song M, Mccrea AM, Meier KJ. Health Care in America: The Relationship Between Subjective and Objective Assessments of Hospitals. Int PUBLIC Manag J. 2021;24(5):596–622.

59. Koné Péfoyo AJ, Wodchis WP. Organizational performance impacting patient satisfaction in Ontario hospitals: a multilevel analysis. BMC Res Notes. 2013 Dec;6:509.

60. Bastos LSL, Hamacher S, Zampieri FG, Cavalcanti AB, Salluh JIF, Bozza FA. Structure and process associated with the efficiency of intensive care units in low-resource settings: An analysis of the CHECKLIST-ICU trial database. J Crit Care. 2020;59:118–23.

61. Nik Hisamuddin R, Tuan Hairulnizam TK. Developing Key Performance Indicators for Emergency Department of Teaching Hospitals: A Mixed Fuzzy Delphi and Nominal Group Technique Approach. MALAYSIAN J Med Sci. 2022;29(2):114–25.

62. Profit J, Gould JB, Bennett M, Goldstein BA, Draper D, Phibbs CS, et al. The association of level of care with NICU quality. Pediatrics. 2016;137(3).

63. Profit J, Kowalkowski MA, Zupancic JAF, Pietz K, Richardson P, Draper D, et al. Baby-MONITOR: A Composite Indicator of NICU Quality. Pediatrics. 2014;134(1):74–82.

64. Ajami S, Ebadsichani A, Tofighi S, Tavakoli N. Medical records department and balanced scorecard approach. J Educ Health Promot. 2013;2:7.

65. Lai J, Yuen PL. Identification, classification and shortlisting of performance indicators for hospital facilities management. FACILITIES. 2021;39(1–2):4–18.

66. Lai JHK, Hou H, Edwards DJ, Yuen PL. An analytic network process model for hospital facilities management performance evaluation. Facilities. 2022;40(5–6):333–52.

67. Hwang DK, Cho J, Moon J. Feasibility study on energy audit and data driven analysis procedure for building energy efficiency: Bench-marking in Korean hospital buildings. Energies. 2019;14(15).

68. Cagliano AC, Grimaldi S, Rafele C. Assessing warehouse centralization and outsourcing in the healthcare sector: An Italian case study. In Department of Management and Production Engineering, Politecnico di Torino, Corso Duca degli Abruzzi 24, Torino, 10129, Italy: AIDI - Italian Association of Industrial Operations Professors; 2017. p. 244–50.

69. Cefalu MS, Elliott MN, Setodji CM, Cleary PD, Hays RD. Hospital quality indicators are not unidimensional: A reanalysis of Lieberthal and Comer. Health Serv Res. 2019;54(2):502–8.

70. Cristiano S, Ulgiati S, Gonella F. Systemic sustainability and resilience assessment of health systems, addressing global societal priorities: Learnings from a top nonprofit hospital in a bioclimatic building in Africa. Renew Sustain ENERGY Rev. 2021;141.

71. Ghozali MT, Latifah DN, Darayani A. Analysis of Drug Supply Management of the Pharmacy Warehouse of Prof. Dr. Soerojo Mental Health Hospital, Magelang, Indonesia. Clin Schizophr Relat Psychoses. 2021;15.

72. Gonnelli V, Satta F, Frosini F, Iadanza E. Evidence-based approach to medical equipment maintenance monitoring. In: H. E, O. V, J. V, J. H, editors. University of Florence, Dept. of Information Engineering, Florence, Italy: Springer Verlag; 2017. p. 258–61.

73. Bhardwaj P, Joshi NK, Singh P, Suthar P, Joshi V, Jain YK, et al. Competence-Based Assessment of Biomedical Equipment Management and Maintenance System (e-Upkaran) Using Benefit Evaluation Framework. CUREUS J Med Sci. 2022;14(10).

74. Fekri O, Manukyan E, Klazinga N. Appropriateness, effectiveness and safety of care delivered in Canadian hospitals: A longitudinal assessment on the utility of publicly reported performance trend data between 2012-2013 and 2016-2017. BMJ Open. 2020;10(6).

75. Bastos LSL, Wortel SA, de Keizer NF, Bakhshi-Raiez F, Salluh JIF, Dongelmans DA, et al. Comparing continuous versus categorical measures to assess and benchmark intensive care unit performance. J Crit Care. 2022;70.

76. Nobakht S, Jahangiri K, Hajinabi K. Correlation of performance indicators and productivity: A cross sectional study of emergency departments in Tehran, Iran during year 2016. Trauma Mon. 2018;23(5).

77. Dawande PP, Wankhade RS, Akhtar FI, Noman O. Turnaround Time: An Efficacy Measure for Medical Laboratories. CUREUS J Med Sci. 2022;14(9).

78. Gebreyes M, Sisay A, Tegen D, Asnake A, Wolde M. Evaluation of Laboratory Performance, Associated Factors and Staff Awareness Towards Achieving Turnaround Time in Tertiary Hospitals, Ethiopia. Ethiop J Health Sci. 2020;30(5):767–76.

79. Abou Tarieh RR, Zayyat R, Naoufal RN, Samaha HR. A case study exploring the impact of JCI standards implementation on staff productivity and motivation at the laboratory and blood bank. Heal Sci REPORTS. 2022;5(1).

80. Gnanaraj J, Kulkarni RG, Sahoo D, Abhishekh B. Assessment of the Key Performance Indicator Proposed by NABH in the Blood Centre of a Tertiary Health Care Hospital in Southern India. Indian J Hematol Blood Transfus. 2022;

81. Tabrizi JS, Saadati M, Sadeghi-Bazargani H, Ebadi A, Golzari SEJ. Developing indicators to improve educational governance in hospitals. Clin Gov. 2014;19(2):117–25.

82. Lin C-Y, Shih F-C, Ho Y-H. Applying the Balanced Scorecard to Build Service Performance Measurements of Medical Institutions: An AHP-DEMATEL Approach. Int J Environ Res Public Health. 2023 Jan;20(2).

83. Bari S, Incorvia J, Ahearn O, Dara L, Sharma S, Varallo J, et al. Building safe surgery knowledge and capacity in Cambodia: a mixed-methods evaluation of an innovative training and mentorship intervention. Glob Health Action. 2021;14(1).

84. Fonseca JRS, Ramos RMP, Santos AMP, Fonseca APSS. Policy effects on the quality of public health care: Evaluating Portuguese public hospitals’ quality through customers’ views. Cent Eur J Public Policy. 2015;9(2):122–40.

85. Beta G, Role D, Berloviene D, Balkena Z. PATIENTS’ SATISFICATION AS THE QUALITY INDICATOR OF NURSING. Lubkina V, Kaupuzs A, Znotina D, editors. SOCIETY. INTEGRATION. EDUCATION, VOL VI: PUBLIC HEALTH AND SPORT, RESEARCHES IN ECONOMICS AND MANAGEMENT FOR SUSTAINABLE EDUCATION. 2020. p. 79–88.

86. Lloyd GF, Bajorek B, Barclay P, Goh S. Narrative review: Status of key performance indicators in contemporary hospital pharmacy practice. J Pharm Pract Res. 2015;45(4):396–403.

87. Horenberg F, Lungu DA, Nuti S. Measuring research in the big data era: The evolution of performance measurement systems in the Italian teaching hospitals. Health Policy (New York). 2020;124(12):1387–94.

88. Mirzaei A, Tabibi SJ, Nasiripour AA, Riahi L. Evaluating the Feasibility of Financial Variables of Health: A Hospital Administrator’s Viewpoint. GALEN Med J. 2016;5(1):25–30.

89. Lee D, Yu S, Yoon SN. Analysis of hospital management based on the characteristics of hospitals: Focusing on financial indicators. Glob Bus Financ Rev. 2019;24(3):1–13.

90. Váchová L, Hajdíková T. Evaluation of Czech hospitals performance using MCDM methods. In: S.I. A, W.S. G, C. D, editors. Department of Exact Methods, Faculty of Management, University of Economics, Prague, Jarošovská 1117, Jindřichuv Hradec, 37701, Czech Republic: Newswood Limited; 2017. p. 732–5.

91. Batista KJM, da Silva SR, Rabbani ERK, Zlatar T. Systematic review of indicators for the assessment of water consumption rates at hospitals. WATER SUPPLY. 2020;20(2):373–82.
