# Supplementary material for: Hospital performance evaluation indicators: a scoping review
Source: BMC Health Serv Res. 2024 May 1;24:561. doi: 10.1186/s12913-024-10940-1 (PMC11064245; doi:10.1186/s12913-024-10940-1)
Supplement: Supplementary file 2 — Supplementary Material 2. [file 12913_2024_10940_MOESM2_ESM.docx]

| **Appendix 2: The frequency used of the study performance aspects, categories and sub-categories** | | | | | | | | |
| --- | --- | --- | --- | --- | --- | --- | --- | --- |
| **Performance aspects** | | **No.** | **Categories** | | **No.** | **Sub-categories** | | **No.** |
| **Performance aspects frequency used** | Efficiency | 54 | **Category frequency used** | Organizational Management | 78 | **Sub-category frequency used** | Patient Safety | 46 |
|  | Productivity | 53 |  |  |  |  |  |  |
|  | Safety | 38 |  |  |  |  | Bed Utilization Rate | 46 |
|  | Effectiveness | 37 |  |  |  |  |  |  |
|  | Speed | 31 |  |  |  |  | Financial Management | 38 |
|  | Cost | 31 |  |  |  |  |  |  |
|  | Satisfaction | 19 |  |  |  |  | Human resource management | 31 |
|  | Appropriateness | 16 |  | Clinical Management | 62 |  |  |  |
|  | Profitability | 16 |  |  |  |  | Time Management | 31 |
|  | Economy | 16 |  |  |  |  |  |  |
|  | Quality of work life | 13 |  |  |  |  | Service delivery and Treatment | 29 |
|  | Quality | 12 |  |  |  |  |  |  |
|  | Evaluation | 12 |  |  |  |  | Supportive Units Assessment | 29 |
|  | Development | 11 |  |  |  |  |  |  |
|  | Equity | 8 |  | Administrative Management | 51 |  | Service Recipients rights | 29 |
|  | Sustainability | 6 |  |  |  |  | Resource management | 26 |
|  | Innovation | 5 |  |  |  |  | Medical Management | 16 |
|  | Patient centeredness | 5 |  |  |  |  | Quality Improvement | 14 |
|  | Relationship | 3 |  |  |  |  | Training and Education Management | 11 |
|  | Coherence | 1 |  |  |  |  | Hospital Characteristics | 10 |
|  | Flexibility | 1 |  |  |  |  | Paramedical Assessment | 6 |
